# The limited use of instructional design guidelines in healthcare simulation scenarios: an expert appraisal

**DOI:** 10.1186/s41077-022-00228-x

**Published:** 2022-09-24

**Authors:** Brena C. P. de Melo, Ana R. Falbo, Edvaldo S. Souza, Arno M. M. Muijtjens, Jeroen J. G. Van Merriënboer, Cees P. M. Van der Vleuten

**Affiliations:** 1 Centro de Atenção à Mulher, Instituto de Medicina Integral Prof. Fernando Figueira (IMIP), Recife, PE Brazil; 2 Centro de Simulação, Faculdade Pernambucana de Saúde (FPS), Av. Mascarenhas de Moraes, 4861, Imbiribeira, Recife, PE 51150-004 Brazil; 3 Diretoria de Pesquisa, Instituto de Medicina Integral Prof. Fernando Figueira (IMIP), Recife, PE Brazil; 4 Curso de Medicina, Faculdade Pernambucana de Saúde (FPS), Recife, PE Brazil; 5 School of Health Professions Education (SHE), Faculty of Health, Medicine and Life Sciences, Maastricht University, Maastricht, the Netherlands

**Keywords:** Simulation training, Instructional design guidelines, Postpartum hemorrhage

## Abstract

**Background:**

Systematic reviews on simulation training effectiveness have pointed to the need to adhere to evidence-based instructional design (ID) guidelines. ID guidelines derive from sound cognitive theories and aim to optimize complex learning (integration of knowledge, skills, and attitudes) and learning transfer (application of acquired knowledge and skills in the workplace). The purpose of this study was to explore adherence to ID guidelines in simulation training programs for dealing with postpartum hemorrhage (PPH), a high-risk situation and the leading cause of maternal mortality worldwide.

**Methods:**

A total of 40 raters analyzed simulation training programs as described in 32 articles. The articles were divided into four subsets of seven articles and one subset of four articles. Each subset was judged by seven to ten raters on adherence to ID guidelines. The 5-point Likert score rating scale was based on Merrill’s First Principles of Instruction and included items relating to key ID features categorized into five subscales: authenticity, activation of prior knowledge, demonstration, application, and integration/transfer. The authors searched for articles published in English between January 2007 and March 2017 in PubMed, Eric, and Google Scholar and calculated the mean Likert-scale score, per subscale, and interrater reliability (IRR).

**Results:**

The mean Likert-scale scores calculated for all subscales were < 3.00. For the number of raters used to judge the papers in this study (varying between 7 and 10), the IRR was found to be excellent for the authenticity and integration/transfer subscales, good-to-excellent for the activation of prior knowledge and application subscales, and fair-to-good for the demonstration subscale.

**Conclusion:**

The results demonstrate a paucity of the description of adherence to evidence-based ID guidelines in current simulation trainings for a high-risk situation such as PPH.

**Supplementary Information:**

The online version contains supplementary material available at 10.1186/s41077-022-00228-x.

## Background

Healthcare simulation training is a training strategy that is often recommended as a way of improving patient outcomes. It is thus often suggested for training high-risk situations such as postpartum hemorrhage (PPH) [1–5]. Achieving such improved outcomes, however, requires that a large number of interconnected elements be present, including the effectiveness of simulation training [6, 7]. Current literature provides much evidence of healthcare simulation training leading to positive learning outcomes [8, 9]. Evidence regarding transfer of learning (i.e., learners’ ability to apply the acquired knowledge and skills in the workplace subsequent to training) is, however, still being consolidated [10, 11]. Systematic reviews exploring the effectiveness of simulation training have underscored the importance of adherence to evidence-based instructional design guidelines as a conditioning factor related to the achievement of such transfer [12, 13].

Instructional design (ID) guidelines are based on sound learning theories and models and present a number of cognitive principles that aim to optimize complex learning and learning transfer [14, 15]. Complex learning concerns the proper integration of knowledge, skills, and attitudes, which is essential for the management of high-risk situations such as PPH [16]. Systematic reviews exploring the impact of simulation training on patient outcomes have already acknowledged the relevance of design features such as variability (clinical variation), repetitive practice of routine aspects, increasing complexity, mastery of learning (uniformly high achievement of standards), and providing feedback [7, 13, 17, 18].

The various ID guidelines available include Merrill’s First Principles of Instruction [19], which is a meta-model involving an overarching summary of available ID guidelines [20], proposing five key instructional principles for task-centered learning. These are based on careful analysis of a wide range of cognitive learning models: (1) identification of an *authentic problem* (since learning is promoted when learners are engaged with real-world problems), (2) *activation of prior knowledge* as the foundation for new knowledge, (3) *demonstration* of the task to be learned, (4) *application* of newly acquired knowledge by learners, and (5) *integration or transfer* of new knowledge into the learner’s world.

Simulation training has been widely advocated for PPH, the leading cause of maternal mortality worldwide, because most deaths related to this occurrence are attributable to management failures. To avoid such failures, which include delayed diagnosis, poor communication, and lack of adequate education and training, simulation training should be effective for both learning and transfer of learning [1, 5, 21–24].

Applying evidence-based ID guidelines to healthcare simulation training formats should be a priority when aiming to achieve transfer of learning and improve patient outcomes [10, 12]. This is of particular relevance for commonly encountered high-risk situations, such as PPH, in which achievement of adequate complex learning may be essential for maximizing patient safety [22, 25]. It was, therefore, necessary to explore the available literature for descriptions of the ID features used.

## Methods

The present study aimed to explore the extent to which articles in the literature describe simulation training programs for dealing with a high-risk situation—in this case PPH—as adhering to evidence-based ID guidelines.

We invited a panel of healthcare experts to appraise the use of evidence-based ID guidelines in PPH simulation training programs described in the literature by scoring the extent to which their use is described or lack of such description. We chose a particularly prevalent high-risk situation, PPH, as the training content to be analyzed, on account of its epidemiological importance [4], which has led to a widespread use of PPH simulation training programs. This study formed part of a broader research project on the use of instructional design guidelines in postpartum hemorrhage simulation training, which was submitted to and approved by the Institutional Review Board of the Instituto de Medicina Integral Prof. Fernando Figueira (IMIP), in Recife, Brazil, on March 17, 2012, CAE No. 0034.0.099.000-11.

### Participants

The participating raters were healthcare experts with a background in health education and, in particular, the training of health professionals. The raters were identified in two rounds and invited by email to collaborate. In the first round, from June 2015 to August 2015, we contacted authors and co-authors of previously published articles describing PPH simulation trainings. In the second round, from November 2016 to December 2016, we identified authors of abstracts listed in the Abstracts book of the International Association for Medical Education (AMEE) Conference 2016 with topics related to either simulation and/or instructional design. The corresponding contact information was located through Google Scholar profiles and similar webpages to confirm the authors’ backgrounds in health education and training expertise and to exclude undergraduate students. The raters contacted were asked to recommend other healthcare experts with a similar background who could also be invited. After both rounds, 98 raters were invited by email, of whom 60 agreed to participate and 40 returned the completed rating scales.

### Materials

The rating scale used for the analysis was based on Merrill’s First Principles of Instruction. Table 1 presents the complete list of the 24 rating-scale items, which were divided into the following five subscales: (1) authenticity, (2) activation of prior knowledge, (3) demonstration, (4) application, and (5) integration/transfer. Each item of each subscale was rated on a 5-point Likert scale: 1 = strongly disagree, 2 = disagree, 3 = neutral, 4 = agree, and 5 = strongly agree. If the corresponding item had not been described in the article reporting the PPH simulation training, raters could select a “not described” or “not applicable” option.

**Table 1:** Rating-scale items used for the analysis of articles, based on Merrill’s First Principles of Instruction

Subscales	Subitems
**Authenticity**	Scenarios are based on real-life tasks
	Trainees receive relevant theoretical information before they start to work on the scenario(s)
	Trainees receive guidance while they are working on the scenario(s)
	Scenarios differ from each other to the same extent as real-life tasks
	Scenarios are sequenced from simple to complex
	Trainees are encouraged to compare and contrast scenarios
**Activation of prior knowledge**	Trainees are required to activate their relevant prior knowledge and experience
	Trainees are encouraged to connect their past experience to new ideas, skills, and attitudes they are expected to learn
	Trainees receive a protocol that helps them to organize the new things they learn
	Trainees have the opportunity to demonstrate knowledge, skills, and attitudes they have already mastered before the training
**Demonstration**	Trainees are given demonstrations of the skills and/or models of the behaviors they are expected to learn
	Trainees are given examples of errors, mistakes, and things that can easily go wrong
	Trainees’ attention is directed to skills, information, and attitudes that are most relevant and/or important
	Trainees receive multiple demonstrations that represent alternative ways of performing the skills that need to be learned
	Trainees receive demonstrations not as simple descriptions but in a lifelike fashion (e.g., real-life modeling, video, animation)
	Trainees learn steps that contain non-observable decision-making and reasoning processes
**Application**	Trainees have opportunities to practice or try out what has been learned
	Trainees are tested on new scenarios to see if they can apply what has been learned
	Trainees’ errors when solving problems, doing learning tasks, or completing assignments are detected and they receive feedback on these
	Trainees are required to predict challenges and/or explain causes of undesirable outcomes
	Trainees collaborate with peers to enhance their learning
**Integration/transfer**	Trainees have the opportunity to reflect on, discuss with others, and defend what they have learned
	Trainees have the opportunity to explore how they can personally use what they learned
	Trainees are able to publicly demonstrate to others what they have learned

The rating scale was pre-tested in a pilot study with seven instruction experts who approved it for clarity.

**Fig. 1:**
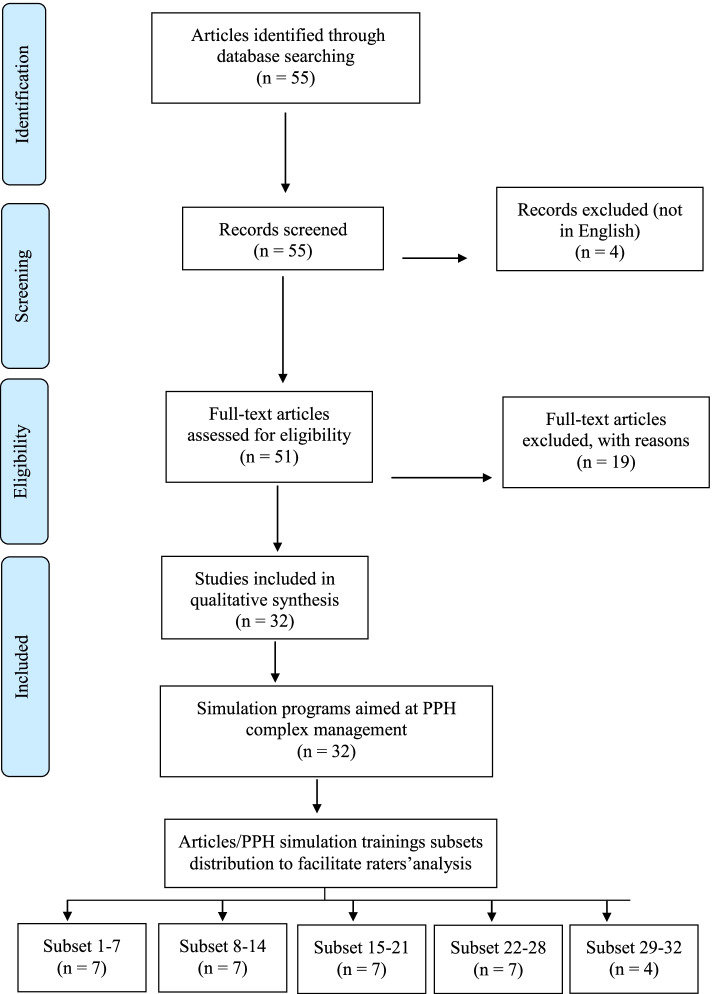
Flow diagram of the selection of the articles/PPH simulation trainings and subset distribution for raters

We analyzed PPH simulation training programs as described in articles identified by searching PubMed, Eric, and Google Scholar for studies published in English between January 2007 and March 2017, using the following keywords: “post-partum hemorrhage” AND “simulation” OR “simulation training” OR “medical simulation” OR “obstetric simulation.” We included studies retrieved by our keyword search and which described simulation training scenario(s) aimed at complex management of PPH that were attended by healthcare professionals. Articles were excluded if they lacked a description of PPH simulation training, provided secondary analysis of a PPH simulation scenario already described in one of the other articles, or described simulation scenarios intended for the training of specific individual PPH management-related skills. Our search yielded 51 studies, and after exclusion of 19 (10 for lacking a description of PPH simulation training itself, six for describing simulation scenarios for the training of specific individual PPH management-related skills, two conference abstracts, and one secondary analysis of a PPH simulation training already described in one of the other articles), the remaining 32 articles were analyzed. The remaining 32 articles were subdivided into the following five subsets to facilitate distribution for scoring by the raters: articles 1–7, 8–14, 15–21, 22–28, and 29–32. Figure 1 presents a flow diagram of the selection of the articles analyzed.

We prepared information tables for each article to facilitate analysis for raters. These contained the following information: article title, publication date, journal and publishing data, abstract, study design as described in the article, number of participants, and instructional aspects of the training. The selected articles were also carefully read, multiple times in full, searching for any description of the following training aspects of the PPH scenarios: presentation, practice, feedback, and assessment. These text segments were extracted and highlighted in the prepared tables as instructional aspects of the training. The full text of all the articles was also made available for consultation. Some of the raters reported consulting the full text of the articles only to confirm the absence of a description of one or more training aspects.

### Procedures

Upon agreeing to participate as a rater in the study, each rater received, by email, one of the subsets of articles for analysis along with the rating scale, distributed in a crossover fashion to avoid self-rating (for those who were both raters and authors of included articles). We also provided them with an instructional guide to help them understand the ID model to ensure that they were fully informed when assessing the articles. We also provided detailed instructions on how to fill out the rating scale (each item to be rated for each paper), and the corresponding subset information tables for the articles, which were sufficient for the analysis. All raters were invited to consult the authors by email, if necessary, and guidance was provided for any rater who did so. We have appended an additional file containing the complete set of instructions provided for raters (see Additional file 1).

We distributed the subsets of articles as soon as raters agreed to participate in the study and aimed to obtain an even number of final ratings. We consulted the raters regarding the feasibility of a 6-week deadline for returning the filled-out scales but were flexible about this when necessary. Of the 60 raters who agreed to participate, five declined to participate further in the study after receiving the materials for analysis, and 15 did not reply to subsequent attempts to contact them by email. A final total of 40 raters returned completed rating scales, constituting a response rate of 66.7%. The final numbers of raters scoring each subset of articles were as follows: subset 1–7 (eight raters), subset 8–14 (eight raters), subset 15–21 (seven raters), subset 22–28 (seven raters), and subset 29–32 (ten raters). Consequently, the data consisted of five blocks, each comprising the ratings of *N*_a_ (number of articles) articles by *N*_r_ (number of raters) raters, where *N*_a_ and *N*_r_ varied as indicated above. We chose to invite a large number of raters, as we expected significant variation in the scores given to the articles and wished ultimately to use mean scores as our primary measure.

### Statistical analysis

We used SPSS version 23 (IBM, Armonk, NY, USA) and Excel version 16.13.1 (Microsoft, Redmond, WA, USA) for data analysis. The first step of the analysis involved averaging item-specific scores for each article across all raters. The resulting article-level item scores were used as indicators of the article’s level of observed coverage of the items. In the aggregation, the “not described”/“not applicable” and missing answers were therefore recoded as “strongly disagree.” A resulting score < 3.00 thus indicated “little or no coverage observed.” In the following step, article-level subscale scores were obtained by calculating the average score of the corresponding items per subscale, thus providing indicators of an article’s level of coverage of Merrill’s First Principles (authenticity, activation of prior knowledge, demonstration, application, integration/transfer). The coverage of the subscales in the current sample of articles was explored by confirming a normal distribution and producing boxplots, M±SD, and percentiles.

Generalizability theory [26] was applied to the original intra-article rater data in order to estimate the interrater reliability (IRR) for each of the five subscales. We calculated the generalizability coefficient (*G*) as an estimation of reliability. In terms of generalizability theory, each of the five blocks has a so-called *a* × *r* design (ratings of *N*_a_ articles by *N*_r_ raters), and variance components (*V*) for article, rater, and article-rater interaction (*V*_a_, *V*_r_, and *V*_ar_, respectively) were obtained accordingly from each block of data. Taking the average of each component over the five blocks, a generalizability coefficient was calculated using the equation *G*=*V*_a_/(*V*_a_+*V*_ar_/*N*_r_), where *N*_r_ is the number of raters. The IRR is consequently higher for a block with more raters and, in the case of our data (with unequal *N*_r_ over blocks), we will thus find a range for the IRR over the five blocks. The IRR was calculated as indicated above for each of the five subscales. The resulting IRRs were qualified by applying the classifications proposed in Hallgren (2012) for intraclass correlation coefficients (ICCs) measuring IRR (of which *G* is an example).

## Results

Descriptive statistics for the subscale scores (5-point Likert) of the sample of articles (*N*=32) are shown in Table 2, which also provides the relative IRR (generalizability coefficient *G*) for each subscale.

**Table 2:** Subscale scores (5-point Likert) of articles (*N*=32) and relative interrater reliability (IRR) (generalizability coefficient *G*)

Subscale	Mean	Standard deviation	Percentiles			Relative IRR
			25th	50th (median)	75th	Generalizability coefficient (***G***)
**Authenticity**	2.62	.45	2.34	2.50	2.99	0.84–0.88
**Activation of prior knowledge**	2.64	.60	2.13	2.70	3.02	0.68–0.76
**Demonstration**	2.27	.36	2.02	2.26	2.51	0.56–0.65
**Application**	2.67	.46	2.43	2.66	2.91	0.73–0.79
**Integration/transfer**	2.60	.66	2.06	2.63	3.03	0.81–0.86

Further information on the selected articles is provided in Tables 3 and 4.

**Table 3:** Information on articles analyzed (author, year of publication, title, and brief description of the methodology)

Article #	Author, year	Title	Brief description of the methodology	
			Participants	Objective/methods
1	Andrighetti TP et al., 2012	Shoulder dystocia and postpartum hemorrhage simulations: student confidence in managing these complications	Registered nurses enrolled in a graduate midwifery education program	Quasiexperimental design evaluating student confidence
2	Brich L et al., 2007	Obstetric skills drills: evaluation of teaching methods	Junior and senior medical and midwifery staff	Three teaching methods were employed. Each team of staff was randomly allocated to undertake a full day of training
3	Chichester et al., 2014	A cost-effective approach to simulation-based team training in obstetrics.	Obstetric providers	Multidisciplinary learning experience
4	Clark et al., 2010	Team training/simulation	Obstetricians, anesthesiologists, midwives, nurses, pediatricians, and ancillary staff	An overview of team and simulation training
5	Cooper et al., 2012	Managing women with acute physiological deterioration: student midwives’ performance in a simulated setting	Student midwives	An exploratory quantitative analysis of student performance based upon performance ratings
6	Scholes et al., 2012	Clinical decision-making: midwifery students’ recognition of, and response to, postpartum hemorrhage in the simulation environment	Student midwives	Students were exposed to instruction on managing maternal deterioration and response to obstetric emergency as part of their curriculum program
7	Deering et al., 2009	Use of a postpartum hemorrhage simulator for instruction and evaluation of residents	Residents	Residents from 3 programs underwent training with a postpartum hemorrhage simulation
8	Egenberg et al., 2015	Can inter-professional simulation training influence the frequency of blood transfusions after birth?	All maternity staff	Two cohorts were compared retrospectively using a pre–post design
9	Fialkow et al., 2014	An in situ standardized patient-based simulation to train postpartum hemorrhage and team skills on a labor and delivery unit	Nurses, obstetrical residents, obstetrical attending physicians, anesthesiology residents, and anesthesiology attending physicians	Description of the development, content validation, and in situ implementation of a standardized patient-based, interdisciplinary PPH scenario
10	Magee et al., 2013	Low cost, high yield: simulation of obstetric emergencies for family medicine training.	Family medicine residents	Residents were randomly assigned to intervention or control group
11	Markova et al., 2012	Evaluation of multiprofessional obstetric skills training for postpartum hemorrhage	Midwives, nurses, auxiliary nurses, and doctors on call	A database audit
12	Marshal et al., 2014	Impact of simulation and team training on postpartum hemorrhage management in non-academic centers	Experienced clinical teams in non-academic hospitals in urban and rural communities	Multi-center longitudinal study to evaluate in situ simulation and team training for PPH
13	Maslovitz et al., 2007	Recurrent obstetric management mistakes identified by simulation	Residents in obstetrics and gynecology and midwives	To develop a simulation-based curricular unit for labor and delivery teams involved in obstetric emergencies to detect and address common mistakes
14	Maslovitz et al., 2008	Improved accuracy of postpartum blood loss estimation as assessed by simulation	Obstetrical teams consisted of physicians and obstetrical nurses	Prospective study conducted as part of the simulation-based training course to assess the accuracy of estimated blood loss by obstetrical teams during a simulated postpartum hemorrhage (PPH) scenario
15	Nelissen et al., 2014	Helping mothers survive bleeding after birth: an evaluation of simulation-based training in a low-resource setting	Clinicians, nurse-midwives, medical attendants, and ambulance drivers involved in maternity care	Educational intervention study
16	Phillippi et al., 2015	Interprofessional simulation of a retained placenta and postpartum hemorrhage	Students (nurse-midwifery, nursing students, and nurse-anesthesia students)	Interdisciplinary simulation designed jointly by the nurse-anesthesia and nurse-midwifery faculty to provide students with a realistic, complex experience to resolve an ongoing patient crisis
17	Robertson et al., 2009	Simulation-based crisis team training for multidisciplinary obstetric providers	Perinatal healthcare professionals (attending physicians, nurses, residents, and nurse midwives)	Pretest-posttest study design
18	Crofts et al., 2007	Change in knowledge of midwives and obstetricians following obstetric emergency training: a randomised controlled trial of local hospital, simulation centre and teamwork training	Midwives (including those working in hospital or the community) and all doctors, working within the Obstetric Department (including general practice trainees, obstetrics and gynecology trainees, and consultants)	Prospective randomized controlled trial, as part of the wider Simulation and Fire-drill Evaluation (SaFE) study
19	Siassakos et al., 2009	Content analysis of team communication in an obstetric emergency scenario	Doctors and midwives	Assess the utility, content validity, and application of techniques used in aviation, for the qualitative analysis of team communication in a “low fidelity” simulated obstetric emergency scenario before and after clinical training
20	Straub et al., 2013	Targeted obstetric hemorrhage program improves incoming resident confidence and knowledge	Incoming obstetrics and gynecology (OB) and family medicine (FM) residents	An educational program consisting of a lecture and high-fidelity simulation exercise
21	Vadnais et al., 2012	Assessment of long-term knowledge retention following single-day simulation training for uncommon but critical obstetrical events	Resident and attending physicians	Pretest-postest study design 4 and 12 months later
22	Kato et al., 2017	Simulation training program for midwives to manage postpartum hemorrhage: a randomized controlled trial	Midwives	RCT comparing simulation training group versus no training group using a pretest-intervention-posttest design
23	Melo et al., 2017	The use of instructional design guidelines to increase effectiveness of postpartum hemorrhage simulation training	Obstetrics and gynecology residents	Pretest–post-test non-equivalent groups study
24	Egenberg et al., 2016	Changes in self-efficacy, collective efficacy, and patient outcome following interprofessional simulation training on postpartum hemorrhage	Midwives, obstetricians, and auxiliary nurses	The study had a multimethod, quasi-experimental pre-post design that combined patient outcome with survey measures
25	Nathan et al., 2016	Retention of skills 2 years after completion of a postpartum hemorrhage simulation training program in rural Rwanda	Rural physicians	A quasi-experimental, pre–post-intervention study
26	Higgins et al., 2015	Teaching an experienced multidisciplinary team about postpartum hemorrhage: comparison of two different methods	Experienced clinicians	This study compared the impressions of experienced clinicians on the effect of two methods of educational interventions in a More^OB^ training program designed to improve recognition and management of PPH
27	Hilton et al., 2015	Checklists and multidisciplinary team performance during simulated obstetric hemorrhage	Multidisciplinary teams	Prospective observational study
28	Miller et al., 2015	Emergency birth hybrid simulation with standardized patients in midwifery education: implementation and evaluation	Graduate midwives	This article describes the development and initial evaluation of hybrid simulation used for labor and birth emergency situations
29	Wong et al., 2015	The state of Illinois obstetric hemorrhage project: pre-project and post-training examination scores	Physicians, registered nurses, advanced practice nurses	To describe the implementation of the OBHEP project and to report on change and retention in knowledge among providers, as assessed by the pre- and post-tests
30	Evans et al., 2014	Competency-based training “Helping Mothers Survive: Bleeding after Birth” for providers from central and remote facilities in three countries	Skilled and semiskilled birth attendants	A pre- and post-assessment of participants in BAB (bleeding after birth) training
31	Monod et al., 2014	Optimization of competency in obstetrical emergencies: a role for simulation training	Midwives and obstetricians	Observational study
32	Highfield et al., 2016	Effect of nurse-led simulation on OB/perinatal nurses’ knowledge & confidence in managing complications & emergencies	Registered nurses	Pre-/posttest study

**Table 4:** List of rated articles with full references

Article #	Author, year	Complete reference
1	Andrighetti TP et al., 2012	Andrighetti TP, Knestrick JM, Marowitz A, Martin C, Engstrom JL. Shoulder dystocia and postpartum hemorrhage simulations: student confidence in managing these complications. J Midwifery Women’s Health 2012;57:55-60. doi: 10.1111/j.1542-2011.2011.00085.x. Epub 2011 Sep 23.
2	Brich L, et al., 2007	Birch L, Jones N, Doyle PM, Green P, McLaughlin A, Champney C, Williams D, Gibbon K, Taylor K. Obstetric skills drills: evaluation of teaching methods. Nurse Educ Today 2007;27:915-22. doi: 10.1016/j.nedt.2007.01.006. Epub 2007 Mar 21.
3	Chichester et al., 2014	Chichester M, Hall NJ, Wyatt TL, Pomilla R. A cost-effective approach to simulation-based team training in obstetrics. Nurs Women’s Health 2014;18:500-7. doi: 10.1111/1751-486X.12162.
4	Clark et al., 2010	Clark EA, Fisher J, Arafeh J, Druzin M. Team training/simulation. Clin Obstet Gynecol 2010;53:265-77. doi: 10.1097/GRF.0b013e3181cc4595.
5	Cooper et al., 2012	Cooper S, Bulle B, Biro MA, Jones J, Miles M, Gilmour C, Buykx P, Boland R, Kinsman L, Scholes J, Endacott R. Managing women with acute physiological deterioration: student midwives performance in a simulated setting. Women Birth 2012;25:e27-36. doi: 10.1016/j.wombi.2011.08.009. Epub 2011 Sep 22.
6	Scholes et al., 2012	Scholes J, Endacott R, Biro M, Bulle B, Cooper S, Miles M, Gilmour C, Buykx P, Kinsman L, Boland R, Jones J, Zaidi F. Clinical decision-making: midwifery students' recognition of, and response to, post partum haemorrhage in the simulation environment. BMC Pregnancy Childbirth 2012;12:19. doi: 10.1186/1471-2393-12-19.
7	Deering et al., 2009	Deering SH, Chinn M, Hodor J, Benedetti T, Mandel LS, Goff B. Use of a postpartum hemorrhage simulator for instruction and evaluation of residents. J Grad Med Educ 2009;1:260-3. doi: 10.4300/JGME-D-09-00023.1.
8	Egenberg et al., 2015	Egenberg S, Øian P, Bru LE, Sautter M, Kristoffersen G, Eggebø TM. Can inter-professional simulation training influence the frequency of blood transfusions after birth? Acta Obstet Gynecol Scand 2015;94:316-23. doi: 10.1111/aogs.12569. Epub 2015 Feb 1.
9	Fialkow et al., 2014	Fialkow MF, Adams CR, Carranza L, Golden SJ, Benedetti TJ, Fernandez R. An in situ standardized patient-based simulation to train postpartum hemorrhage and team skills on a labor and delivery unit. Simul Healthc 2014;9:65-71. doi: 10.1097/SIH.0000000000000007.
10	Magee et al., 2013	Magee SR, Shields R, Nothnagle M. Low cost, high yield: simulation of obstetric emergencies for family medicine training. Teach Learn Med 2013;25:207-10. doi: 10.1080/10401334.2013.797353.
11	Markova et al., 2012	Markova V, Sørensen JL, Holm C, Nørgaard A, Langhoff-Roos J. Evaluation of multi-professional obstetric skills training for postpartum hemorrhage. Acta Obstet Gynecol Scand 2012;91:346-52. doi: 10.1111/j.1600-0412.2011.01344.x.
12	Marshal et al., 2014	Marshall NE, Vanderhoeven J, Eden KB, Segel SY, Guise JM. Impact of simulation and team training on postpartum hemorrhage management in non-academic centers. J Matern Fetal Neonatal Med 2015;28:495-9. doi: 10.3109/14767058.2014.923393. Epub 2014 May 29.
13	Maslovitz et al., 2007	Maslovitz S, Barkai G, Lessing JB, Ziv A, Many A. Recurrent obstetric management mistakes identified by simulation. Obstet Gynecol 2007;109:1295-300. doi: 10.1097/01.AOG.0000265208.16659.c9.
14	Maslovitz et al., 2008	Maslovitz S, Barkai G, Lessing JB, Ziv A, Many A. Improved accuracy of postpartum blood loss estimation as assessed by simulation. Acta Obstet Gynecol Scand 2008;87:929-34. doi: 10.1080/00016340802317794.
15	Nelissen et al., 2014	Nelissen E, Ersdal H, Ostergaard D, Mduma E, Broerse J, Evjen-Olsen B, van Roosmalen J, Stekelenburg J. Helping mothers survive bleeding after birth: an evaluation of simulation-based training in a low-resource setting. Acta Obstet Gynecol Scand 2014;93:287-95. doi: 10.1111/aogs.12321. Epub 2014 Jan 15.
16	Phillippi et al., 2015	Phillippi JC, Buxton M, Overstreet M. Interprofessional simulation of a retained placenta and postpartum hemorrhage. Nurse Educ Pract 2015;15:333-8. doi: 10.1016/j.nepr.2015.02.001. Epub 2015 Feb 14.
17	Robertson et al., 2009	Robertson B, Schumacher L, Gosman G, Kanfer R, Kelley M, DeVita M. Simulation-based crisis team training for multidisciplinary obstetric providers. Simul Healthc 2009;4:77-83. doi: 10.1097/SIH.0b013e31819171cd.
18	Crofts et al., 2007	Crofts JF, Ellis D, Draycott TJ, Winter C, Hunt LP, Akande VA. Change in knowledge of midwives and obstetricians following obstetric emergency training: a randomised controlled trial of local hospital, simulation centre and teamwork training. BJOG 2007;114:1534-41. doi: 10.1111/j.1471-0528.2007.01493.x. Epub 2007 Sep 27.
19	Siassakos et al., 2009	Siassakos D, Draycott T, Montague I, Harris M. Content analysis of team communication in an obstetric emergency scenario. J Obstet Gynaecol 2009;29:499-503. doi: 10.1080/01443610903039153.
20	Straub et al., 2013	Straub HL, Morgan G, Ochoa P, Grable I, Wang E, Kharasch M, Plunkett BA. Targeted obstetric haemorrhage programme improves incoming resident confidence and knowledge. J Obstet Gynaecol 2013;33:798-801. doi: 10.3109/01443615.2013.816668.
21	Vadnais et al., 2012	Vadnais MA, Dodge LE, Awtrey CS, Ricciotti HA, Golen TH, Hacker MR. Assessment of long-term knowledge retention following single-day simulation training for uncommon but critical obstetrical events. J Matern Fetal Neonatal Med 2012;25:1640-5. doi: 10.3109/14767058.2011.648971. Epub 2012 Apr 25.
22	Kato et al., 2017	Kato C, Kataoka Y. Simulation training program for midwives to manage postpartum hemorrhage: A randomized controlled trial. Nurse Educ Today 2017;51:88-95. doi: 10.1016/j.nedt.2017.01.005. Epub 2017 Jan 20.
23	Melo et al., 2017	de Melo BC, Falbo AR, Muijtjens AM, van der Vleuten CP, van Merriënboer JJ. The use of instructional design guidelines to increase effectiveness of postpartum hemorrhage simulation training. Int J Gynaecol Obstet 2017;137:99-105. doi: 10.1002/ijgo.12084. Epub 2017 Jan 16.
24	Egenberg et al., 2016	Egenberg S, Øian P, Eggebø TM, Arsenovic MG, Bru LE. Changes in self-efficacy, collective efficacy and patient outcome following interprofessional simulation training on postpartum haemorrhage. J Clin Nurs 2017;26:3174-3187. doi: 10.1111/jocn.13666. Epub 2017 Mar 12.
25	Nathan et al., 2016	Nathan LM, Patauli D, Nsabimana D, Bernstein PS, Rulisa S, Goffman D. Retention of skills 2 years after completion of a postpartum hemorrhage simulation training program in rural Rwanda. Int J Gynaecol Obstet 2016;134:350-3. doi: 10.1016/j.ijgo.2016.01.021. Epub 2016 May 16.
26	Higgins et al., 2015	Higgins M, Kfouri J, Biringer A, Seaward G, Windrim R. Teaching an Experienced Multidisciplinary Team About Postpartum Hemorrhage: Comparison of Two Different Methods. J Obstet Gynaecol Can 2015;37:824-828. doi: 10.1016/S1701-2163(15)30155-9.
27	Hilton et al., 2015	Hilton G, Daniels K, Goldhaber-Fiebert SN, Lipman S, Carvalho B, Butwick A. Checklists and multidisciplinary team performance during simulated obstetric hemorrhage. Int J Obstet Anesth 2016;25:9-16. doi: 10.1016/j.ijoa.2015.08.011. Epub 2015 Aug 21.
28	Miller et al., 2015	Lindsay Miller J, Avery MD, Larson K, Woll A, VonAchen A, Mortenson A. Emergency birth hybrid simulation with standardized patients in midwifery education: implementation and evaluation. J Midwifery Women’s Health 2015;60:298-303. doi: 10.1111/jmwh.12276. Epub 2015 May 11.
29	Wong et al., 2015	Wong CA, Scott S, Jones RL, Walzer J, Geller S. The state of Illinois obstetric hemorrhage project: pre-project and post-training examination scores. J Matern Fetal Neonatal Med 2016;29:845-9. doi: 10.3109/14767058.2015.1021672. Epub 2015 Sep 4.
30	Evans et al., 2014	Evans CL, Johnson P, Bazant E, Bhatnagar N, Zgambo J, Khamis AR. Competency-based training “Helping Mothers Survive: Bleeding after Birth” for providers from central and remote facilities in three countries. Int J Gynaecol Obstet. 2014;126:286-90. doi: 10.1016/j.ijgo.2014.02.021. Epub 2014 Apr 24.
31	Monod et al., 2014	Monod C, Voekt CA, Gisin M, Gisin S, Hoesli IM. Optimization of competency in obstetrical emergencies: a role for simulation training. Arch Gynecol Obstet 2014;289:733-8. doi: 10.1007/s00404-013-3111-6. Epub 2013 Dec 18.
32	Highfield et al., 2016	Farrar Highfield ME, Scharf-Swaller C, Chu L. Effect of Nurse-Led Review Plus Simulation on Obstetric/Perinatal Nurses' Self-Assessed Knowledge and Confidence. Nurs Womens Health 2017;20:568-581. doi: 10.1016/j.nwh.2016.10.007.

For all subscales, the mean scores were found to be lower than 2.68, with more than 75% of the item scores below 3.04 and over 50% below 2.71. These findings indicate that the raters noted a paucity of description of aspects relating to adherence to evidence-based ID guideline aspects in a large majority of the PPH simulation training programs. For the authenticity, activation of prior knowledge, application, and integration/transfer subscales, the IRR varied between 0.68 and 0.88, which we considered to represent “good to excellent” agreement for the purposes of the present study. The IRR for the demonstration subscale was 0.56–0.65, which is not fully acceptable for our purposes.

## Discussion

Our Likert-scale mean scores were below the neutral score of 3 for all subscales. This indicates a pervasive lack of description of adherence to the main principles of evidence-based ID guidelines in simulation training for high-risk situations such as PPH. Our findings for four of the subscales — authenticity, integration/transfer, activation of prior knowledge, and application — are particularly worthy of note. These subscales presented IRR values ranging from good to excellent. The IRR level found for the demonstration subscale may be the result of incomplete or missing descriptions of the ID features relating to this subscale.

The raters’ overall agreement on the lack of coverage of evidence-based ID guidelines for almost all subscales reveals a lack of adequate description of the use of relevant ID features in PPH simulation training. Such lack of adequate description raises concern regarding the appropriate use of relevant ID features and the potentially detrimental effect of this on the transfer of learning. The proper description should involve reporting guidelines and the latter should, also for the sake of transfer of learning, present, in detail, key elements of evidence-based ID guidelines [27–29].

We can only speculate as to the reasons underlying this paucity of an adequate description of the use of evidence-based ID guidelines by those who promote simulation. The large body of sound evidence available as to the potentially detrimental effects on learning and transfer of learning when ID guidelines are not properly taken into account makes it unlikely that this finding can be attributed to a lack of awareness of the issue [7, 18, 30]. Moreover, evidence of positive learning and transfer outcomes when instructional approaches adhere to evidence-based ID guidelines has been produced for other areas of content besides simulation training of high-risk situations, including evidence-based medicine and decision-making [9, 11, 12, 25].

While adequate use of evidence-based instructional features has been shown to be necessary for ensuring the effectiveness of various methods of instruction, including simulation training, faculty development is another crucial factor contributing to the success of simulation [28, 31, 32]. The use of strategies to enhance awareness among faculty members with regard to incorporating innovative designs has thus been acknowledged to contribute to better simulation outcomes and should be promoted [33]. We, therefore, believe that faculty development could further raise awareness regarding the benefits of adequately using and describing the use of relevant evidence-based ID guidelines for effective simulation training outcomes.

One practical implication of our findings may be to recommend the use of a checklist of ID features based on the items described in our rating scale (Table 1) when designing simulation training. Most likely, some adjustments would have to be made, such as varying the number of cases, in so far as this is feasible, to accommodate budget and time constraints. Such a checklist would also probably require some tailoring before being applied to simulation training formats with specific goals (e.g., mastery of learning). We are aware, however, that such adjustments and tailoring may be particularly challenging, and this may explain the lack of description found.

Our concern with these findings regarding a general tendency not to report adherence to evidence-based ID guidelines is underlined by the results for specific items from the rating scale. For instance, it is worth drawing special attention to items from the authenticity subscale, which specifically refers to exposure to variability with phrases such as “scenarios differ from each other to the same extent as real-life tasks” and “scenarios are sequenced from simple to complex.” Potential lack of exposure to multiple scenarios may seriously jeopardize simulation training for high-risk situations, since this has a detrimental effect on a core complex learning principle for achieving transfer — exposure to broad clinical variation [15, 34]. When managing a complex high-risk situation, such as PPH, healthcare professionals should be able to make use of a systematic approach to problem solving, so as to be able to properly manage the clinical conditions present. Such ability relies heavily on exposure to clinical variation, if it is to be adequately developed [16, 35–37].

The influence on learning and transfer of learning of the various ID elements described in the rating scale items of each of the subscales (authenticity, activation of prior knowledge, demonstration, application, and integration/transfer) has frequently been demonstrated [14, 15, 19, 35, 37]. Therefore, even for a subscale with an IRR considered to be “fair,” such as “demonstration,” possible neglect of some of its instructional features may compromise the effectiveness of simulation training. For instance, failing to demonstrate the skills to be learned, as highlighted in the items “trainees are given demonstrations of the skills and/or models of the behaviors they are expected to learn” and “trainees receive multiple demonstrations that represent alternative ways of performing the skills that need to be learned” may also significantly hinder the complex learning and transfer of learning essential for the proper management of high-risk situations, such as postpartum hemorrhage [15, 19, 38].

Our overall findings provide further support for concerns previously raised by systematic reviews on simulation training effectiveness and the lack of use of evidence-based ID guidelines [7, 18]. We also consider the large number of articles identified and included in our analysis an important strength of our study. It is also worth noting the large number of more recent studies included in our analysis, demonstrating growing interest in training healthcare providers for high-risk situations such as PPH [39]. Our findings, however, indicate that even recent studies of simulation neglect to describe using evidence-based ID guidelines and it is thus reasonable to infer that they did not use them. This may significantly compromise learning and transfer of learning. Furthermore, such studies indicate a worrying lack of awareness regarding these ID guidelines on the part of those who design such simulation training [1, 40].

We acknowledge that some of the articles analyzed did report adherence to evidence-based ID guidelines in the PPH simulation training described. However, our strategy using mean score per subscale for our analysis may have led to some of the instructional strengths of some of the simulation trainings described being overlooked and this should be considered a limitation of our study. Analysis of a single simulation training content area (i.e., PPH) may also be seen as a study limitation, notwithstanding the high epidemiological prevalence of PPH and its similarity to other high-risk situations. However, other training content areas that focus more on deliberate practice of routine aspects of a task (such as Rapid Cycle Deliberate Practice) [37] and less on whole-task practice may require a different set of instructional design guidelines. In terms of the selection of raters, our goal of achieving an adequate number of participants may have led to the inclusion of raters who did not strictly adhere to Ericsson’s [41, 42] criteria for being considered an expert.

Furthermore, some of our experts may have lacked the PPH knowledge necessary to assess content-related design principles (e.g., authenticity). Finally, our aggregation protocol — recoding “not described”/ “not applicable” as “strongly disagree” — may also be seen as a study limitation. We nevertheless believe it is justifiable to consider the lack of reporting of the use of evidence-based ID guidelines as indicating potential disregard as to the importance of such guidelines.

Future studies of the use of evidence-based instructional design guidelines in healthcare simulation should include a larger number of content areas for analysis and aim to identify instructional strengths using specific simulation trainings described in the literature. Likewise, exploratory research design may contribute to a better understanding of the current reasons for shortcomings with regard to adequate description of ID guideline features. The use of alternative rating strategies may also improve interrater reliability.

## Conclusion

In conclusion, we highlight the overall paucity of descriptions of the use of evidence-based ID guidelines in simulation training programs for high-risk situations, such as PPH. Encouraging faculty to further promote adequate use and description of these guidelines, particularly when reporting data regarding simulation training programs, may help to improve simulation training effectiveness and transfer of learning.

## Supplementary Information

**Additional file 1.** This additional file contains the following sequence of instructions received by each rater: 1- Explanatory email, 2- Information Table Subset*^1^, 3- Rating Instrument Subset*^1*2^, 4- Additional Information Table Subset* ^*^1 – the table subset for each article varied according to the subset to which each rater was allocated; *2 each rater received their own rating instrument with entries relating to the corresponding article subsets.

## Data Availability

The datasets used and/or analyzed during the current study are available from the corresponding author on reasonable request.

## Abbreviations

ID: Instructional design
PPH: Postpartum hemorrhage
