# Supplementary material for: The limited use of instructional design guidelines in healthcare simulation scenarios: an expert appraisal
Source: Adv Simul (Lond). 2022 Sep 24;7:30. doi: 10.1186/s41077-022-00228-x (PMC9509554; doi:10.1186/s41077-022-00228-x)
Supplement: Supplementary file 1 — Additional file 1. This additional file contains the following sequence of instructions received by each rater: 1- Explanatory email, 2- Information Table Subset*1, 3- Rating Instrument Subset*1*2, 4- Additional Information Table Subset* *1 – the table subset for each article varied according to the subset to which each rater was allocated; *2 each rater received their own rating instrument with entries relating to the corresponding article subsets. [file 41077_2022_228_MOESM1_ESM.docx]

**Additional File to Review Manuscript ASIM-D-22-00016 Advances in Simulation Submission June2022**

We have pasted below the sets of instructions received by each rater after agreeing on participating in the study. The following five sets of instructions are available:

|  | **Sets of instructions received by the raters** | **Page** |
| --- | --- | --- |
| **1** | **Example of email received by the rater** | 3 |
| **2** | **Information Table Subset - Articles' Tables PPH Simulation & ID Subset1to7**^*1^ |  |
|  | 1. **Subset1to7** | 4 |
|  | 1. **Subset8to14** | 16 |
|  | 1. **Subset15to21** | 34 |
|  | 1. **Subset22to28** | 51 |
|  | 1. **Subset29to32** | 67 |
| **3** | **Rating Instrument Subset (Example of instrument: Rating Instrument PPH Simulation & ID Subset1to7_SORENSEN^*1,*2^** | **75** |
| **4** | **Additional Information Table – ADDITIONAL INFO PPH Simulation & ID Subset1to7.doc**^*1^) |  |
|  | 1. **Subset1to7** | 78 |
|  | 1. **Subset8to14** | 80 |
|  | 1. **Subset15to21** | 84 |
|  | 1. **Subset22to28** | 90 |
|  | 1. **Subset29to32** | 92 |
| **5** | **Each rater received the complete articles of their corresponding subsets: 1to7; 8to14; 15to21; 22to28; 29to32. Please see Table 4 in the manuscript with the complete references for all 32 articles.** | 94 |
|  | *1 – each article’s table subset varied according to the subset each rater was allocated to; *2 each rater received their own rating instrument adjusted to the subset he/she was allocated to. | |

Below, we provide two tables. In **Table 1** is a list of articles divided in subsets, in **Table 2** The distribution of raters per subsets.

**Table 1.** List of articles divided in subsets

| **LISTA DOS ARTIGOS** | | |
| --- | --- | --- |
| **Subset of Articles 1-7** | **Subset of Articles 8-14** | **Subset of Articles 15-21** |
| **Paper # 1 Andriguetti** | **Paper # 8 Egenberg** | **Paper # 15 Nelissen** |
| **Paper # 2 Birch** | **Paper # 9 Fialkow** | **Paper # 16 Philippi** |
| **Paper # 3 Chichester** | **Paper # 10 Magee** | **Paper # 17 Roberstson** |
| **Paper # 4 Clark** | **Paper # 11 Markova** | **Paper # 18 Crofts** |
| **Paper # 5 Cooper** | **Paper # 12 Marshal** | **Paper # 19 Siassakos** |
| **Paper # 6 Scholes** | **Paper # 13 Maslovitz 2007** | **Paper # 20 Straub** |
| **Paper # 7 Deering** | **Paper # 14 Maslovitz 2008** | **Paper # 21 Vadnais** |

**Table 2** Distribution of raters per subsets

| **Raters’ distribution per Subsets** | | | | | | | | | |
| --- | --- | --- | --- | --- | --- | --- | --- | --- | --- |
| **Subset of Articles 1-7** | | **Subset of Articles 8-14** | | **Subset of Articles 15-21** | | **Subset of Articles 22-28** | | **Subset of Articles 29-32** | |
| **Rater #1** | Bezerra_Patrícia | **Rater #9** | Carvalho_Filho_Marco | **Rater #17** | Andriguetti_Tia | **Rater #24** | Asada_ Yoshikazu | **Rater #31** | Aleluia_Ieda |
| Rater #2 | Egenberg | **Rater #10** | Cury_Patricia | **Rater #18** | Attoe_Cris | **Rater #25** | Georg_Carina | **Rater #32** | Andersen_Steven |
| **Rater #3** | Hewett_Angela | **Rater #11** | Endacott | **Rater #19** | Boyer_Louis | **Rater #26** | Gronier_Marleen | **Rater #33** | Appoloni_Thiago |
| **Rater #4** | Nilsson_Cecilia | **Rater #12** | Peters_Sanne | **Rater #20** | Chichester | **Rater #27** | Knobel_Roxana | **Rater #34** | Cheng_Jeffrey |
| **Rater #5** | Rebeca_Tereza | **Rater #13** | Ribeiro_Ligia | **Rater #21** | Cooper | **Rater #28** | Lustosa_Kristopherson | **Rater #35** | Costa_Marcia |
| **Rater #6** | Rossi_Elaine | **Rater #14** | Roosmalen | **Rater #22** | Kinsman | **Rater #29** | Polido_Carla | **Rater #36** | Grangeia_Tiago |
| **Rater #7** | Sorensen | **Rater #15** | Santos_Elizabeth | **Rater #23** | Maslovitz | **Rater #30** | Thingaard_Ebbe | **Rater #37** | Grierson_Lawrence |
| **Rater #8** | Souza_Edvaldo | **Rater #16** | Straub_Heather |  |  |  |  | **Rater #38** | Ong_Caroline |
|  |  |  |  |  |  |  |  | **Rater #39** | Thomsen_AnnSofia |
|  |  |  |  |  |  |  |  | **Rater #40** | Todsen_Tobias |

1. **EMAIL RECEIVED BY the raters RATER** (example email for the rater SORENSEN who assessed the subset of articles1to7)

Dear Rater SORENSEN,

Thank you for accepting to collaborate with the study on PPH Simulation & Instructional Design.

Please find attached the following two Word**^®^** documents: "**Articles' Tables PPH Simulation & ID Subset1to7**” and **"Rating Instrument PPH Simulation & ID Subset1to7_SORENSEN"**.

In the first, "**Articles' Tables PPH Simulation & ID Subset1to7**", you will find tables where I have listed the texts extracted from the articles at which the simulation based training programs were described. After some introductory information about the article (such as title, authors, journal and abstract), the main information collected from the text is disposed into rows corresponding to categories such as presentation, practice, feedback and assessment. For each one of these categories, there is an additional subdivision (What?, How?, Medium?) related to, for example: What was the presented information?"; How was the practice executed?"; or Which medium was used to give learners feedback?. (A few empty cells may appear if the corresponding information was not available in the original text.)

As for the Rating Instrument document, in there you will find the rating scale per se. I suggest that you start by opening this word file and reading through the items, so as to become familiar with the items to be evaluated. The rating scale was developed with basis on Merrill's First Principles of Instruction. A 5 point Likert scale was adopted and I ask you to please type your answer in the corresponding cell. In the hypothesis that you find that such item was not present in the "**Articles' Tables PPH Simulation & ID Subset1to7**" document tables, please type ND= not described.

You will only need to work with these first two word documents, but in case you wish to check the original articles from which the simulation based training programs were extracted, they are also following attached. The third word document, which also follows attached, has additional information such as: study aim, study design and number of participants and their background. Once again, this information is not necessary for your analysis, but they were made available if you may wish to consult them. (**ADDITIONAL INFO PPH Simulation & ID Subset1to7.doc**)

Please let me know if you need any further information.

My original expectation is to receive your answers in about 3 or 4 weeks. In case you believe that this deadline does not seem feasible to you, please let me know when you expect to be able to send me your evaluation.

I thank you deeply for your generous help.

Best regards,

Brena Melo

1. **Information Table Subset - Articles' Tables PPH Simulation & ID Subset**
   1. **Articles' Tables PPH Simulation & ID Subset1to7**

| **Article # 1 Andriguetti** | | |
| --- | --- | --- |
| **Title** | | Shoulder dystocia and postpartum hemorrhage simulations: student confidence in managing these complications. |
| **Authors** | | [Andrighetti TP](http://www.ncbi.nlm.nih.gov/pubmed/?term=Andrighetti%20TP%5BAuthor%5D&cauthor=true&cauthor_uid=22251913), [Knestrick JM](http://www.ncbi.nlm.nih.gov/pubmed/?term=Knestrick%20JM%5BAuthor%5D&cauthor=true&cauthor_uid=22251913), [Marowitz A](http://www.ncbi.nlm.nih.gov/pubmed/?term=Marowitz%20A%5BAuthor%5D&cauthor=true&cauthor_uid=22251913), [Martin C](http://www.ncbi.nlm.nih.gov/pubmed/?term=Martin%20C%5BAuthor%5D&cauthor=true&cauthor_uid=22251913), [Engstrom JL](http://www.ncbi.nlm.nih.gov/pubmed/?term=Engstrom%20JL%5BAuthor%5D&cauthor=true&cauthor_uid=22251913). |
| **Journal & Publishing information** | | [J Midwifery Womens Health.](http://www.ncbi.nlm.nih.gov/pubmed/22251913) 2012 Jan-Feb;57(1):55-60. doi: 10.1111/j.1542-2011.2011.00085.x. Epub 2011 Sep 23. |
| **Abstract** | | **Introduction:** Simulation is an effective teaching strategy for educating health professionals. However, little is known about the effectiveness of simulations in midwifery education. The purpose of this project was to determine whether the use of high-fidelity simulations for the obstetric emergencies of shoulder dystocia and postpartum hemorrhage increases student confidence in managing these complications.  **Methods:** The participants were registered nurses enrolled in a graduate midwifery education program in the Southeastern United States. Student confidence in learning to manage shoulder dystocia and postpartum hemorrhage was studied in 2 groups of students. The control group (n = 10) received standard teaching methods consisting of discussion, watching a video, and low-fidelity teaching methods. The intervention group (n = 18) received a high-fidelity simulation learning experience. Student confidence was measured before and after each learning experience using a validated, 8-item, Likert-type scale.  **Results:** In the control group, student confidence did not significantly increase after a classroom discussion or low-fidelity simulation experience. Student confidence increased significantly (*P* < .01) after the high-fidelity simulation learning experiences. When the differences between the pretest and posttest scores were compared for the control and intervention groups, there was a moderate effect size (0.54) for the intervention group for the shoulder dystocia simulations and a large effect size (1.68) for the postpartum hemorrhage simulations.  **Conclusion:** High-fidelity simulations for shoulder dystocia and postpartum hemorrhage significantly increased student confidence in managing these complications. |
| **Presentation**  **(**Information presented to learners) | What? The content of the presentation / practice scenarios / feedback ... | Student confidence in learning to manage shoulder dystocia and postpartum hemorrhage was studied in 2 groups of students. |
|  | How? The form of the presentation / practice scenarios / feedback ... (e.g., sequencing, scaffolds etc.) | The control group (n = 10) received standard teaching methods consisting of discussion, watching a video, and low-fidelity teaching methods. The intervention group (n = 18) received a high-fidelity simulation learning experience. The control group underwent training about the management of PPH using a discussion format that used a question and answer approach. *pg56* |
|  | Medium? The medium used (on paper, lecture, group discussion, simulation etc.) |  |
| **Practice**  **(**Practice provided to learners) | What? The content of the presentation / practice scenarios / feedback ... | .. high-fidelity shoulder dystocia and PPH simulations... |
|  | How? The form of the presentation / practice scenarios / feedback ... (e.g., sequencing, scaffolds etc.) | For the intervention group, high-fidelity shoulder dystocia and PPH simulations were developed using principles of adult learning and novice to expert (A. Clardy, unpublished manuscript, 2005). *pg56* |
|  | Medium? The medium used (on paper, lecture, group discussion, simulation etc.) | Video and demonstration by faculty. The simulation environment recreated the appearance and feeling of a labor and delivery unit, with visual and auditory cues to simulate a real-life encounter. The equipment included a static mannequin, because a high- fidelity simulator was not able to portray a shoulder dystocia or PPH in a more realistic fashion. *pg56* Psychological fidelity was mimicked by having students role play 1 of 3 personnel (student nurse-midwife, nurse, significant other) involved in the room during the complication. Each student played the role of the student nurse-midwife and needed to make decisions in that role in consultation with their preceptor. Faculty played the role of preceptor. *pg57* |
| **Feedback**  **(**Feedback given to learners) | What? The content of the presentation / practice scenarios / feedback ... | After each simulation experience, students participated in a group debriefing session in which students commented on the strengths and limitations of the simulation session and their areas of needed improvement and reflected on their feeling during and after simulation. *pg57* |
|  | How? The form of the presentation / practice scenarios / feedback ... (e.g., sequencing, scaffolds etc.) |  |
|  | Medium? The medium used (on paper, lecture, group discussion, simulation etc.) |  |
| **Assessment**  **(**Assessing the learners) | What? The content of the presentation / practice scenarios / feedback ... | ... student confidence in managing the obstetric emergencies of shoulder dystocia and PPH. |
|  | How? The form of the presentation / practice scenarios / feedback ... (e.g., sequencing, scaffolds etc.) | Student confidence was measured immediately before and after the learning experiences. All students completed the Student Satisfaction and Self Confidence in Learning instrument immediatelly before and after their learning experience. *pg56* Student confidence was measured using the National League for Nursing Student Satisfaction and Self Confidence in Learning instrument. This instrument was developed to evaluate student confidence after undergoing a simulation learning experience and has been used in other studies measuring student confidence with high-fidelity simulations. *pg56* |
|  | Medium? The medium used (on paper, lecture, group discussion, simulation etc.) | Student confidence was measured before and after each learning experience using a validated, 8-item, Likert-type scale. Eight of the items on this instrument measured student confidence and were used for this study to create a confidence score. The items used a Likert-type scale with responses ranging from 1 (strongly disagree) to 5 (strongly agree). One item was negatively worded, so the scoring of that item was reversed. The individual scores on the 8 items were summed to derive a total confidence score. *pg56* The content validity of this instrument was previously established by consulting 9 clinical experts in nursing. Internal consistency of the items on the instrument was previously established with a Cronbach’s alpha of .87.The Cronbach’s alpha for the 8 items used in this study was .80. *pg56* |
|  | | |
| **Article # 2 Birch** | | |
| **Title** | | Obstetric skills drills: Evaluation of teaching methods |
| **Authors** | | L. Birch, N. Jones, P.M. Doyle, P. Green, A. McLaughlin, C. Champney, D. Williams, K. Gibbon, K. Taylor |
| **Journal & Publishing information** | | Nurse Education Today (2007) 27, 915–922 |
| **Abstract** | | **Objective:** To determine the most effective method of delivering training to staff on the management of an obstetric emergency. Subjects: The research was conducted in a District General Hospital in the UK, delivering approximately 3500 women per year. Thirty-six staff, comprising of junior and senior medical and midwifery staff were included as research subjects. Each of the staff members were put into one of six multi-professional teams. Effectively, this gave six teams, each comprising of six members.  **Method:** Three teaching methods were employed. Lecture based teaching (LBT), simulation based teaching (SBT) or a combination of these two (LAS). Each team of staff were randomly allocated to undertake a full day of training in the management of Post Partum Haemorrhage utilising one of these three teaching methods. Team knowledge and performance were assessed pre-training, post training and at three months later. In addition to this assessment of knowledge and performance, qualitative semi-structured interviews were carried out with 50% of the original cohort one year after the training, to explore anxiety, confidence, commu- nication, knowledge retention, enjoyment and transferable skills.  **Results:** All teams improved in their performance and knowledge. The teams taught using simulation only (SBT) were the only group to demonstrate sustained improvement in clinical management of the case, confidence, communication skills and knowledge. However, the study did not have enough power to reach statistical significance. The SBT group reported transferable skills and less anxiety in subsequent emergencies. SBT and LAS reported improved multidisciplinary communication. Although tiring, the SBT was enjoyed the most.  **Conclusion:** Obstetrics is a high-risk speciality, in which emergencies are to some extent, inevitable. Training staff to manage these emergencies is a fundamental principal of risk management. Traditional risk management strategies based on incident reporting and event analysis are reactive and not always effective. Simulation based training is an appropriate proactive approach to reducing errors and risk in obstetrics, improving teamwork and communication, whilst giving the student a multiplicity of transferable skills to improve their performance. |
| **Presentation**  **(**Information presented to learners) | What? The content of the presentation / practice scenarios / feedback ... | The following points influenced the decision to opt for post partum haemorrhage (PPH):   - The performance of the team was to be assessed, not individual skill. As such, an emergency scenario where several members of a team were actively involved in treatment was needed. - The scenario needed to be realistic with an opportunity for individual as well as team learning... PPH is the most common recorded emergency in the hospital where the research took place.   *pg 918/919*   In order to assess prior knowledge, at the start of the training day each team completed a questionnaire on PPH.   Test papers were completed anonymously. Participants were   required to define PPH, give answers relating to causes and predisposing factors and to complete a flow chart of the stages involved in managing a primary PPH. *pg 919* The same topics were covered in all sessions irrespective of teaching format, as such the content of the training did not change, merely the manner in which that content was delivered. Team roles, leadership, communication and delegation were key topics alongside physiology and biochemistry. The method of delivery was the key variable.  *pg 920* |
|  | How? The form of the presentation / practice scenarios / feedback ... (e.g., sequencing, scaffolds etc.) | Following the initial assessment, each team underwent a full day of training. The format of training was randomly allocated to each group and incorporated theory and/or practical elements. Two of the teams were allocated to receive traditional lecture based teaching (LBT). Two were allocated to receive a half-day of lectures followed by a half day of simulation training (LAS). The final two teams received a full day of simulation-based training but no theoretical component (SBT).  *pg 920* The simulator was placed in the labor suite in the hospital where the study was conducted. This ensured the surroundings provided as realistic an environment as is possible for the simulator to reside. *pg 918* The simulation based training (SBT) involved repeating the emergency scenario under the guidance of a senior midwife and consultant obstetrician. Teams were stopped and questioned on their management at various stages and alternative approaches discussed. The lecture based training (LBT) involved formal tutorial and informal discussion in a classroom environment. The lecture and simulation training (LAS) had classroom-based tutorials in the morning and simulation training in the afternoon.   *pg 920* |
|  | Medium? The medium used (on paper, lecture, group discussion, simulation etc.) |  |
| **Practice**  **(**Practice provided to learners) | What? The content of the presentation / practice scenarios / feedback ... |  |
|  | How? The form of the presentation / practice scenarios / feedback ... (e.g., sequencing, scaffolds etc.) | Multi-disciplinary teams were randomly selected from obstetric and midwifery staff to participate in one day of training on an obstetric emergency topic. *pg 918* Teams were not informed in advance, what the emergency scenario would be. ... The ‘‘patient’’, a 28 year old primigravida, with no significant medical or obstetric history, had just had a spontaneous vaginal delivery of a full term infant. The perineum was intact, but there was a small vaginal wall laceration that had not been sutured. Blood loss at delivery was recorded as 300 ml. Mock case notes with full booking history, antenatal care, hemoglobin results, blood group and intrapartum record were available in the room. The locum midwife handed over care of the patient to one of the junior midwives in the experimental team. Over the next five minutes, vaginal blood loss was gradually increased. The junior midwife was free to call on other members of her team as she felt necessary. Physiological readings such as blood pressure and pulse were titrated according to the treatment given and total blood loss.   *pg 919* |
|  | Medium? The medium used (on paper, lecture, group discussion, simulation etc.) | The simulator that was used to replicate the patient for this research was a Laerdal SimMan^TM^, which is a mannequin animated with a variety of electromechanical or pneumatic devices that produce respiratory movement, palpable pulses, heart and lung sounds and realistic airway anatomy. *pg 917* The AHPS need to be programmed to act/re-act to external stimuli and will therefore allow for realistic advancement within the scenario enabling the student to experience experiential learning. *pg 917* The ability of the manikin to elicit realistic responses to medical interventions in real time allows the Obstetric team to work together without hesitation, for it is the use of the full body manikin placed within a real life environment that can influence the development of student attitudes, psychomotor skills, knowledge and clinical problem solving abilities. *pg 917/918* Each team was then taken to delivery suite and asked to deal with a simulated post partum hemorrhage, as outlined above. This was video recorded. *pg 919* |
| **Feedback**  **(**Feedback given to learners) | What? The content of the presentation / practice scenarios / feedback ... | At the end of each training day, a ‘‘debriefing’’ session was held were all team members could feedback and reflect on the training they had received. *pg 919* |
|  | How? The form of the presentation / practice scenarios / feedback ... (e.g., sequencing, scaffolds etc.) | At the end of the days training, the initial assessment components of questionnaire and video OSCE were repeated. This gave the post training score. In addition, participants were asked to indicate on a five-point scale, their perception of their knowledge, confidence and effectiveness as a team. Also, how much they had enjoyed the training. In order to compare long-term knowledge retention, three months later, the same teams were assembled again and the questionnaire and video OSCE repeated once more. *pg 919* Each team received a score from each of the independent assessors derived from the video and questionnaires for each of the three assessment periods: (1) Before training (pre training score), (2) at the end of the days training (post-training  score), (3) three months after training (three month score). *pg 920* |
|  | Medium? The medium used (on paper, lecture, group discussion, simulation etc.) |  |
| **Assessment**  **(**Assessing the learners) | What? The content of the presentation / practice scenarios / feedback ... | As a result of comments received during the debrief sessions, this research was supplemented by further qualitative work (phase two). Phase two involved the completion of semi-structured interviews to explore the topics identified during the debrief sessions held after phase one.  *...* Key themes for the interviews included individual anxiety when faced with an emergency, communication between professionals, transferable skills and individual confidence, enjoyment of the training method undertaken and knowledge retention. *pg 920* |
|  | How? The form of the presentation / practice scenarios / feedback ... (e.g., sequencing, scaffolds etc.) | The recording was later analysed by two independent experienced obstetric staff, each of whom independently assessed team performance using pre-set 89 Objective Structured Clinical Examination (OSCE) criteria. *pg 919* Phase two involved the completion of semi-structured interviews to explore the topics identified during the debrief sessions held after phase one. These interviews were held approximately one year after the completion of the training. In addition to this assessment of knowledge and performance, qualitative semi-structured interviews were carried out with 50% of the original cohort one year after the training, to explore anxiety, confidence, communication, knowledge retention, enjoyment and transferable skills. The(se) interviews were semi-structured and tape recorded. *pg 920* |
|  | Medium? The medium used (on paper, lecture, group discussion, simulation etc.) |  |
|  | | |
| **Article # 3 Chichester** | | |
| **Title** | | A cost-effective approach to simulation-based team training in obstetrics. |
| **Authors** | | [Chichester M](http://www.ncbi.nlm.nih.gov/pubmed/?term=Chichester%20M%5BAuthor%5D&cauthor=true&cauthor_uid=25495970), [Hall NJ](http://www.ncbi.nlm.nih.gov/pubmed/?term=Hall%20NJ%5BAuthor%5D&cauthor=true&cauthor_uid=25495970), [Wyatt TL](http://www.ncbi.nlm.nih.gov/pubmed/?term=Wyatt%20TL%5BAuthor%5D&cauthor=true&cauthor_uid=25495970), [Pomilla R](http://www.ncbi.nlm.nih.gov/pubmed/?term=Pomilla%20R%5BAuthor%5D&cauthor=true&cauthor_uid=25495970). |
| **Journal & Publishing information** | | [Nurs Womens Health.](http://www.ncbi.nlm.nih.gov/pubmed/25495970) 2014 Dec;18(6):500-7. doi: 10.1111/1751-486X.12162. |
| **Abstract** | | **Abstract:** Many larger facilities regularly stage obstetric drills in modern simulation departments equipped with expensive simulators. Despite lacking these resources, we wanted to provide effective simulation training at our rural hospital. A team of clinicians and educators developed a cost-effective and time-efficient simulation drill for nurses, which included both a didactic review and a simulation day. The drill included obstetric providers and incorporated scenarios for shoulder dystocia, neonatal resuscitation and postpartum hemorrhage. This was a successful multidisciplinary learning experience that was high in creativity and teamwork, but low in cost. |
| **Presentation**  **(**Information presented to learners) | What? The content of the presentation / practice scenarios / feedback ... | The drill included obstetric providers and incorporated scenarios for shoulder dystocia, neonatal resuscitation and postpartum hemorrhage. *pg 501* To increase interprofessional collaboration, the nurse manager invited obstetric providers to participate in the drill. ... As the scenario evolved, the unit’s neonatal nurse practitioner offered to include a neonatal resuscitation drill. To maximize the time available, we decided to incorporate all three scenarios stacked into one simulation, beginning with shoulder dystocia, leading to a neonatal resuscitation and concluding with postpartum hemorrhage. This design allowed for the combination of multiple learning scenarios that more closely resembled realistic circumstances. These particular scenarios were chosen as the most common obstetric emergencies warranting regular drills to prepare obstetric nurses and providers (Alderman, 2012). *pg 502* |
|  | How? The form of the presentation / practice scenarios / feedback ... (e.g., sequencing, scaffolds etc.) | Plans were made to hold both a didactic review and a simulation day similar to what the students had experienced. The didactic portion, led by the L&D nurse, was an interactive, evidence-based lecture with case presentations integrated throughout. With the knowledge that a prescenario learner activity is a critical component of scenario development, the lecture portion was offered during a monthly staff meeting to facilitate staff attendance (Waxman, 2010). The nurse manager chose to make the simulation drills mandatory/paid time, and a sign-up sheet was posted with slots for four to five nurses per 45-minute time slot. *pg 502* Most groups completed the drills and debriefing in less than the 45-minute allotted time. *pg 504* |
|  | Medium? The medium used (on paper, lecture, group discussion, simulation etc.) | The team developed objectives targeted at staff nurses’ learning needs that could be met either during the scenario or the debriefing session. *pg 503* |
| **Practice**  **(**Practice provided to learners) | What? The content of the presentation / practice scenarios / feedback ... |  |
|  | How? The form of the presentation / practice scenarios / feedback ... (e.g., sequencing, scaffolds etc.) | The educator from the school of nursing who had voiced the woman for the student drills would again assume this role. She would be strategically positioned behind a curtain at the head of the bed where her questions, comments and, at times, hysterics could afford a higher level of realism to the low-fidelity simulator. ... The planners agreed to share the task of mixing enough simulated blood for the hemorrhage scene, where an enema bag would be filled with varying amounts for each group. The enema bag tubing would be placed beneath the simulator to hemorrhage on command when the flow clamp was released. ... The team developed objectives targeted at staff nurses’ learning needs that could be met either during the scenario or life the debriefing session. The expert nurse would serve as facilitator by providing guidance during the simulation via narration and leading the debriefing session. *pg 503* |
|  | Medium? The medium used (on paper, lecture, group discussion, simulation etc.) | Much thought was given to finding a location that would optimize realism and influence the learners’ ability to suspend disbelief. Given the unpredictability of the L&D’s unit census, we planned to hold the simulation in a triage room that’s equipped to serve as an overflow labor room. The standard monitors and equipment it routinely houses made it a realistic alternative setting. An adult female simulator, Mrs. Chase, a curriculum staple for decades, would be brought from the school of nursing along with miscellaneous supplies. *pg 503* |
| **Feedback**  **(**Feedback given to learners) | What? The content of the presentation / practice scenarios / feedback ... | Debriefing always had two standard questions: *What do you think went well?* And *What do you think could have been done better?* Further discussion and learning were spurred by asking additional review questions, such as *what risk factors/indicators were noted for a shoulder dystocia or postpartum hemorrhage? pg 505* |
|  | How? The form of the presentation / practice scenarios / feedback ... (e.g., sequencing, scaffolds etc.) | Feedback was obtained and learning assessed through a posttest, written evaluations and a debriefing session. *pg 504* |
|  | Medium? The medium used (on paper, lecture, group discussion, simulation etc.) |  |
| **Assessment**  **(**Assessing the learners) | What? The content of the presentation / practice scenarios / feedback ... | To reinforce material from the didactic, a 25-question quiz was available to be done individually or as a group, with the final exam being a table with three pads full of simulated blood. The challenge would be given to see who could most accurately estimate the volume in each pad, with a prize awarded for the closest estimate. *pg 504* Our feedback on the learning activity was positive, but for it to be considered truly successful, outcome data should also in- clude staff nurses’ feedback following a real-life emergency and how the simulation experience influenced team performance during an actual event. *pg 506* |
|  | How? The form of the presentation / practice scenarios / feedback ... (e.g., sequencing, scaffolds etc.) | We gathered data 1 year later to do just that. The survey asked nurses if they had encountered any ofthe three emergencies in practice, and if the team simulation had improved their ability to act. While only 25 percent of the nurses subsequently had an emergency situation in the year following the team drill, of those who had, 100 percent reported feeling better prepared for it as a result of the simulation. *pg 506* |
|  | Medium? The medium used (on paper, lecture, group discussion, simulation etc.) |  |
|  | | |
| **Article # 4 Clark** | | |
| **Title** | | Team Training/ Simulation |
| **Authors** | | Erin A. S. Clark, Janet Fisher, Julia Arafeh, Maurice Druzin |
| **Journal & Publishing information** | | Clinical Obstetrics and Gynecology Volume 53, Number 1, 265–277 r 2010, |
| **Abstract** | | **Abstract:** Obstetrical emergencies require the rapid formation of a team with clear communication, strong leadership, and appropriate decision-making to en- sure a positive patient outcome. Obstetric teams can improve their emergency response capability and efficiency through team and simulation training. Postpartum hemorrhage is an ideal model for team and simulation training, as postpartum hemorrhage requires a multidisciplinary team with the capability to produce a protocol-driven, rapid response. This article provides an overview of team and simulation training and focuses on applications within obstetrics, particularly preparation for postpartum hemorrhage. **Key words:** postpartum hemorrhage, team training, simulation |
| **Presentation**  **(**Information presented to learners) | What? The content of the presentation / practice scenarios / feedback ... | DEVELOPING LEARNING OBJECTIVES In determining the learning objectives for team training in PPH, the L&D Education Committee, in collaboration with the Department of Obstetrics and Gynecology, focused on gaps in our staff knowledge and attitudes. We were guided by the findings of JCAHO’s Sentinel Alert #30, Preventing infant death and injury during delivery and the 2006 JCAHO designated National Patient Goals, including ‘‘improving the effectiveness of communication among caregivers’’ and ‘‘implementing a standardized approach to ‘hand off’ communications.’’ Although we felt we had a high functioning and collegial team, we recognized that barriers to effective communication and teamwork were the root cause in several of our own ‘‘sentinel events.’’ Improvement of communication skills was therefore our number one team-training objective. *pg 266* Specifically delegating tasks was another behavioral objective. Staff members were asked to say a specific person’s name, point at a person, or make direct eye contact and say ‘‘you’’ and then state the job needing to be performed. Staff were given the following example of more effective communication and team functioning: Say ‘‘Janet, you call the lab to crossmatch 4 units of blood,’’ instead of saying to the room, ‘‘crossmatch 4 units of blood.’’ In the latter example, either many people will respond to that order, or no one will. An important objective for improved team functioning was clear identification of the team leader. ... Our final objective was in response to JCAHO’s National Patient Safety Goal #3, ‘‘Improve the safety of using medica- tions.’’ In the development of this objective, we focused on reinforcing the staff knowledge of uterotonic medications used in PPH and discussed standardized use of first-line medications. *pg 272* |
|  | How? The form of the presentation / practice scenarios / feedback ... (e.g., sequencing, scaffolds etc.) | PREPARING THE STAFF TO MEET THE OBJECTIVES  Journal articles and information specific to the learning objectives were distributed to the participants 2 weeks before their scheduled session. To create a ‘‘safe’’ learning environment, we initiated our team-training session with a team meeting where the objectives of the learning exercise were reviewed and the specific behaviors that support these objectives were discussed. Team cohesiveness was further encouraged when the group broke into 2 squads and played PPH Jeopardy using questions focused on the use of uterotonic medications. *pg 272* Finally, the group was brought into the simulation room and given a through orientation to the simulation including how they would be introduced to the clinical situation and how they would receive ongoing data on the simulated patient’s status. *pg 273* |
|  | Medium? The medium used (on paper, lecture, group discussion, simulation etc.) |  |
| **Practice**  **(**Practice provided to learners) | What? The content of the presentation / practice scenarios / feedback ... | Effective learning can occur with very basic equipment and supplies (low fidelity) to very sophisticated simulators (high fidelity). Creating simulations for successful team training exercises is more reliant on creativity and commitment than on having fancy mannequins and expensive audiovisual equipment. Suggested components for successful low-fidelity PPH simulation are listed in Table 1. The goal is always to create an atmosphere where the trainees can achieve a ‘‘suspension of disbelief’’ to allow themselves to fully participate in the exercise. *pg 273* Ordering and obtaining blood products was an important process that was emphasized during this training. During the series of simulations, we discovered some process problems which created a great opportunity to reinforce to the staff the correct steps for ordering and obtaining blood products. Arrangements also were made with the blood bank to have 2 units of ‘‘training blood’’ available for the simulation. *pg 274/275* |
|  | How? The form of the presentation / practice scenarios / feedback ... (e.g., sequencing, scaffolds etc.) | We purchased the basic Noelle Obstetric simulator. Most of the additional resources that we needed were readily available in the hospital. We collected expired saline bags, intravenous lines, and other supplies to add realism to the scenario. The Biomedical Department had simulators for testing medical equipment and they allowed us to borrow these for our simulation and team training exercises. A brief in-service on how to run the blood pressure/pulse and electrocardiogram simulators from one of the biomedical engineers allowed us to generate vital signs on equipment that the staff normally uses. The participants in the scenario looked at these monitors to give them clues on Noelle’s changing clinical condition. When a simulator was not available, for example the pulse oximeter, we simply intermittently taped a sign on the vital sign monitor to alert the team as to how this assessment varied during the scenario. *pg 273* It took 3 facilitators to run our simulation: one to manage the vital signs monitors, one to be the voice of Noelle and manage the blood flow, and one to film. This method of teaching is unquestionably more labor intensive, both in personnel and time, than didactic methods. In addition, simulation and team-training in the work place, rather than at a ‘‘simulation center,’’ poses unique challenges and advantages (Fig. 3). The advantages far outweigh the negatives at our institution. Staff members are able to participate during their regularly scheduled shifts, logistical lessons are concrete and easily extrapolated to everyday patient care, and our relatively low-fidelity simulation equipment translates into a successful high-fidelity experience. *pg 275* |
|  | Medium? The medium used (on paper, lecture, group discussion, simulation etc.) | Our Obstetric simulator is a very basic model and was modified to lend additional realism to our simulation. We used large, heavy duty latex balloons found at most party stores for Noelle’s uterus. To create blood clots, the balloon was filled with 4 cups of dark red gelatin and allowed to set in the refrigerator. The balloon was then set out at room temperature for about 4 hours and the balloon was aggressively massaged to break the gelatin up into smaller clot-sized pieces; this pro- cess softened the gelatin enough so that when the ‘‘uterus’’ was creded the ‘‘clots’’ were easily expelled (Fig. 1). To simulate vaginal bleeding, we added powdered blood (available through the simulator manufacturer or other sources and less staining than red food coloring or dye) to a bag of saline and fed the IV tubing through Noelle’s vagina. The facilitator who was the ‘‘voice’’ of Noelle would sit beside the bed, the saline bag on her lap beneath a sheet, and she would squeeze the IV bag to increase the blood flow when indicated (Fig. 2). This system had the added advantage of being able to compare actual ‘‘blood loss’’ to clinician estimates. Our simulation takes place in an actual labor and delivery room. ... To simulate the medications, we fill syringes with water and label the syringes with the appropriate drug and dose. All of the equipment, supplies, and medication are put in the actual places that they are stored on the unit. This adds value to the simulation by emphasizing how simple tasks, such as retrieving medication or supplies, can have a tremendous impact on time and staff resources. A chart was created for ‘‘Noelle Simulation’’ that included a prenatal record, our standard history and physical form, and staff notes describing her labor progress. Nursing documentation is carried out by computer at our hospital and we added Noelle into our system for each simulation. The admitting department created an armband and stamper plate for Noelle, so that we could simulate the exact process that we use for sending laboratories or administering medications. We also alerted all hospital departments whose services could be called upon during the simulation (specifically, anesthesia and pediatrics). These departments have responded favorably to our efforts and generally send appropriate staff to respond to our simulated emergency. *pg 274* |
| **Feedback**  **(**Feedback given to learners) | What? The content of the presentation / practice scenarios / feedback ... |  |
|  | How? The form of the presentation / practice scenarios / feedback ... (e.g., sequencing, scaffolds etc.) | We used a 1-hour debriefing session at the conclusion of the exercise to review where errors occurred and how they could potentially have been prevented and to also identify areas of appropriate performance. All sessions were videotaped and then reviewed and analyzed by the participants while guided by an experienced nurse facilitator. The challenge for the facilitator is to create an atmosphere where participants in the simulation feel safe and are willing to engage in a constructive critique of their performance. We start each debriefing session with a discussion of the ground rules of confidentiality and trust. The facilitator’s role in the debriefing is to keep the discussion focused and to use the learning objectives to direct the evaluation. These sessions provide a rich discussion, not only for the stated goals of the training, but also to highlight topics such as effective use of all team members in an emergency and effective leadership behavior. Our experience with videotaping the scenarios has taught us 2 lessons: (1) participants are anxious about being taped and (2) participants recognize that reviewing the tape is the single most powerful teaching tool during the exercise. We reduced this anxiety by acknowledging these feelings upfront, keeping the mood light, and allowing the staff to joke about the experience before starting the debriefing. *pg 275* |
|  | Medium? The medium used (on paper, lecture, group discussion, simulation etc.) |  |
| **Assessment**  **(**Assessing the learners) | What? The content of the presentation / practice scenarios / feedback ... | Evaluating the results of team training is in its infancy and most data are based on attitudes and perceptions of those who have participated in training sessions. However, the positive feedback on the impact of this education is compelling, with many staff stating that they see an improvement in team functioning and communication as a result of the training. Literature supports that participants in simulation and team training have a favorable experience and the perception of the majority is that these methods have a direct impact on improving the quality of patient care. *pg 276* |
|  | How? The form of the presentation / practice scenarios / feedback ... (e.g., sequencing, scaffolds etc.) |  |
|  | Medium? The medium used (on paper, lecture, group discussion, simulation etc.) |  |
|  | | |
| **Article # 5 Cooper** | | |
| **Title** | | Managing women with acute physiological deterioration: student midwives performance in a simulated setting. |
| **Authors** | | [Cooper S](http://www.ncbi.nlm.nih.gov/pubmed/?term=Cooper%20S%5BAuthor%5D&cauthor=true&cauthor_uid=21940231)^1^, [Bulle B](http://www.ncbi.nlm.nih.gov/pubmed/?term=Bulle%20B%5BAuthor%5D&cauthor=true&cauthor_uid=21940231), [Biro MA](http://www.ncbi.nlm.nih.gov/pubmed/?term=Biro%20MA%5BAuthor%5D&cauthor=true&cauthor_uid=21940231), [Jones J](http://www.ncbi.nlm.nih.gov/pubmed/?term=Jones%20J%5BAuthor%5D&cauthor=true&cauthor_uid=21940231), [Miles M](http://www.ncbi.nlm.nih.gov/pubmed/?term=Miles%20M%5BAuthor%5D&cauthor=true&cauthor_uid=21940231), [Gilmour C](http://www.ncbi.nlm.nih.gov/pubmed/?term=Gilmour%20C%5BAuthor%5D&cauthor=true&cauthor_uid=21940231), [Buykx P](http://www.ncbi.nlm.nih.gov/pubmed/?term=Buykx%20P%5BAuthor%5D&cauthor=true&cauthor_uid=21940231), [Boland R](http://www.ncbi.nlm.nih.gov/pubmed/?term=Boland%20R%5BAuthor%5D&cauthor=true&cauthor_uid=21940231), [Kinsman L](http://www.ncbi.nlm.nih.gov/pubmed/?term=Kinsman%20L%5BAuthor%5D&cauthor=true&cauthor_uid=21940231), [Scholes J](http://www.ncbi.nlm.nih.gov/pubmed/?term=Scholes%20J%5BAuthor%5D&cauthor=true&cauthor_uid=21940231), [Endacott R](http://www.ncbi.nlm.nih.gov/pubmed/?term=Endacott%20R%5BAuthor%5D&cauthor=true&cauthor_uid=21940231). |
| **Journal & Publishing information** | | [Women Birth.](http://www.ncbi.nlm.nih.gov/pubmed/21940231) 2012 Sep;25(3):e27-36. doi: 10.1016/j.wombi.2011.08.009. Epub 2011 Sep 22. |
| **Abstract** | | **Objective:** Midwives’ ability to manage maternal deterioration and ‘failure to rescue’ are of concern with questions over knowledge, clinical skills and the implications for maternal morbidity and, mortality rates. In a simulated setting our objective was to assess student midwives’ ability to assess, and manage maternal deterioration using measures of knowledge, situation awareness and skill, performance.  **Methods:** Anexploratoryquantitativeanalysisofstudentperformancebaseduponperformance, ratings derived from knowledge tests and observational ratings. During 2010 thirty-five student, midwives attended a simulation laboratory completing a knowledge questionnaire and two video, recorded simulated scenarios. Patient actresses wearing a ‘birthing suit’ simulated deteriorating, women with post-partum and ante-partum haemorrhage (PPH and APH). Situation awareness was, measured at the end of each scenario. Applicable descriptive and inferential statistical tests were, applied to the data.  **Findings:** The mean total knowledge score was 75% (range 46—91%) with low skill performance, means for both scenarios 54% (range 39—70%). There was no difference in performance between the scenarios, however performance of key observations decreased as the women deteriorated; with significant reductions in key vital signs such as blood pressure and blood loss measurements. Situation, awareness scores were also low (54%) with awareness decreasing significantly (t(32) = 2.247, p = 0.032), in the second and more difficult APH scenario.  **Conclusion:** Whilst knowledge levels were generally good, skills were generally poor and decreased as the women deteriorated. Such failures to apply knowledge in emergency stressful situations may be resolved by repetitive high stakes and high fidelity simulation. |
| **Presentation**  **(**Information presented to learners) | What? The content of the presentation / practice scenarios / feedback ... | The ability of staff to identify and manage deterioration in the ante/post-natal woman has not been closely examined and there is a lack of understanding about how inexperienced clinicians make decisions. Situation awareness does appear to play an important role and simulayion training has been shown to improve knowledge and clinical skills in labour room staff.  With these issues in mind this paper follows our previous work on the management of deteriorating patients and is part of a series of studies examining deterioration management in simulated settings using manikins, simulated patients and in situ simulation.  With these issues in mind we examined the pre-existing knowledge and skills of Australian student midwives in video recorded simulated settings incorporating clinical and situation awareness assessments. *pg e28* |
|  | How? The form of the presentation / practice scenarios / feedback ... (e.g., sequencing, scaffolds etc.) | We therefore utilized a simulated environment, to measure skill and situation awareness, in order to investigate and enhance student midwives’ ability to assess and manage deteriorating women. *pg e29* |
|  | Medium? The medium used (on paper, lecture, group discussion, simulation etc.) |  |
| **Practice**  **(**Practice provided to learners) | What? The content of the presentation / practice scenarios / feedback ... |  |
|  | How? The form of the presentation / practice scenarios / feedback ... (e.g., sequencing, scaffolds etc.) | Participants attended a 1.5 h individual session which included a demographic survey, knowledge test, two 8 min simulation exercises and video based reflective review and feedback. *pg e29*  Four professional patient actresses were employed to simulate clinical scenarios. In the PPH scenario a ‘newborn baby’ was provided in a cot and the actresses were fitted with the ‘ModelMed Birth Simulation Suit’. The suit simulates a post-partum abdomen, including palpation of a boggy uterus, realistic internal examinations, and haemorrhage of blood-like fluid from the vagina. In the APH scenario the actress donned a pregnancy suit with a firm uterus. Scenarios were drawn from maternal records and contemporary guidelines of the Royal Womens’ Hospital, Melbourne and were assessed by a panel of clinical experts to ensure face and content validity. The neonatal nurse educator on the team was also an experienced midwife who was able to advise generally and specifically on birthing and resuscitation practices. In order to mimic reality as closely as possible, the conduct of the simulations met three conditions: 1, relatively little information was provided initially; 2, the participants were allowed to investigate freely and 3, were given clinical information over time; process-based information giving.  In both scenarios subtle cues indicating maternal deterioration were present in the first 4 min, prior to more obvious and significant deterioration in maternal condition during the final 4 min (i.e. the women deteriorated significantly at the 4 min mark). On arrival participants were provided with a verbal description of the woman’s presenting condition, e.g. ‘a 28-year old has had a spontaneous vaginal birth of her first baby at term’ and minimal further information, requiring observations or actions to be performed on the basis of the presenting and developing condition. *pg e30* |
|  | Medium? The medium used (on paper, lecture, group discussion, simulation etc.) |  |
| **Feedback**  **(**Feedback given to learners) | What? The content of the presentation / practice scenarios / feedback ... |  |
|  | How? The form of the presentation / practice scenarios / feedback ... (e.g., sequencing, scaffolds etc.) | We measured students’ level of knowledge (prior to the simulation exercises), rated their clinical performance during two simulations with the aid of video records, and measured their level of ‘situation awareness’. *pg e29*  All participants complete two 8 min contrasting videoed simulation exercises (PPH and APH) in a skills laboratory designed to simulate a maternity ward. In order therefore to reduce coercion potential participants were informed that results would not influence degree or registration outcomes. In addition applicable feedback and/or training was offered to participants and non-participants on completion of the study. The supportive role of the team and the debriefing and feedback components of the study were emphasised. *pg e30* |
|  | Medium? The medium used (on paper, lecture, group discussion, simulation etc.) |  |
| **Assessment**  **(**Assessing the learners) | What? The content of the presentation / practice scenarios / feedback ... | Participant reflective review followed the scenarios prior to final performance feedback, with the ultimate aim of improving clinical performance and patient safety. We report on the quantitative outcomes of this study. *pg e28*  In summary, we used simulation to identify student midwives ability to manage deteriorating women. We measured students’ level of knowledge (prior to the simulation exercises), rated their clinical performance during two simulations with the aid of video records, and measured their level of ‘situation awareness’. *pg e29*  At the conclusion of each scenario the participants were removed from the simulation area and asked 15 ‘yes/no’ questions regarding the situation. The questions were developed by a panel of experts using Goal Directed Task Analysis to identify goals, decisions and situation awareness requirements. **Questions for each scenario covered three domains (i) physiological perception [5 items], e.g. ‘what is the current blood pressure?’ (ii) Comprehension [5 items], e.g. ‘what is wrong with the woman?’ (iii) Projection [5 items], e.g. ‘what future medications may be required?’ Immediate ‘gut reaction’ responses were encouraged.** The total score was calculated from the number of correctly answered questions. *pg e30* |
|  | How? The form of the presentation / practice scenarios / feedback ... (e.g., sequencing, scaffolds etc.) | The 11 item multiple choice questionnaire (with 4 response options), was used to assess participants’ knowledge of maternal deterioration. ... Items related to management of obstetric emergencies (n = 5) and observational parameters (n = 6). The percentage of correctly answered items was calculated for each participant. Skill ratings were performed by this researcher and a second non-participant observer. Consultation and consensus was then reached at the end of each scenario to ensure inter-rater reliability. A standardised checklist was used to rate the number of correct observations/actions and the mean performance was calculated for each participant (out of 23 possible observations/actions for each scenario). *pg e29/30* |
|  | Medium? The medium used (on paper, lecture, group discussion, simulation etc.) |  |

- 1. **Articles' Tables PPH Simulation & ID Subset8to14**

| **Paper # 8 Egenberg2015** | | |
| --- | --- | --- |
| **Title** | | Can inter-professional simulation training influence the frequency of blood transfusions after birth? |
| **Authors** | | [Egenberg S](http://www.ncbi.nlm.nih.gov/pubmed/?term=Egenberg%20S%5BAuthor%5D&cauthor=true&cauthor_uid=25545119)^1^, [Øian P](http://www.ncbi.nlm.nih.gov/pubmed/?term=%C3%98ian%20P%5BAuthor%5D&cauthor=true&cauthor_uid=25545119), [Bru LE](http://www.ncbi.nlm.nih.gov/pubmed/?term=Bru%20LE%5BAuthor%5D&cauthor=true&cauthor_uid=25545119), [Sautter M](http://www.ncbi.nlm.nih.gov/pubmed/?term=Sautter%20M%5BAuthor%5D&cauthor=true&cauthor_uid=25545119), [Kristoffersen G](http://www.ncbi.nlm.nih.gov/pubmed/?term=Kristoffersen%20G%5BAuthor%5D&cauthor=true&cauthor_uid=25545119), [Eggebø TM](http://www.ncbi.nlm.nih.gov/pubmed/?term=Eggeb%C3%B8%20TM%5BAuthor%5D&cauthor=true&cauthor_uid=25545119). |
| **Journal & Publishing information** | | [Acta Obstet Gynecol Scand.](http://www.ncbi.nlm.nih.gov/pubmed/25545119) 2015 Mar;94(3):316-23. doi: 10.1111/aogs.12569. Epub 2015 Feb 1. |
| **Abstract** | | **Objective.** To investigate whether inter-professional simulation training influenced the rate of red blood cell (RBC) transfusions after birth. **Design.** Two cohorts were compared retrospectively using a pre–post design. Setting. Norwegian university hospital with 4800 deliveries annually. Population. Women with estimated blood loss >500 mL within 24 h after birth in 2009 and 2011. **Methods.** In 2010, all maternity staff attended a 6-h, scenario-based training on emergency obstetrics including postpartum hemorrhage, using a birthing simulator. The simulation focused on prevention, identification, and treatment of postpartum hemorrhage and on communication and leadership. Debrief immediately after the scenarios involved reflection and self-assessment. **Main outcome measures.** The frequency of women receiving RBC transfusions as a marker for blood loss. Secondary out- come was the frequency of surgical procedures in the management of postpartum hemorrhage. **Results.** In 2009, 111/534 (20.8%) women with estimated blood loss >500 mL after birth received RBC transfusions vs. 67/546 (12.3%) in 2011 (p < 0.01). The adjusted odds ratio for women receiving RBC transfusions in 2011 vs. 2009 was 0.53 (95% CI 0.38–0.74). Parity, oxytocin augmentation, duration of second stage, episiotomy, operative vaginal delivery, and sphincter injury were included in the final model. The odds ratio was stable in all combinations of possible confounders. We observed a significant reduction in the frequencies of curettage (p < 0.01) and uterine artery embolizations (p = 0.01). **Conclusion.** We found a significant reduction in RBC transfusions after birth, which might be associated with mandatory simulation training. A causal link cannot be documented because of complex interactions of several variables. **Abbreviations:** CS, cesarean section; Hgb, hemoglobin; PPH, postpartum hemorrhage; RBC, red blood cell. |
| **Presentation**  **(**Information presented to learners) | What? The content of the presentation / practice scenarios / feedback ... | The simulation training comprised lectures and discussions on local guidelines for obstetric emergencies, such as PPH with a focus on atony being the major cause of PPH, pre-defined learning objectives, scenarios with role play, and reflections in inter-professional teams. *pg 317* |
|  | How? The form of the presentation / practice scenarios / feedback ... (e.g., sequencing, scaffolds etc.) | In 2010, the hospital implemented a yearly, mandatory, 6-h inter-professional training for 180 midwives, doctors, and nurse assistants in the maternity wards. *pg 317* |
|  | Medium? The medium used (on paper, lecture, group discussion, simulation etc.) |  |
| **Practice**  **(**Practice provided to learners) | What? The content of the presentation / practice scenarios / feedback ... | The scenario-based training on PPH involved small teams being exposed to scenarios on excessive blood loss after birth.  The simulation focused on prevention, identification, and treatment of postpartum hemorrhage and on communication and leadership. *pg 317* |
|  | How? The form of the presentation / practice scenarios / feedback ... (e.g., sequencing, scaffolds etc.) | One essential difference between the training at Stavanger University Hospital and similar exercises in other hospitals was the yearly, mandatory 6-h training for the entire maternity staff using the birthing simulator MamaNatalie, which was newly developed and offered a highly realistic way to simulate excessive bleeding. *pg 321* |
|  | Medium? The medium used (on paper, lecture, group discussion, simulation etc.) | The scenario-based training on PPH involved small teams being exposed to scenarios on excessive blood loss after birth. A fellow midwife using the strap-on birthing simulator “MamaNatalie” (Laerdal Global Health, Stavanger, Norway) acted as a laboring woman (Figure 1) featuring a possible blood loss ≤1200 mL and bleeding in a manner corresponding with atony after birth. Uterine massage was an important part of the protocol on PPH and was emphasized in all PPH scenarios as contributing to the prevention of excessive blood loss. According to the protocol, the midwife consults the obstetrician on duty when estimated blood loss exceeds 1000 mL. Simulation training focused on the algorithm regarding PPH, where the midwife was supposed to perform active management of the third stage of labor, including thorough uterine massage and routine administration of oxytocin. *pg 317/318* |
| **Feedback**  **(**Feedback given to learners) | What? The content of the presentation / practice scenarios / feedback ... |  |
|  | How? The form of the presentation / practice scenarios / feedback ... (e.g., sequencing, scaffolds etc.) | Another important factor was the review sessions involving reflection and self-assessment immediately after the scenario. Self-assessment has the highest impact on learning. The learning objectives in the simulation training were actions according to PPH protocol, including appropriate communication between staff members. Debriefing may have stimulated self-evaluation and group reflection on how to prevent, identify, and treat PPH most efficiently, resulting in more effective handling of PPH situations. *pg 322* |
|  | Medium? The medium used (on paper, lecture, group discussion, simulation etc.) |  |
| **Assessment**  **(**Assessing the learners) | What? The content of the presentation / practice scenarios / feedback ... | After running PPH scenarios, the teams were challenged on visual estimation of the blood loss. In cases of ongoing hemorrhage, the midwife is supposed to call for help, administer a high-dose oxytocin infusion, give misoprostol rectally, empty the urinary bladder, and repair bleeding perineal tears. In case of excessive blood loss, the protocol recommends bimanual compression of the uterus and transfer of the mother to the operating theater. Scenario-based training was limited to the emergency situation and the treatment given before transfer to the operating theatre. According to protocol, curettage and, if needed, uterine artery embolization (available round-the-clock) were to be carried out in the operating theater if excessive bleeding remained. For women with severe PPH after cesarean section, B-Lynch sutures were recommended. Hysterectomy would be the last option, performed only as a life-saving procedure. *pg 318* |
|  | How? The form of the presentation / practice scenarios / feedback ... (e.g., sequencing, scaffolds etc.) | The facilitators, being fellow midwives trained to facilitate multi-professional teams, challenged the groups on their experiences, understanding, and actions, with the aim of strengthening self-efficacy and adherence to local procedures to prevent human errors. *pg 318* |
|  | Medium? The medium used (on paper, lecture, group discussion, simulation etc.) |  |
|  | | |
| **Paper # 9 Fialkow** | | |
| **Title** | | An in situ standardized patient-based simulation to train postpartum hemorrhage and team skills on a labor and delivery unit. |
| **Authors** | | [Fialkow MF](http://www.ncbi.nlm.nih.gov/pubmed/?term=Fialkow%20MF%5BAuthor%5D&cauthor=true&cauthor_uid=24401919)^1^, [Adams CR](http://www.ncbi.nlm.nih.gov/pubmed/?term=Adams%20CR%5BAuthor%5D&cauthor=true&cauthor_uid=24401919), [Carranza L](http://www.ncbi.nlm.nih.gov/pubmed/?term=Carranza%20L%5BAuthor%5D&cauthor=true&cauthor_uid=24401919), [Golden SJ](http://www.ncbi.nlm.nih.gov/pubmed/?term=Golden%20SJ%5BAuthor%5D&cauthor=true&cauthor_uid=24401919), [Benedetti TJ](http://www.ncbi.nlm.nih.gov/pubmed/?term=Benedetti%20TJ%5BAuthor%5D&cauthor=true&cauthor_uid=24401919), [Fernandez R](http://www.ncbi.nlm.nih.gov/pubmed/?term=Fernandez%20R%5BAuthor%5D&cauthor=true&cauthor_uid=24401919). |
| **Journal & Publishing information** | | [Simul Healthc.](http://www.ncbi.nlm.nih.gov/pubmed/24401919) 2014 Feb;9(1):65-71. doi: 10.1097/SIH.0000000000000007. |
| **Abstract** | | *No Abstract* |
| **Presentation**  **(**Information presented to learners) | What? The content of the presentation / practice scenarios / feedback ... | The clinical care needs assessment was performed by evaluating current standards of practice for patient care during bleeding emergencies. The authors conducted an in-depth literature review and evaluation of current local hospital protocols. Critical components were included in the learning objectives (Fig. 1). *pg 67* At the start of the simulation, all (or a portion) of the participants can be read the following case stem. The decision on who should receive the stem depends on desired learning objectives, the skills of the learners, and the systems-based practices at the training institution. *pg 68* |
|  | How? The form of the presentation / practice scenarios / feedback ... (e.g., sequencing, scaffolds etc.) |  |
|  | Medium? The medium used (on paper, lecture, group discussion, simulation etc.) |  |
| **Practice**  **(**Practice provided to learners) | What? The content of the presentation / practice scenarios / feedback ... | An event-based approach to training uses discreet events placed strategically throughout the course of the simulation to ensure that desired behaviors and team interactions are triggered in a realistic manner. Standardized patient, confederate, and clinical events (bleeding, vital sign changes, etc) served as triggers throughout the scenario. The content of the triggers and events was informed by the needs assessment and are listed in Figure 1. The decision to use a standardized patient-based format was multifactorial. First, because one of the objectives of the simulation centered on communication with the patient and patient-centered care, realistic patient communication cues were essential. Second, because performance of procedural skills and more invasive management were not part of the simulation, adequate fidelity could be obtained using simulation adjuncts (simulated intravenous hook-up, vaginal bleeding with PartoPants, etc). Finally, the use of standardized patients for similar roles has been supported in the literature. *pg 67* The scenario clinical events and patient clinical course, clinical indicators and triggers, and expected patient care and teamwork actions were content validated by independent health provider subject matter experts (board-certified obstetricians and L&D nurses with 95 years of experience). The methodology of content validation involved having a sample of subject matter experts rate the extent to which the event content (including sequencing, timing, and requisite responses) appropriately represents the typical resuscitation problem it is designed to simulate (Fig. 2 for example). A sample of 17 subject matter experts (9 obstetrical nurses, 8 obstetricians) was used to meet professional standards for content validation (6-8 experts per area of evaluation). Content rating for appropriate representativeness used a 5-point Likert scale (ranging from 1, strongly agree, to 5, strongly disagree). The mean rating was 1.84, indicating strong endorsement by the subject matter experts. Indices for overall agreement rwg(j) (0.98) indicated good subject matter expert convergence that scenario events, clinical indicators, and targeted medical actions were representative and met appropriate standards for patient care. This convergence, in combination with the high rating mean, substantiated the content validity of the scenario. *pg 67/68* |
|  | How? The form of the presentation / practice scenarios / feedback ... (e.g., sequencing, scaffolds etc.) | This scenario can be performed in situ or in a simulation center. To optimize our ability to evaluate teamwork and systems factors in our simulation, we conducted simulations on an L&D unit. Phases 1 to 3 were conducted in a standard labor, delivery, and resuscitation room, and the patient was transferred to an operating room for phases 4 to 5 (Fig. 1). All parts of the scenario could be performed in a single location if appropriate for the institution’s practice.  Instructors  Instructors (2 needed) were board-eligible obstetricians and licensed, certified obstetrical nurses with instructional experience in simulation training and debriefing. .. In addition, each simulation required instructors to assist standardized patients with their verbal prompts by giving reminders through a 1-way radio/headset. This role can be important because teams often use unanticipated strategies that nonclinical standardized patients and nurse confederates may not be prepared to address in a manner that is both re- alistic and in line with the goals of the simulation. For example, more aggressive management (ie, invasive monitoring or surgi- cally based treatments) would not be possible on a standardized patient. When instructors hear such planning occurring, they can give the confederates realistic responses (‘‘I cannot consent to any procedures without my husband present.’’) that allow the scenario to continue without pointing out obvious technical limitations. Having standardized patients recall all timed prompts and respond to such unanticipated learner comments is quite challenging and is facilitated by the ability of an instructor to talk through the response with the standardized patient in real time.  Schedule: Training sessions lasted for 2 hours in total and involved the following: Sign in (5 minutes), Introduction to simulation environment and PartoPants (15 minutes), Simulation session (20 minutes), Break (10 minutes), Debrief and feedback to instructors (50 minutes), Wrap-up (5 minutes).  All (or a portion) of the participants can then be directed to the patient’s bedside. At this time, the patient’s chart is available if requested (including laboratory values from her emergency department stay). The patient is able to provide any necessary medical history. The degree of spontaneously provided information can differ depending on the skill set of the learners.  As the team progresses through the case, they may call for help from other members if not initially allowed into the room. This allows for potential changes in leadership, handoffs, and briefs depending on the system in place at the training institution. Updated bedside ultrasound and bimanual examination findings are available to the team as they are requested. The team may also transfer the patient to the operating room if surgical intervention is anticipated. The case ends with stabilization of the patient. |
|  | Medium? The medium used (on paper, lecture, group discussion, simulation etc.) | A 34-year-old G3, P2 female has just been admitted through the emergency department after a precipitous delivery and subsequent postpartum hemorrhage from uterine atony, now controlled after uterotonics (40 U oxytocin IV). Patient’s estimated blood loss is 1.5 L before arrival. She has been given 1 U of packed red blood cells and 2 L of crystalloids. Type and cross match has been performed. (Her laboratory values from the emergency department are available on a chart at the bedside if requested.) *pg 68*  Nurse Confederate: The nurse confederate served to introduce the team to the patient and to help teams provide appropriate care within the simulated environment. She provided assistance when asked but does not help with medical knowledge or clinical care direction. The nurse confederate was also tasked with providing ‘‘backup triggers’’ to ensure the team progresses through the entire scenario as written (Fig. 1). By providing such triggers, the instructors guarantee that the learners have the opportunity to meet all learning objectives. Because the goal was to have the team (not the nurse confederate) complete most tasks, she was introduced as a new, that is, inexperienced, nurse transporting the patient from the emergency department. To ensure consistency and accuracy of role fulfillment, standardized patients received 20 hours of training. Confederate performance was assessed before initiating simulations and throughout the trainings by the investigators (C.R.A., L.C., M.F.F.) who reviewed video recordings and provided focused feedback.  Standardized Patient: The standardized patient role required a female, age 20 to 40 years. The standardized patient also provided critical triggers and prompts to the team (Fig. 1). As the simulation progressed and the patient’s condition worsened, the standardized patient became more anxious and concerned with her clinical status. Her vital signs were provided using a simulated monitor, and the low-to-high volume vaginal bleeding was simulated using PartoPants. The standardized patient controlled the rate of bleeding via a 3-way stopcock. |
| **Feedback**  **(**Feedback given to learners) | What? The content of the presentation / practice scenarios / feedback ... |  |
|  | How? The form of the presentation / practice scenarios / feedback ... (e.g., sequencing, scaffolds etc.) | Both physician and nursing instructors were present at each debriefing to ensure a balanced, interprofessional discussion of team-work issues.  Debriefings were conducted using methodology outlined by Brett-Fleeger et al. First, instructors set the tone for psychological safety within the debrief by establishing clear expectations of respect for and between the learners and by reinforcing a standard confidentiality agreement that is part of all simulation-based training at the University of Washington. This was followed by an open question to the participants to elicit general thoughts about the simulation experience. These were noted by one of the instructors acting as a scribe. Next, a short period was spent reviewing the medical knowledge and diagnostic issues raised during the simulation. Specific critical components of the bleeding protocol were discussed, and participants were encouraged to reflect on their performance and any barriers to effectiveness they noted. Instructors also ensured that the process of estimating blood loss and indications for operative management were reviewed.  After this part of the debriefing, instructors led partici- pants toward discussion of teamwork behaviors. Any communication or teamwork issues identified during the first part of the debrief were further explored and deconstructed to see if any themes emerged (see Results). In addition, the instructors used clips from the simulation video to allow participants to observe themselves and reflect specifically on key aspects of (1) leadership and leadership transition, (2) provider-patient communication, (3) role assignment, and (4) the use of team updates to ensure a shared mental model. Before leaving the training, learners completed a training evaluation used by the University of Washington Institute for Simulation and Interprofessional Studies. |
|  | Medium? The medium used (on paper, lecture, group discussion, simulation etc.) |  |
| **Assessment**  **(**Assessing the learners) | What? The content of the presentation / practice scenarios / feedback ... | This simulation was overall well received. Results from the postsimulation survey, along with participant response rates, are presented in Table 3. *See Table 3 in the article. pg 70* |
|  | How? The form of the presentation / practice scenarios / feedback ... (e.g., sequencing, scaffolds etc.) |  |
|  | Medium? The medium used (on paper, lecture, group discussion, simulation etc.) |  |
|  | | |
| **Paper # 10 Magee** | | |
| **Title** | | Low cost, high yield: simulation of obstetric emergencies for family medicine training. |
| **Authors** | | [Magee SR](http://www.ncbi.nlm.nih.gov/pubmed/?term=Magee%20SR%5BAuthor%5D&cauthor=true&cauthor_uid=23848326)^,^ [Shields R](http://www.ncbi.nlm.nih.gov/pubmed/?term=Shields%20R%5BAuthor%5D&cauthor=true&cauthor_uid=23848326), [Nothnagle M](http://www.ncbi.nlm.nih.gov/pubmed/?term=Nothnagle%20M%5BAuthor%5D&cauthor=true&cauthor_uid=23848326). |
| **Journal & Publishing information** | | [Teach Learn Med.](http://www.ncbi.nlm.nih.gov/pubmed/23848326) 2013;25(3):207-10. doi: 10.1080/10401334.2013.797353 |
| **Abstract** | | ***Background*:** Simulation is now the educational standard for emergency training in residency and is particularly useful on a labor and delivery unit, which is often a stressful environment for learners given the frequency of emergencies. However, simulation can be costly. ***Purpose*:** This study aimed to assess the feasibility and effectiveness of low-cost simulated obstetrical emergencies in training family medicine residents. ***Methods*:** The study took place in a community hospital in an urban underserved setting in the Northeast United States. Low cost simulations were developed for postpartum hemorrhage (PPH) and preeclampsia/eclampsia (PEC). Twenty residents were randomly assigned to the intervention (simulated PPH or PEC followed by debriefing) or control (lecture on PPH or PEC) group, and equal numbers of residents were assigned to each scenario. All participants completed a writting test at baseline and an oral exam 6 months later on the respective scenario to which they were assigned. The participants provided written feedback on their respective teaching interventions. We compared performance on pretest and posttests by group using Wilcoxon Rank Sum. ***Results*:** Twenty residents completed the study. Both groups performed similarly on baseline tests for both scenarios. Compared to controls, intervention residents scored significantly higher on the examination on the management of PPH but not for PEC. All intervention group participants reported that the simulation training was “extremely useful,” and most found it “enjoyable.” ***Conclusions*:** We demonstrated the feasibility and acceptability of two low-cost obstetric emergency simulations and found that they may result in persistent increases in trainee knowledge. **Keywords:** simulation in healthcare, simulation in obstetrics, simulation |
| **Presentation**  **(**Information presented to learners) | What? The content of the presentation / practice scenarios / feedback ... |  |
|  | How? The form of the presentation / practice scenarios / feedback ... (e.g., sequencing, scaffolds etc.) | The control group received only the traditional teaching in the form of a 1-hour lecture by a faculty member.  See Table 1 for a summary chart comparing the activities of the intervention and control groups. *pg 208* |
|  | Medium? The medium used (on paper, lecture, group discussion, simulation etc.) |  |
| **Practice**  **(**Practice provided to learners) | What? The content of the presentation / practice scenarios / feedback ... | Residents were randomly assigned to intervention or control group. Within each group, residents were assigned to learn about postpartum hemorrhage or preeclampsia/eclampsia. *pg 208* |
|  | How? The form of the presentation / practice scenarios / feedback ... (e.g., sequencing, scaffolds etc.) | All participants completed a written test at baseline and an oral exam 6 months later on the respective scenario to which they were assigned. *(abstract)* Each resident in the intervention group participated individually in a simulation exercise involving a postpartum hemorrhage or an eclamptic seizure. These simulations occurred only once for each resident. *pg 208* |
|  | Medium? The medium used (on paper, lecture, group discussion, simulation etc.) | The low-cost simulation program involved emergency scenarios using volunteer faculty actors. Faculty members in family medicine obstetrics as well as an outside consultant expert in simulation created the scripts for the simulation scenarios and a scoring tool to evaluate the residents’ performance in each simulation exercise. After we trained all members of the simulation team (actors and scorers), we piloted the scenarios with two senior 3rd-year residents and made minor changes to the scripts. Each simulation took about 30 minutes to set up and then 20 to 30 minutes to perform with the learners. These simulations occurred approximately once monthly on labor and delivery over an 18-month period. The researcher initiated each scenario by calling nurses and residents to a labor room to evaluate a “patient”; on arrival the trainee learned the encounter was a simulation. One scenario simulated a postpartum hemorrhage. The actor sat behind a plastic pelvis commonly used by obstetrical training programs and covered herself with a sheet. Using an IV bag with red food coloring injected into it to give the appearance of blood, the actor squeezed out fluid manually to simulate an active hemorrhage. In a scenario for eclampsia, the actor simulated symptoms of preeclampsia and then an eclamptic seizure, and the team of physicians and nurses treated her accordingly. As much as possible, the simulations attempted to mimic reality. Two faculty were needed for each simulation—one as the actor and one as the scorer. *pg 208* |
| **Feedback**  **(**Feedback given to learners) | What? The content of the presentation / practice scenarios / feedback ... | The participants provided written feedback on their respective teaching interventions. *(abstract)* |
|  | How? The form of the presentation / practice scenarios / feedback ... (e.g., sequencing, scaffolds etc.) | After the simulation, the actors, the scorers, the nurses, and the residents met for up to 30 minutes to debrief.  The attending actor in the simulation led the debriefing session and encouraged the trainee to reflect on his or her performance of the target behaviors. Simulation and debriefing took approximately 1 hour in total. At the end of the debriefing, the leader gave each resident a short questionnaire in which participants ranked the usefulness of the simulation as a training tool and their overall satisfaction with the experience. In addition, there was space for open-ended comments.  The attending actor in the simulation led the debriefing session and encouraged the trainee to reflect on his or her performance of the target behaviors. Simulation and debriefing took approximately 1 hour in total. At the end of the debriefing, the leader gave each resident a short questionnaire in which participants ranked the usefulness  After the simulation, the actors, the scorers, the nurses, and comments. *pg 209* |
|  | Medium? The medium used (on paper, lecture, group discussion, simulation etc.) |  |
| **Assessment**  **(**Assessing the learners) | What? The content of the presentation / practice scenarios / feedback ... | Simulation scoring sheets included eleven target behaviors for the scorer to identify during the simulation (Table 2). A resident was given full credit for a behavior if he or she asked for any intervention in the chosen category. For instance, for the postpartum hemorrhage simulation, if the learner chose any treatment medication from the list of appropriate options (pitocin, methergine, prostaglandin F2alpha, or misoprostol), he or she was given full credit for choosing an appropriate medication. There was no partial credit (if the resident could not identify the name but knew that medications should be used) or extra credit given (if the resident identified all four medication possibilities available in our hospital). *pg 208/209* |
|  | How? The form of the presentation / practice scenarios / feedback ... (e.g., sequencing, scaffolds etc.) | Both groups were pretested using a 20-item multiple-choice test on either postpartum hemorrhage or preeclampsia. *pg 208*  Both groups (control and intervention) were then posttested 6 months later using a case-based oral examination on the scenario to which they had been assigned. Both groups completed written teaching evaluations using a 5-point Likert scale and intervention participants also wrote responses to open ended questions about the simulation experience.  Simulation scoring sheets included eleven target behaviors for the scorer to identify during the simulation (Table 2). *pg 209* |
|  | Medium? The medium used (on paper, lecture, group discussion, simulation etc.) |  |
|  | | |
| **Paper # 11 Markova** | | |
| **Title** | | Evaluation of multiprofessional obstetric skills training for postpartum hemorrhage |
| **Authors** | | Markova V, Sørensen JL, Holm C, Nørgaard A, Langhoff-Roos J. |
| **Journal & Publishing information** | | Acta Obstet Gynecol Scand. 2012 Mar;91(3):346-52. doi: 10.1111/j.1600-0412.2011.01344.x. |
| **Abstract** | | ***Objective.*** To evaluate the effect of multi-professional obstetric skills training on the incidence of postpartum hemorrhage (PPH) indicated by red blood cell (RBC) transfusion and time delay in surgical interventions before, during, and after implementation of the training. ***Design*.** A database audit. ***Setting*.** University hospital, Rigshospitalet, Copenhagen, Denmark. ***Population*.** Women receiving red blood cell (RBC) transfusion up to seven days postpartum before (2003), during (2005), and after (2007) the introduction of training. ***Methods*.** Linkage of the Danish Medical Birth Registry and the local transfusion database, followed by audit of medical records. We identified 148 women with RBC transfusion for PPH in 10 461 deliveries and assessed the cause of PPH, surgical interventions and transfusion data. ***Main outcome measures*.** RBC transfusion. Delayto surgical intervention. ***Results*.** RBC transfusion rates for PPH were 1.5% (2003), 1.6% (2005), and 1.2% (2007) (not statistically significant). The transfusion rates did not change after vaginal delivery but decreased after cesarean section [2.4, 2.1 and 0.7% (*p*<0.01)]. Transfusion requirements and pre-transfusion hemoglobin values did not change. The median time from delivery to manual removal of the placenta increased non-significantly (64, 70 and 75 minutes). The median time from decision to manual removal of the placenta remained unchanged (30 minutes). ***Conclusion*.** There was no effect of multi-professional obstetric skills training on the rate of RBC transfusion for PPH. The unchanged long delay in handling a retained placenta indicates a need for multi-disciplinary training in collaboration with staff from anesthesiology and the operation theater. **Abbreviations:** PPH, postpartum hemorrhage; RBC, red blood cell; CS, cesarean section; Hb, hemoglobin, mmol/L (conversion algorithm: 1mmol/L=16g/L). |
| **Presentation**  **(**Information presented to learners) | What? The content of the presentation / practice scenarios / feedback ... | The introduction of simulation-based multi-professional obstetric skills training for PPH started with a focus on basic skills in managing PPH at and after vaginal delivery. The training sessions were mandatory for midwives, nurses, auxiliary nurses and on-call doctors in the obstetric department. *pg 347* |
|  | How? The form of the presentation / practice scenarios / feedback ... (e.g., sequencing, scaffolds etc.) | Each 2.5-hour training session included: (1) a theoretical lecture for 12 participants from different staff groups...  The training sessions were (and still are) held regularly to include all staff, and members are invited to repeat the course every two to three years. *pg 347* |
|  | Medium? The medium used (on paper, lecture, group discussion, simulation etc.) |  |
| **Practice**  **(**Practice provided to learners) | What? The content of the presentation / practice scenarios / feedback ... | In December 2003 the obstetric department at Copenhagen University Hospital (Rigshospitalet), introduced multi- professional obstetric skills training in the management of emergency obstetric situations which may occur in conjunction with vaginal delivery, e.g. management of postpartum hemorrhage (PPH), shoulder dystocia, basic neonatal resuscitation, and severe preeclampsia. Midwives, nurses, auxiliary nurses and doctors on call participated.  The training focused on prophylaxis, identification of PPH and estimation of blood loss, administration of uterotonics, fluid replacement and compression of the uterus but did not focus on how and when to give RBC transfusions.  The scenarios were based on vaginal deliveries only. *pg 346* |
|  | How? The form of the presentation / practice scenarios / feedback ... (e.g., sequencing, scaffolds etc.) | Each 2.5-hour training session included: (1) a theoretical lecture for 12 participants from different staff groups, (2) a multi-professional workshop for groups of six participants where scenario-based skills training was provided, and (3) discussion and feedback for the whole group. *pg 347* |
|  | Medium? The medium used (on paper, lecture, group discussion, simulation etc.) | Techniques were taught on mannequins. *pg 347* |
| **Feedback**  **(**Feedback given to learners) | What? The content of the presentation / practice scenarios / feedback ... |  |
|  | How? The form of the presentation / practice scenarios / feedback ... (e.g., sequencing, scaffolds etc.) | Each 2.5-hour training session included: ..., and (3) discussion and feedback for the whole group. *pg 347* |
|  | Medium? The medium used (on paper, lecture, group discussion, simulation etc.) |  |
| **Assessment**  **(**Assessing the learners) | What? The content of the presentation / practice scenarios / feedback ... | The evaluation of this intervention showed a significant increase in self-assessed confidence among auxiliary nurses and trainee doctors in management of PPH, a significant increase in the administration of uterotonics and the use of the ICD-code for PPH, as well as a facilitation of staff-directed infrastructural changes in the delivery room, such as emergency boxes with relevant medicines and equipment for the management of PPH. *pg 346* |
|  | How? The form of the presentation / practice scenarios / feedback ... (e.g., sequencing, scaffolds etc.) |  |
|  | Medium? The medium used (on paper, lecture, group discussion, simulation etc.) |  |
|  | | |
| **Paper # 12 Marshal** | | |
| **Title** | | Impact of simulation and team training on postpartum hemorrhage management in non-academic centers. |
| **Authors** | | [Marshall NE](http://www.ncbi.nlm.nih.gov/pubmed/?term=Marshall%20NE%5BAuthor%5D&cauthor=true&cauthor_uid=24824110)^1^, [Vanderhoeven J](http://www.ncbi.nlm.nih.gov/pubmed/?term=Vanderhoeven%20J%5BAuthor%5D&cauthor=true&cauthor_uid=24824110), [Eden KB](http://www.ncbi.nlm.nih.gov/pubmed/?term=Eden%20KB%5BAuthor%5D&cauthor=true&cauthor_uid=24824110), [Segel SY](http://www.ncbi.nlm.nih.gov/pubmed/?term=Segel%20SY%5BAuthor%5D&cauthor=true&cauthor_uid=24824110), [Guise JM](http://www.ncbi.nlm.nih.gov/pubmed/?term=Guise%20JM%5BAuthor%5D&cauthor=true&cauthor_uid=24824110). |
| **Journal & Publishing information** | | [J Matern Fetal Neonatal Med.](http://www.ncbi.nlm.nih.gov/pubmed/24824110) 2014 May 29:1-5. [Epub ahead of print] |
| **Abstract** | | **Objective:** Prompt recognition and response to postpartum hemorrhage (PPH) are vital in preventing maternal morbidity and mortality. We conducted a multi-center study to evaluate in situ simulation and team training for PPH among experienced clinical teams in non-academic hospitals in urban and rural communities. **Methods:** A longitudinal intervention study was performed in six Oregon community hospitals. All teams responded to an in situ simulated delivery and postpartum hemorrhage using trained actors and an obstetric birthing simulator, followed by a debriefing and training session. The simulation scenario was then repeated in 9–12 months. All sessions were digitally videorecorded and independently reviewed by two obstetricians using a structured evaluation form. PPH management including clinical response times were compared before and after team training using Student’s paired t-test and McNemar’s test. **Results:** Twenty-two teams completed paired case simulations. Team training significantly improved response times in management of PPH, including recognition of PPH, time to administer second medication. Medical management (use of the three indicated medications) improved after training from 27.3% to 63.6%, p=0.01. ***Conclusions:*** Simulation and team training significantly improved postpartum hemorrhage response times among clinically experienced community labor and delivery teams. **Keywords:** Clinical competence, education, outcome and process assessment, patient care team, postpartum hemorrhage, pregnancy complications, simulation |
| **Presentation**  **(**Information presented to learners) | What? The content of the presentation / practice scenarios / feedback ... | Clinical content covered included estimating blood loss, PPH risk factors, and medical and surgical treatments.  CRM/team training content included didactics and video expert modeling for the following topics: an overview of patient safety in obstetrics, communication (e.g. directed communication, transparent thinking and closed-loop communication), situational awareness, resource management, role clarity and leadership, and debriefing. *pg 2* |
|  | How? The form of the presentation / practice scenarios / feedback ... (e.g., sequencing, scaffolds etc.) | In addition, written materials were provided for each hospital to facilitate additional education in both teamwork and obstetric emergencies. We provided each site with a written CRM manual we created and a copy of the ACOG Obstetric Emergencies textbook as well. *pg 3* |
|  | Medium? The medium used (on paper, lecture, group discussion, simulation etc.) |  |
| **Practice**  **(**Practice provided to learners) | What? The content of the presentation / practice scenarios / feedback ... |  |
|  | How? The form of the presentation / practice scenarios / feedback ... (e.g., sequencing, scaffolds etc.) | The simulation scenario was then repeated in 9–12 months. *(abstract)* As has been previously described in detail, all simulation were performed in situ on the labor and delivery units of each institution to more realistically portray a typical hemorrhage that each team may experience. All participants were aware they would be involved in a simulated delivery but were not aware of the nature of the obstetric emergency. Clinical teams participated in groups that resembled the typical composition of response teams in their hospital and were composed of two labor and delivery nurses (one primary nurse and one secondary nurse who was available to be called in to assist) and one provider (obstetrician, certified nurse midwife or family physician). *pg 2* Teams participated in a second simulated PPH, again involving a hypertensive patient, 9–12 months after the initial simulation.Whenever possible, the same team members participated in both training scenarios. Although it was not mandatory to complete both sessions, all second visits included a minimum of two members who underwent the initial training. *pg 3* |
|  | Medium? The medium used (on paper, lecture, group discussion, simulation etc.) | An obstetric birthing simulator (NOELLE; Gaumard Scientific, Coral Gables, FL) was used to simulate PPH. Simulation personnel (study confederates), including a ‘‘hand-off nurse’’, ‘‘family member’’ and ‘‘patient’’ (who spoke for the obstetric simulator) followed a designated script that allowed for branch points based upon response.  At the beginning of the scenario, the hand-off (study confederate) nurse informed the primary nurse (participant) that the patient had a history of well-controlled hypertension (HTN), which made the use of methylergonovine contra-indicated. The obstetric provider (participant) received information directly from the participating nurse in accordance with local procedures. The use of a hypertensive simulated patient provided an opportunity to directly evaluate team communication and opportunities to prevent medication error. As only the primary nurse was informed of the patient’s history of HTN, it was essential for: (1) the nurse to inform the provider about the patient’s HTN, (2) the provider and/or nurse to recognize that HTN is a contraindication to methylergonovine administration and (3) the provider and/or nurse to not administer methylergonovine due to HTN. Even if the nurse did not initially inform the provider about the HTN, there was a second opportunity for the nurse to inform the provider about the patient history when the provider called for methylergonovine.  Simulated blood was dispensed directly through the manikin via large syringes controlled by the simulation (confederate) family member. Simulated maternal vital signs showed progressive maternal tachycardia and hypotension as the hemorrhage progressed along with resolution with appropriate clinical maneuvers, including uterine massage and medication administration. As these maneuvers can be both preventative and therapeutic, the time of initiation of the therapy and the time of recognition of hemorrhage were recorded. The administration of blood products was not included as an outcome measure due to the specific focus on prompt recognition of PPH and immediate preventative and therapeutic interventions. Providers were permitted to order any uterotonic medications available at their institution, which commonly included oxytocin, misoprostol, carboprost and methylergonovine. The simulation continued until PPH was corrected via medical management (i.e. the appropriate administration of three indicated uterotonic medications along with uterine massage) or 10min had elapsed. This time frame was chosen in order to allow sufficient education time and optimize participant focus on initial PPH management. If the team did not perform appropriate resuscitation measures after 10 min, the simulation was stopped and their time was not included in the assessment of time elapsed to correct PPH. *pg 2* |
| **Feedback**  **(**Feedback given to learners) | What? The content of the presentation / practice scenarios / feedback ... | Upon completion of the simulation all clinical teams received standardized debriefing and team training and reviewed a short, evidence-based, interprofessional clinical didactic. The didactic content consisted of a 7-min evidence-based clinical didactic and an 8-min team training video based upon TeamSTEPPS^TM^ and the book ‘‘Cockpit Resource Management"by Barbara Kanki. *pg 2* |
|  | How? The form of the presentation / practice scenarios / feedback ... (e.g., sequencing, scaffolds etc.) |  |
|  | Medium? The medium used (on paper, lecture, group discussion, simulation etc.) |  |
| **Assessment**  **(**Assessing the learners) | What? The content of the presentation / practice scenarios / feedback ... | PPH management including clinical response times were compared before and after team training using Student’s paired t-test and McNemar’s test. *(abstract)* |
|  | How? The form of the presentation / practice scenarios / feedback ... (e.g., sequencing, scaffolds etc.) | PPH management including clinical response times were compared before and after team training using Student’s paired t-test and McNemar’s test. *(abstract)* All simulations were video recorded and reviewed independently using structured evaluation tools for clinical and team performance by two obstetricians who were not part of the initial study team.The reviewers were blinded to the timing of videos regarding whether they occurred prior to or following team training. *pg 3* |
|  | Medium? The medium used (on paper, lecture, group discussion, simulation etc.) |  |
|  | | |
| **Paper # 13 Maslovitz 2007** | | |
| **Title** | | Recurrent Obstetric Management Mistakes Identified by Simulation |
| **Authors** | | Sharon Maslovitz, Gad Barkai, Joseph B. Lessing, Amitai Ziv, and Ariel Many, |
| **Journal & Publishing information** | | Obstet Gynecol 2007;109:1295–300) |
| **Abstract** | | **OBJECTIVE:** To develop a simulation-based curricular unit for labor and delivery teams involved in obstetric emergencies to detect and address common mistakes. **METHODS:** A simulation-based curricular unit for hands-on training of four obstetric emergency scenarios was developed using high-tech mannequins and low- tech simulators. The scenarios were eclamptic seizure, postpartum hemorrhage, shoulder dystocia, and breech extraction. The obstetric teams consisted of at least one resident and two midwives. Checklists of actions expected from the teams were handed out to the course’s tutors who observed the “event.” All sessions were videotaped and then reviewed and analyzed by the trainees themselves, who were guided by two experienced tutors. We identified the most commonly occurring mistakes by summing up checklists and by watching the recorded sessions. **RESULTS:** Between February 2004 and April 2006, 60 residents in obstetrics and gynecology and 88 midwives underwent the simulation-based course. Forty-two labor and delivery teams completed all four sessions. The most common management errors were delay in transporting the bleeding patient to the operating room (82%), unfamiliarity with prostaglandin administration to reverse uterine atony (82%), poor cardiopulmonary resuscitation techniques (80%), inadequate documentation of shoulder dystocia (80%), delayed administration of blood products to reverse consumption coagulopathy (66%), and inappropriate avoidance of episiotomy in shoulder dystocia and breech extraction (32%). Eighteen trainees were invited for repeated sessions at least 6 months after the first training day, and their scores were significantly higher in the latter sessions (79.4 + 4.3 versus 70 + 5.3 for the second and first simulated eclampsia sessions). **CONCLUSION:** A curricular unit based on simulation of obstetric emergencies can identify pitfalls of management in labor and delivery rooms that need to be addressed. |
| **Presentation**  **(**Information presented to learners) | What? The content of the presentation / practice scenarios / feedback ... | The scenarios were eclamptic seizure, postpartum hemorrhage, shoulder dystocia, and breech extraction. *(abstract)*  The scenarios chosen by most of them were these four: 1) preeclamptic patient with an eclamptic seizure, 2) an intractable postpartum hemorrhage, 3) shoulder dystocia, and 4) breech extraction. *pg 1296* |
|  | How? The form of the presentation / practice scenarios / feedback ... (e.g., sequencing, scaffolds etc.) |  |
|  | Medium? The medium used (on paper, lecture, group discussion, simulation etc.) |  |
| **Practice**  **(**Practice provided to learners) | What? The content of the presentation / practice scenarios / feedback ... |  |
|  | How? The form of the presentation / practice scenarios / feedback ... (e.g., sequencing, scaffolds etc.) | We had to anticipate the actions that would most likely be taken by the trainees and then plan appropriate responses for the mannequin. Consequently, algorithms were created for the two scenarios, and they were fed into the computer attached to the mannequins in such a manner that the simulator acted independently in accordance with the treatment (or lack of it) administrated by the teams. *pg 1296* |
|  | Medium? The medium used (on paper, lecture, group discussion, simulation etc.) | The conditions of eclamptic seizure and postpartum hemorrhage were created for full-scale, high-tech mannequins (Meti, METI Inc, Sarasota, FL; SimMan, Laerdal Medical Ltd, Orpington, Kent, UK). Each basic simulator required adaptation for obstetric requirements, such as a remote control seizure apparatus attached to the bed of the mannequin or a bleeding machine that could be attached to almost every organ, including the vagina (Fig. 1). *pg 1296* |
| **Feedback**  **(**Feedback given to learners) | What? The content of the presentation / practice scenarios / feedback ... |  |
|  | How? The form of the presentation / practice scenarios / feedback ... (e.g., sequencing, scaffolds etc.) | Before each session the trainees were requested to fill out a questionnaire that included inquiries about general data about the trainee (age, gender, institutional affiliation, years of experience), level of experience in managing obstetric emergencies (as a case manager or as an active or passive participant), level of bedside teaching or guidance that the trainee had been exposed to in obstetric emergencies, and a self-estimation of the trainee’s own ability to conduct such a case. *pg 1296*  Feedback questionnaires were handed out to both the trainers and trainees before and after each session to determine the usefulness of and satisfaction from our simulation-based course. Detection of common and recurring mistakes was achieved by reviewing many hours of videotapes and summing up the data in the checklists. *pg 1297* |
|  | Medium? The medium used (on paper, lecture, group discussion, simulation etc.) | All four stations were videotaped from several angles by cameras distributed around the virtual delivery rooms. At the conclusion of each station, the teams viewed the video of their actions along with a senior obstetrician and an experienced midwife who assisted them in analyzing their mistakes and drawing conclusions in preparation for the next session. *pg 1297* |
| **Assessment**  **(**Assessing the learners) | What? The content of the presentation / practice scenarios / feedback ... | Detection of common and recurring mistakes was achieved by reviewing many hours of videotapes and summing up the data in the checklists. *pg 1297* |
|  | How? The form of the presentation / practice scenarios / feedback ... (e.g., sequencing, scaffolds etc.) | Checklists of actions expected from the teams were handed out to the course’s tutors who observed the “event.” *pg 1297* |
|  | Medium? The medium used (on paper, lecture, group discussion, simulation etc.) | Tutors were asked to mark whether or not each action had been done appropriately according to predefined criteria. Each trainee was graded on a scale from 0 to 100 by a scoring system based on the filled out checklists. *pg 1297* |
|  | | |
| **Paper # 14 Maslovitz 2008** | | |
| **Title** | | Improved accuracy of postpartum blood loss estimation as assessed by simulation |
| **Authors** | | SHARON MASLOVITZ, GAD BARKAI, JOSEPH B. LESSING, AMITAI ZIV & ARIEL MANY |
| **Journal & Publishing information** | | Acta Obstetricia et Gynecologica. 2008; 87: 929-934 |
| **Abstract** | | **Objective.** Caregivers underestimate the amount of blood loss, but this almost five decades-old assumption has not been validated. We aimed at assessing the accuracy of estimated blood loss by obstetrical teams during a simulated Postpartum hemorrhage (PPH) scenario. **Study design.** A prospective study conducted as part of the simulation-based training course, using sophisticated mannequin simulators adapted for obstetrical training by specially designed devices. **Setting.** Part of the simulation-based training course. **Population.** Obstetrical teams consisted of physicians and obstetrical nurses. **Methods.** Each of the participating obstetrical teams assessed blood loss during PPH scenarios. Their estimates were compared to the actual predefined 3.5-liter blood loss. An intervention group underwent a similar course in which they recorded their estimations after 1, 2 and 3.5 liters were lost. **Outcome measures.** Blood loss estimates after completion of the scenario in both groups.  **Results.** Fifty obstetrical teams took part in the study. Eight comprised the interventional group. The average estimated blood loss was 1,780 ml (49% underestimation) for non-interventional teams. The interventional groups estimated blood loss to be 2,400 ml (32% underestimation). The main method of estimating blood loss was ‘gut feeling’, followed by verbalized guesses of team members and assessments of the ‘patient’s’ hemodynamic status.  **Conclusions.** Accuracy of blood loss estimations by a simulation-based PPH scenario was 50-60%. Measurements at predetermined intervals significantly improved accuracy of these estimations. Our study suggests that implementation of periodic estimations of blood loss in the management of PPH might improve clinical judgment. |
| **Presentation**  **(**Information presented to learners) | What? The content of the presentation / practice scenarios / feedback ... |  |
|  | How? The form of the presentation / practice scenarios / feedback ... (e.g., sequencing, scaffolds etc.) |  |
|  | Medium? The medium used (on paper, lecture, group discussion, simulation etc.) |  |
| **Practice**  **(**Practice provided to learners) | What? The content of the presentation / practice scenarios / feedback ... |  |
|  | How? The form of the presentation / practice scenarios / feedback ... (e.g., sequencing, scaffolds etc.) | The setup for the study was a simulation-based hands-on training course that had been in operation for three years. The training day was divided into four learning units including a station dedicated to the management of obstetrical emergencies which was based on two scenarios (eclamptic seizures and PPH) using sophisticated mannequins (Meti† - † METI Inc., Sarasota, Florida, USA; Simman 􏰕 Laerdal, Norway).  The panel of tutors consisted of six senior perinatologists and four experienced midwives who had previously attended guidance sessions for trainers. We had to anticipate the actions that would most likely be taken by the trainees and then plan appropriate responses by the mannequin. For that purpose, algorithms were created (12) and fed into the computer attached to the mannequin in such a manner that the simulator responded in accordance with the treatment (or lack of it) administered by the teams (Figure 1). The PPH scenario was designed to assess the ability of obstetrical teams to detect and treat this extreme complication of an otherwise normal delivery in a decisive and appropriate fashion. The team members were expected to deal with an intractable PPH apparently caused by uterine atony, which was only partially and temporarily responding to powerful uterotonics (Figure 2). *pg 930* |
|  | Medium? The medium used (on paper, lecture, group discussion, simulation etc.) | These mannequins were equipped with many replicas of human organs and were attached to computers capable of being pro- grammed in advanced. Since the sophisticated simulators were not originally developed as obstetrical mannequins, we had to adapt and adjust them for our purposes. For instance, we developed a special bleeding device that was made of a commercial spray container attached on one end to an air plug on the wall (in order to create pressure) and on the other end to a tube ending at the simulated mannequin’s vagina. The amount and intensity of bleeding were controlled by a remote control device operated by a trained tutor. The mannequin ‘bled’ a total of exactly 3.5 liters during each session. The mannequin, ‘Mary’, can communicate through a microphone implanted in her larynx and activated by the mannequin operator. A variety of routinely used hemodynamic parameters (pulse, blood pressure, pulse oxymetry, urinary output through a catheter inserted into the mannequin’s urethra) are available to the teams. The hemodynamic status is coordinated with the amount of bleeding and alters automatically according to the actions taken by the teams. The teams can also use other hospital facilities, such as the operating room and blood bank, and they are expected to administer blood products at the appearance of signs of hypovolemic shock. *pg 930/931* |
| **Feedback**  **(**Feedback given to learners) | What? The content of the presentation / practice scenarios / feedback ... | The items *(of the oral feedback quesitonnaire)* were: How much blood do you estimate that the ‘patient’ lost?  Which factors did you consider for estimating the blood loss?  Did you arrive at a figure larger than your actual estimation knowing that physicians typically underestimate in such situations?  Have you estimated the amount of blood loss during the ‘acute event’? Would you have managed the case completely differently had you known the true extent of bleeding? What do you consider the cutoff point of blood loss for urgent/intense treatment? *pg 931* |
|  | How? The form of the presentation / practice scenarios / feedback ... (e.g., sequencing, scaffolds etc.) | All sessions of the PPH scenarios were videotaped from several angles by cameras distributed around the virtual delivery rooms. At the conclusion of each session, the teams viewed the video of their actions along with a senior obstetrician and an experienced midwife who assisted them in analyzing their mis- takes and in drawing conclusions. During the discussion that followed, an oral questionnaire was presented to the resident who had been assigned as the case manager as well as to the other team members. *pg 931* |
|  | Medium? The medium used (on paper, lecture, group discussion, simulation etc.) |  |
| **Assessment**  **(**Assessing the learners) | What? The content of the presentation / practice scenarios / feedback ... | The tutors were requested to mark whether or not each action had been carried out appropriately according to pre-defined criteria. *pg 931* |
|  | How? The form of the presentation / practice scenarios / feedback ... (e.g., sequencing, scaffolds etc.) | Checklists that included a comprehensive list of actions that were required of the teams during each stage were handed out to the tutors who attended the ‘event’. Each trainee was graded on a scale from 0 to 100. *pg 931* |
|  | Medium? The medium used (on paper, lecture, group discussion, simulation etc.) |  |

1. **Articles' Tables PPH Simulation & ID Subset15to21**

| **Paper # 15 Nelissen** | | |
| --- | --- | --- |
| **Title** | | Helping mothers survive bleeding after birth: an evaluation of simulation-based training in a low-resource setting. |
| **Authors** | | [Nelissen E](http://www.ncbi.nlm.nih.gov/pubmed/?term=Nelissen%20E%5BAuthor%5D&cauthor=true&cauthor_uid=24344822)^1^, [Ersdal H](http://www.ncbi.nlm.nih.gov/pubmed/?term=Ersdal%20H%5BAuthor%5D&cauthor=true&cauthor_uid=24344822), [Ostergaard D](http://www.ncbi.nlm.nih.gov/pubmed/?term=Ostergaard%20D%5BAuthor%5D&cauthor=true&cauthor_uid=24344822), [Mduma E](http://www.ncbi.nlm.nih.gov/pubmed/?term=Mduma%20E%5BAuthor%5D&cauthor=true&cauthor_uid=24344822), [Broerse J](http://www.ncbi.nlm.nih.gov/pubmed/?term=Broerse%20J%5BAuthor%5D&cauthor=true&cauthor_uid=24344822), [Evjen-Olsen B](http://www.ncbi.nlm.nih.gov/pubmed/?term=Evjen-Olsen%20B%5BAuthor%5D&cauthor=true&cauthor_uid=24344822), [van Roosmalen J](http://www.ncbi.nlm.nih.gov/pubmed/?term=van%20Roosmalen%20J%5BAuthor%5D&cauthor=true&cauthor_uid=24344822), [Stekelenburg J](http://www.ncbi.nlm.nih.gov/pubmed/?term=Stekelenburg%20J%5BAuthor%5D&cauthor=true&cauthor_uid=24344822). |
| **Journal & Publishing information** | | [Acta Obstet Gynecol Scand.](http://www.ncbi.nlm.nih.gov/pubmed/24344822) 2014 Mar;93(3):287-95. doi: 10.1111/aogs.12321. Epub 2014 Jan 15. |
| **Abstract** | | **Objective.** To evaluate “Helping Mothers Survive Bleeding After Birth” (HMS BAB) simulation-based training in a low-resource setting.  **Design.** Educational intervention study.  **Setting.** Rural referral hospital in Northern Tanzania.  **Population.** Clinicians, nurse-midwives, medical attendants, and ambulance drivers involved in maternity care.  **Methods.** In March 2012, health care workers were trained in HMS BAB, a half-day simulation-based training, using a train- the-trainer model. The training focused on basic delivery care, active management of third stage of labor, and treatment of postpartum hemorrhage, including bimanual uterine compression.  **Main outcome measures.** Evaluation questionnaires provided information on course perception. Knowledge, skills, and confidence of facilitators and learners were tested before and after training. **Results.** Four master trainers trained eight local facilitators, who subsequently trained 89 learners. Evaluation revealed that HMS BAB training was considered acceptable and feasible, although more time should be allocated for training, and teaching materials should be translated into the local language. Knowledge, skills, and confidence of learners increased significantly immediately after training. However, overall pass rates for skills tests of learners after training were low (3% pass rate for basic delivery and management of postpartum hemorrhage).  **Conclusions.** The HMS BAB simulation-based training has potential to contribute to education of health care providers. We recommend a full day of training and validation of the facilitators to improve the training.  Abbreviations: AMTSL, active management of third stage of labor; BAB, Bleeding After Birth; HBB, Helping Babies Breathe; HMS, Helping Mothers Survive; PPH, postpartum hemorrhage. |
| **Presentation**  **(**Information presented to learners) | What? The content of the presentation / practice scenarios / feedback ... | The training focused on basic delivery care, active management of third stage of labor, and treatment of postpartum hemorrhage, including bimanual uterine compression. *(abstract)*  The training materials were reviewed by external stakeholders from different international organizations. The first module, “Bleeding After Birth” (BAB), focuses on basic delivery care, active management of third stage of labor (AMTSL), and treatment of PPH (12). The training is designed for all levels of health care providers. Training started with an introduction regarding the development and aim of the HMS BAB training (1 h). This was followed by 3 h of theory regarding basic delivery care, active management of third stage of labor, and treatment of PPH, using training materials such as the “Action Plan” (wall poster to aid decision making), a “Training Flip Book” (graphic display used during training), and a “Facilitation Guide” (in English). *pg 288* |
|  | How? The form of the presentation / practice scenarios / feedback ... (e.g., sequencing, scaffolds etc.) | An introduction of the course by one of the master trainers followed and subsequently the learners were allocated to the four available facilitators. Training was conducted in small groups and the groups contained health care providers of different cadres (maximum six learners per facilitator). It started with 1.5 h of theory using the HMS BAB training materials... *pg 288* |
|  | Medium? The medium used (on paper, lecture, group discussion, simulation etc.) | Training started with an introduction regarding the development and aim of the HMS BAB training (1 h). This was followed by 3 h of theory regarding basic delivery care, active management of third stage of labor, and treatment of PPH, using training materials such as the “Action Plan” (wall poster to aid decision making), a “Training Flip Book” (graphic display used during training), and a “Facilitation Guide” (in English). *pg 288* |
| **Practice**  **(**Practice provided to learners) | What? The content of the presentation / practice scenarios / feedback ... | An educational intervention study was carried out in March 2012 at Haydom Lutheran Hospital, a rural referral hospital in Northern Tanzania. *pg 288* |
|  | How? The form of the presentation / practice scenarios / feedback ... (e.g., sequencing, scaffolds etc.) | HMS makes use of a train-the-trainer model in which training is cascaded down from master trainers to local facilitators to learners. Subsequently, local facilitators trained local learners in a half-day session under supervision of master trainers. In total there were six training sessions, divided over three days. Clinicians, nurse-midwives, medical attendants (nurse aids without formal medical education), and ambulance drivers (without formal medical education) involved in maternity care (including nurse-midwives from the intensive care unit and operating theatre) were selected by the hospital management to attend training. Training took place in a communal room in the hospital and started with a simulated scenario of a birth complicated by PPH and maternal death with a facilitator taking on the role of a patient actor. Training was conducted in small groups and the groups contained health care providers of different cadres (maximum six learners per facilitator). It started with 1.5 h of theory using the HMS BAB training materials, and was followed by 1.5 h of skills and scenario training with the birthing simulator. *pg 288* |
|  | Medium? The medium used (on paper, lecture, group discussion, simulation etc.) | In the second half of the day, MamaNatalie (Laerdal Global Health, Stavanger, Norway), a low-cost, low-tech birthing simulator was introduced. The different components of AMTSL and treatment of PPH were addressed by using the mannequin. Each facilitator took part in scenario training and received feedback from the master trainer. *pg 288* |
| **Feedback**  **(**Feedback given to learners) | What? The content of the presentation / practice scenarios / feedback ... | Confidence was appraised using a questionnaire, which was filled in by all facilitators and learners immediately before and after training. Five answers were possible ranging from 1 = I cannot perform this skill to 5 = extremely confident. This questionnaire also contained questions about their characteristics. *pg 290* |
|  | How? The form of the presentation / practice scenarios / feedback ... (e.g., sequencing, scaffolds etc.) | Each facilitator took part in scenario training and received feedback from the master trainer. Each learner took part in one scenario and received feedback from the facilitator. *pg 288* |
|  | Medium? The medium used (on paper, lecture, group discussion, simulation etc.) |  |
| **Assessment**  **(**Assessing the learners) | What? The content of the presentation / practice scenarios / feedback ... | Knowledge, skills, and confidence of facilitators and learners were tested before and after training. *(abstract)*  First, competences required for PPH prevention and management were selected and linked to a measurable item.  Due to logistic reasons, only health care providers working in labor ward, ambulance drivers, and facilitators were enrolled for skills assessment (Figure 2). They were tested in the week before training, and again in the week after training. Performance was assessed in two simulated scenarios using the birthing simulator: “basic delivery” and “management of PPH due to uterine atony”. The second scenario was further divided into “management of PPH” and “performance of bimanual uterine compression”. *pg 289* |
|  | How? The form of the presentation / practice scenarios / feedback ... (e.g., sequencing, scaffolds etc.) | Evaluation questionnaires provided information on course perception.  The intervention was assessed according to level 1 and 2 of the Kirkpatrick model, a method commonly used for evaluation of training programs (Figure 1) (16). To assess course perception (level 1), both facilitators and learners were requested to fill out an evaluation questionnaire immediately after training to record their opinion about the feasibility and acceptability of HMS BAB training. A 5-point Likert scale was used (ranging from 1 = strongly disagree to 5 = strongly agree). In addition, suggestions to improve training could be made in an open remark. A draft evaluation questionnaire had previously been reviewed and approved by maternal health experts in a group meeting.  Level 2 of the Kirkpatrick model relates to knowledge, skills, and attitudes acquired due to training. Knowledge was assessed by means of a written 26-item knowledge test that was administered immediately before and after training. It consisted of 14 multiple-choice questions and 12 yes/no questions. The criterion-referenced pass score was ≥70% correct answers. The knowledge test was developed and validated by Jhpiego.  A draft checklist based on literature and clinical experience was created, and six maternal health experts reviewed the draft checklist. *pg 289* |
|  | Medium? The medium used (on paper, lecture, group discussion, simulation etc.) | Before starting the skills test, MamaNatalie was introduced to the facilitators and learners to familiarize them with the mannequin. Furthermore, the concept of simulation was explained, along with a description of what was to be expected from the health care worker. A scenario description was given prior to the start of each scenario.  The skills test was videotaped and subsequently assessed by two independent assessors (both residents in obstetrics and gynecology in the Netherlands). The assessors were blinded for the time of testing (pre- or post- intervention).  Attitude was addressed by assessing confidence of facilitators and learners in their ability to perform AMTSL, manage PPH, determine completeness of the placenta, perform bimanual uterine compression, and access advanced care. *pg 290* |
|  | | |
| **Paper # 16 Philippi** | | |
| **Title** | | Interprofessional simulation of a retained placenta and postpartum hemorrhage. |
| **Authors** | | [Phillippi JC](http://www.ncbi.nlm.nih.gov/pubmed/?term=Phillippi%20JC%5BAuthor%5D&cauthor=true&cauthor_uid=25707310), [Buxton M](http://www.ncbi.nlm.nih.gov/pubmed/?term=Buxton%20M%5BAuthor%5D&cauthor=true&cauthor_uid=25707310), [Overstreet M](http://www.ncbi.nlm.nih.gov/pubmed/?term=Overstreet%20M%5BAuthor%5D&cauthor=true&cauthor_uid=25707310). |
| **Journal & Publishing information** | | Nurse Educ Pract. 2015 Feb 14. pii: S1471-5953(15)00028-1. doi: 10.1016/j.nepr.2015.02.001. [Epub ahead of print] |
| **Abstract** | | To improve quality and safety in healthcare, national and international organizations have called for students to receive dedicated training in interprofessional communication and collaboration. We developed a simulation for nurse-midwifery and nurse-anesthesia students, using the Core Competencies for Interprofessional Collaborative Practice framework. The simulation, involving a postpartum women with a retained placenta and acute blood loss, allowed students to collaboratively manage a high-risk situation. We present the details of the simulation and evaluation to assist educators.  **Keywords:** Simulation Interprofessional Interdisciplinary Communication Competency-based education Collaboration |
| **Presentation**  **(**Information presented to learners) | What? The content of the presentation / practice scenarios / feedback ... | The student learning goals were derived from the Core Competencies for Interprofessional Collaborative Practice and included: 1) understanding and articulating the roles and responsibilities of all healthcare team members, 2) effectively communicating pertinent information to other members of the healthcare team, and 3) sharing leadership and responsibility with other healthcare team members to appropriately manage the patient needs. *pg 2* |
|  | How? The form of the presentation / practice scenarios / feedback ... (e.g., sequencing, scaffolds etc.) |  |
|  | Medium? The medium used (on paper, lecture, group discussion, simulation etc.) | A post-partum hemorrhage and retained placenta was the patient crisis for the event as it required implementation of complex knowledge and collaboration at the peak of their practice capacity for both groups of students. While manual removal of the placenta is a rare event for nurse-midwives in the US, it is an essential competency for use in emergency situations. The case was designed so that there was not another method of managing the rapidly unfolding hemorrhage; if students requested physician consultation they were informed the physician was unavailable due to an emergency, forcing them to manage the patient situation. *pg 2* |
| **Practice**  **(**Practice provided to learners) | What? The content of the presentation / practice scenarios / feedback ... | While waiting outside the simulation room, students were given the patient information: You are at a rural hospital. Your midwife colleague is in the other room with an emergency, and the consulting obstetrician is at home. The on-call nurse-anesthetist is in the call room. You are asked to help with this patient who is feeling strong pressure. G2P1 woman was admitted 4 h ago in active labor and received an epidural, reporting high levels of pain for the past half hour. All prenatal was normal, no complications in labor or with her previous birth. Continuous fetal monitoring has been Category I since admission. Estimated fetal weight is 7.5 pounds. *pg 4* |
|  | How? The form of the presentation / practice scenarios / feedback ... (e.g., sequencing, scaffolds etc.) | Once the students had the opportunity to review the background information, a student acting as a nurse came out into the hallway to ask for assistance for the laboring patient. As the students arrived at the simulation site, they were assigned a role for the simulation as shown in Table 2. Nursing students, if available, were assigned to be nurses. Nurse-midwifery students were assigned one of five roles: lead midwife, preceptor midwife, nurse, family member, or booth observer. Student nurse- anesthetists were assigned to be the lead nurse-anesthetist, the preceptor nurse-anesthetist, or a booth observer. This layered approach allowed the lead students to ask for help and provided multiple viewpoints of the event. In addition, it allowed a greater number of students to participant in each simulation. After the students were assigned their role, they were led into the simulation room and control booth to orient to the setting. The anesthesia school hosted the simulation as it had a large mock operating room with a control booth where several faculty and students could observe the simulation and control the simulated patient's vital signs. The students were shown where to find commonly needed supplies and how to work all the equipment. Nurse-midwifery students were oriented to the PartoPants, including the location of the vaginal introitus, urethra, and rectum. Students were asked to introduce themselves to the whole group, including their name and role within the simulation. Following introductions, students who would act as practitioners were lead out of the room and students acting as a nurse, family member, or booth observer were asked to take their places. *pg 3* |
|  | Medium? The medium used (on paper, lecture, group discussion, simulation etc.) | The simulation was designed to include a birth followed by a retained placenta and a severe postpartum hemorrhage. This scenario required nursing, nurse-midwifery, and nurse-anesthesia students to communicate and collaborate to manage the unfolding patient crisis. Nurse-midwifery students were expected to remove the placenta and manage a postpartum hemorrhage in addition to clearly communicating with all team members, including the mother, about the need for pain control prior to the procedure, and the ongoing amount of blood loss. The nurse-anesthesia student was expected to administer appropriate medications for pain control and perform fluid management while effectively communicating with team members and the patient and family. The nursing student was expected to implement orders, prompt the practitioners as appropriate, care for the newborn baby, and communicate with all team members. To encourage the students to learn to interact with team members and patients during an emergency, we chose to have a person, rather than a high-fidelity mannequin, as the patient. To encourage realism, the simulated patient wore PartoPantsTM to allow students to assist the birth and provide bimanual compression while interacting with her (Cohen et al., 2011). The SimManTM monitor display was used for vital signs to allow for remote manipulation from the control room.  Simulation preparation  The simulated patient was advised to wear tight leggings and several pairs of padded undergarments were placed over the leggings to provide a padded buffer between her genitals and the students' hands. The PartoPants were worn over the undergarments and fit loosely over the simulated patient's legs and up to her abdomen, allowing her room for manipulation of the baby, uterus, and placenta. After she was dressed in the PartoPants and positioned on the birthing bed, an IV was taped to her right arm and connected to a bag of lactated-ringers solution. The simulated patient kept her left arm under a sheet to control the baby doll and uterus. An IV-task trainer model was placed at her left shoulder to allow students to place a second IV if needed. In addition, a labeledepidural catheter was taped to her shoulder and open to drain onto her gown to provide a signal that the epidural had been re-dosed. A grapefruit was positioned in the PartoPants along the simulated patient's left leg for the patient to move to her abdomen when the students had successfully managed the patient bleeding; the grapefruit simulated a firm uterus. Bags of red fluid with tubing leading to the PartoPants introitus were placed so a concealed faculty member could control the blood loss. An earpiece speaker allowed the simulated patient to hear commands from the control booth. Immediately prior to the simulation, the patient loaded the baby doll and placenta into the uterus, and positioned them on her abdomen close to the vaginal introits. Faculty then arranged the tubing carrying the red fluid to drain onto chux pads between the patient's legs to simulate vaginal blood loss.  The anesthesia students were called to the room by the nurse following the nurse-midwifery student's request for an anesthesia consult. Anesthesia students needed to enter the room and quickly communicate their presence to the patient, nursing students, and midwifery students, then rapidly assess the patient's vital signs, pain, and sensory level. After receiving report from the acting midwife, the lead anesthesia student needed to re-dose the epidural and/or administer a short-acting IV anesthetic, as well as communicate these actions with the larger healthcare team.  The midwifery student was expected to enter the room, quickly assess the patient (who was close to giving birth), and then gown and glove. Within 3 min a vigorous baby was born. (The APGAR score was taped to the back of the doll.) Following the birth of the baby, there was brisk vaginal bleeding, but the placenta would not deliver with cord traction. In order to decrease the bleeding, the midwifery student needed to manually remove the placenta. If the student failed to act, the bleeding would increase, and the standardized patient would report feeling faint. However, if the student began to remove the placenta, the standardized patient would scream and move away from the midwife. To properly manage the scenario, the midwifery student needed to call the anesthesia team to assist with pain control.  Once the placenta was manually removed, the bleeding would increase, and the uterus (knit uterus) would feel soft and floppy, consistent with uterine atony. The lead midwife was expected to use bimanual compression to control the bleeding and simulta- neously order uterotonics. In addition, he/she needed to communicate with the nursing and anesthesia students about blood loss. Anesthesia students needed to monitor vital signs, manage hemodynamics, and communicate with the midwifery students and request a second IV if appropriate. The scenario included continual deterioration of the maternal condition until participating students effectively communicated and implemented evidence-based care. When effectively managed, the patient would improve: bleeding would decrease, the uterus would become firm (the simulated patient would replace the knit uterus with a grapefruit), and vital signs would stabilize. If the students failed to achieve the objectives and the patient status was grave, the faculty would enter the simulation and provide performance prompts. The simulation was continued until the situation had been successfully resolved. *pg 3/4* |
| **Feedback**  **(**Feedback given to learners) | What? The content of the presentation / practice scenarios / feedback ... | Following this discussion of emotions, students were asked to discuss the simulation sequence of events and their decision- making and management. Lead practitioners were asked to speak first and then other students were asked to share their opinions. After initial comments, booth observers were asked to comment on the ‘whole’ of the simulation. If students were unable to articulate key events, video replay was used to assist discussion. The discussion of patient management lead easily into a conversation about communication and collaboration. Comments were remarkably similar across simulation groups for both cohorts. *pg 4* At the end of the debriefing, students were asked how the simulation would affect their future practice. Faculty closed the debriefing with a review of the simulation objective and students were asked if they had achieved the goal of increasing their ability to effectively communicate and collaborate with other professionals. Following student discussion of the topic, faculty listed ways the group had met the objective. Even if the students had not provided ideal management, each student met the goal of increasing their skills in interprofessional communication and collaboration. Students were then asked for comments about the simulation, including what facets were helpful to their learning and what needed to be improved. *pg 5* |
|  | How? The form of the presentation / practice scenarios / feedback ... (e.g., sequencing, scaffolds etc.) | Immediately following the simulation, students were led to an adjacent conference room for debriefing. The Ee-chats method (Overstreet, 2010) was used to guide the debriefing process. While one faculty member took the lead in facilitation, all simulation faculty were present and involved in the discussion. To begin the debriefing, students were encouraged to talk about their emotions during the simulation to validate their experience and understand how emotions may have affected perfomance. The students in the lead and preceptor roles were asked to speak first, followed by students acting as the nurse and family member. The simulated patient then discussed her emotional experience of the simulation. Booth observers were also encouraged to share their observations. *pg 4* |
|  | Medium? The medium used (on paper, lecture, group discussion, simulation etc.) |  |
| **Assessment**  **(**Assessing the learners) | What? The content of the presentation / practice scenarios / feedback ... |  |
|  | How? The form of the presentation / practice scenarios / feedback ... (e.g., sequencing, scaffolds etc.) | Student evaluation of the simulation occurred in two phases: immediatelly following the simulation during the debriefing and weeks after the simulation via course evaluation or confidential online survey.  In order to elicit the student perspective of the simulation and obtain feedback for improvement, all students participating in the 2013 simulation were sent an online survey two weeks following the event. The anonymous survey included five questions asking for free-text answers and a free-text box for additional comments. *pg 5* |
|  | Medium? The medium used (on paper, lecture, group discussion, simulation etc.) |  |
|  | | |
| **Paper # 17 Roberstson** | | |
| **Title** | | Simulation-Based Crisis Team Training for Multidisciplinary Obstetric Providers |
| **Authors** | | Bethany Robertson; Lori Schumacher; Gabriella Gosman; Ruth Kanfer; Maureen Kelley, P; Michael DeVita. |
| **Journal & Publishing information** | | Simulation in Healthcare Vol. 4, No. 2, Summer 2009 DOI: 10.1097/SIH.0b013e31819171cd |
| **Abstract** | | **Background:** The use of team training programs is promising with regards to their ability to impact knowledge, attitudes, and behavior about team skills. The purpose of this study was to evaluate a simulation-based team training program called Obstetric Crisis Team Training Program (OBCTT) (based on the original training program of Crisis Team Training) framed within a multilevel team theoretical model. We hypothesized that participation in OBCTT would positively impact 10 variables: individual’s knowledge (about team process and obstetric emergency care); confidence and competence in handling obstetric emergencies; and participant attitudes (toward the utility of a rapid response team, simulation technology as a teaching methodology, the utility of team skills in the workplace, comfort in assuming team roles; and individual and team performance). Improvement of objectively measured team performance in a simulated environment was also assessed.  **Methods:** Twenty-two perinatal health care professionals (attending physicians, nurses, resident, and nurse midwives) volunteered to participate in this pretest-posttest study design. All participants were given an online module to study before attending a 4-hour training session. Training consisted of participation in four standardized, simulated crisis scenarios with a female birthing simulator mannequin. Team simulations were video recorded. Debriefings were conducted after each simulation by having team members review the video and discuss team behaviors and member skills. Self-report measures of perinatal and team knowledge as well as several attitude surveys were given at the beginning and again at the end of the training session. A postsimulation attitude survey was administered immediately after the first and last simulation, and a course reaction survey was administered at the end of the training program. Objective task completion scores were computed after each simulation to assess performance.  **Results:** There were significant (*P* < 0.004) improvements in three of the outcome variables, after controlling for type I error with Bonferroni’s correction; attitudes toward competence in handling obstetric emergencies (*t* = 1.6), as well as individual (*t* = 4.2), and team performance (*t* = 4.1). The remaining 6 variables, attitude toward simulation technology, attitude toward the rapid response team; confidence in handling obstetric emergencies; utility of team skills in the workplace; comfort in assuming various team roles; and knowledge, were not statistically significant. Overall task completion from the first to the last simulation (X2F, *df* = 3, n = 3, 8.2, *P* = 0.042) substantially improved (*P* < 0.05).  **Conclusion:** The crisis team training model is applicable to obstetric emergencies. Trainees exhibit a positive change in attitude; perception of individual and team performance, and overall team performance in a simulated environment. The ability of individuals to accurately assess their performance improved as a result of training. |
| **Presentation**  **(**Information presented to learners) | What? The content of the presentation / practice scenarios / feedback ... | ... crisis resource management principles, crisis team skills, team choreography, including a breakdown of team roles and responsibilities for crisis responders, and obstetric emergency management content. *pg 78* |
|  | How? The form of the presentation / practice scenarios / feedback ... (e.g., sequencing, scaffolds etc.) | All participants were given an online module to study before attending a 4-hour training session. *(abstract)* The training program consists of the following steps. (1) Participants review online material before the simulation experience. These materials consist of a slide presentation focusing on crisis resource management principles, crisis team skills, team choreography, including a breakdown of team roles and responsibilities for crisis responders, and obstetric emergency management content. Before coming to the training, participants completed the online didactic training and precourse surveys. *pg 78* |
|  | Medium? The medium used (on paper, lecture, group discussion, simulation etc.) |  |
| **Practice**  **(**Practice provided to learners) | What? The content of the presentation / practice scenarios / feedback ... |  |
|  | How? The form of the presentation / practice scenarios / feedback ... (e.g., sequencing, scaffolds etc.) | Training consisted of participation in four standardized, simulated crisis scenarios with a female birthing simulator mannequin. Team simulations were video recorded. We used a quasi-experimental, pretest-posttest design to evaluate CTTs effectiveness with obstetric health care professionals (Obstetric Crisis Team Training—OBCTT). ... (2) On the day of the simulation course, the facilitator begins with a brief didactic slide presentation reviewing core material and orienting participants to the course. (3) The facilitator orients the participants to the simulated labor and delivery room, manikin, and scenario procedure. (4) Participants perform 4 simulated obstetric crisis scenarios lasting approximately 5 minutes each: fetal bradycardia, anaphylactic shock, shoulder dystocia, and postpartum hemorrhage. Each team performs these in the same order. Participants self-assign a different role during each scenario exercise, and are encouraged not to play the same role twice. The scenarios are short with limited medical challenges to facilitate focus on organization and teamwork. (5) A study assistant video records each scenario. *pg 78* |
|  | Medium? The medium used (on paper, lecture, group discussion, simulation etc.) | Simulation training occurred in a vacant operating suite at the hospital; one room simulated a labor and delivery room and was used to enact the scenarios. Three training groups completed the program. Each group included at least one person from each of the following disciplines: perinatal nurse, obstetric physician, and certified nurse midwife. One obstetrics and gynecology resident participated in training. Total training session time for each group was between 3 and 4 hours and the training format for each group was identical. *pg 78* |
| **Feedback**  **(**Feedback given to learners) | What? The content of the presentation / practice scenarios / feedback ... | Then the facilitator displays, using a laptop and LCD projector, the Team Task Completion Checklist, a checklist of the choreographed roles and responsibilities (see Fig. 1). *pg 79* The facilitator begins the debriefing by asking if all the roles were filled and if so, who filled them and were the corresponding tasks completed; yes/no/nonapplicable (eg, “Did the bedside assistant stay by the patient?”). Participants come to consensus and the facilitator records their response, which provides an immediate visual performance feedback as the spreadsheet generates a graphic of the task completion score after every scenario.The standardization of the debriefing experience around the topics of role assumption, task completion, communication and organization provides participants with a consistent focus from training to training. After the first simulation, the debriefing emphasizes the lack of coordination and the need for organization by assuming roles within the team. The second debriefing reviews role assumption and focuses on completing tasks required by each role. The third debriefing emphasizes how communication can facilitate the team’s performance. The fourth debriefing pushes participants to link material learned in the course to “real world” experiences. *pg 78* |
|  | How? The form of the presentation / practice scenarios / feedback ... (e.g., sequencing, scaffolds etc.) | Debriefings were conducted after each simulation by having team members review the video and discuss team behaviors and member skills. Self-report measures of perinatal and team knowledge as well as several attitude surveys were given at the beginning and again at the end of the training session. A postsimulation attitude survey was administered immediately after the first and last simulation, and a course reaction survey was administered at the end of the training program.  After each scenario; the facilitator leads a 30-minute video-based structured debriefing. As a group, participants first watch the video of the simulation. The facilitator uses this data, in conjunction with a different standardized debriefing tool after each scenario, to guide the reflection of the team process. *pg 78* |
|  | Medium? The medium used (on paper, lecture, group discussion, simulation etc.) |  |
| **Assessment**  **(**Assessing the learners) | What? The content of the presentation / practice scenarios / feedback ... | Objectively measured team performance in a simulated environment was also assessed. *pg 78*  Course Measures *Precourse*: we collected the following: (1) demographics (gender, age, years experience, professional position, and ethnicity); (2) experience with obstetric emergencies; (3) experience with simulation; (4) attitudes toward group work (collectivism/conflict management); and (5) attitudes toward performance appraisal. *Precourse and postcourse*: we surveyed participants about perceived confidence and competence in responding to obstetric emergencies. The questionnaire also assessed attitudes toward the utility of a rapid response system, teamwork in healthcare, and simulation-based training. Perceptions of individual and team performance were also measured after the first and final simulation. Participants took a 15-item multiple choice knowledge test that measured 2 domains: obstetric emergency care and team roles. Postcourse only, we evaluated participant satisfaction with the training program. *pg 79* |
|  | How? The form of the presentation / practice scenarios / feedback ... (e.g., sequencing, scaffolds etc.) | Precourse:All surveys, except the demographics, used a 5-point Likert or ordinal scale. *Precourse and postcourse*: All responses were assessed using a 5-point Likert scale (1 = “strongly disagree,” 5 = “strongly agree”). *During the course*: immediately after the first simulation and before viewing the video-tape recording, participants completed a survey assessing individual and team perfor- mance. This was repeated after the fourth simulation as well. The Team Task Completion Checklist was used to score role assumption and task completion for each scenario. The instrument was adapted from the WISER CTT course. Obstetric, CTT, and simulation education content experts at the University of Pittsburgh modified the original instrument to reflect the specific structure and needs of obstetric crisis response.  This adapted instrument was further modified to reflect the obstetric crisis response team structure of the study site.  Task completion (yes, no, not applicable) was scored based on a consensus opinion of participants and facilitator. The tasks were then tallied to obtain an overall task completion rate for each scenario. *pg 81* |
|  | Medium? The medium used (on paper, lecture, group discussion, simulation etc.) |  |
|  | | |
| **Paper # 18 Crofts** | | |
| **Title** | | Change in knowledge of midwives and obstetricians following obstetric emergency training: a randomised controlled trial of local hospital, simulation centre and teamwork training |
| **Authors** | | JF Crofts, D Ellis, TJ Draycott, C Winter, LP Hunt, VA Akande |
| **Journal & Publishing information** | | Crofts J, Ellis D, Draycott T, Winter C, Hunt L, Akande V. Change in knowledge of midwives and obstetricians following obstetric emergency training: a randomised controlled trial of local hospital, simulation centre and teamwork training. BJOG 2007;114:1534–1541. |
| **Abstract** | | Objectives To explore the effect of obstetric emergency training on knowledge. Furthermore, to assess if acquisition of knowledge is influenced by the training setting or teamwork training.  Design A prospective randomised controlled trial. Setting Training was completed in six hospitals in the South West of England, UK and at the Bristol Medical Simulation Centre, UK.  Population Midwives and obstetric doctors working for the participating hospitals were eligible for inclusion in the study. A total of 140 participants (22 junior and 23 senior doctors, 47 junior and 48 senior midwives) were studied.  Methods Participants were randomised to one of four obstetric emergency training interventions: (1) 1-day course at local hospital, (2) 1-day course at simulation centre, (3) 2-day course with teamwork training at local hospital and (4) 2-day course with teamwork training at simulation centre.  Main outcome measures Change in knowledge was assessed by a 185 question Multiple-Choice Questionnaire (MCQ) completed up to 3 weeks before and 3 weeks after the training intervention.  Results There was a significant increase in knowledge following training; mean MCQ score increased by 20.6 points (95% CI 18.1–23.1, P < 0.001). Overall, 123/133 (92.5%) participants increased their MCQ score. There was no significant effect on the MCQ score of either the location of training (two-way analysis of variants P = 0.785) or the inclusion of teamwork training (P = 0.965).  Conclusions Practical, multiprofessional, obstetric emergency training increased midwives’ and doctors’ knowledge of obstetric emergency management. Furthermore, neither the location of training, in a simulation centre or in local hospitals, nor the inclusion of teamwork training made any significant difference to the acquisition of knowledge in obstetric emergencies.  Keywords Knowledge, obstetric emergencies, simulation, team- work, training. |
| **Presentation**  **(**Information presented to learners) | What? The content of the presentation / practice scenarios / feedback ... |  |
|  | How? The form of the presentation / practice scenarios / feedback ... (e.g., sequencing, scaffolds etc.) | The clinical content of both the 1- and 2-day training interventions (courses) was identical regardless of the locality. Lectures were given using standardised PowerPoint presentations and lecture notes. |
|  | Medium? The medium used (on paper, lecture, group discussion, simulation etc.) |  |
| **Practice**  **(**Practice provided to learners) | What? The content of the presentation / practice scenarios / feedback ... | Simulated clinical emergency scenarios, ‘fire-drills’, followed the same outline in the local and simulation centre courses; however, the training equipment used at the simulation centre was more sophisticated, using computer-controlled mannequins that respond to treatment, than that used in the local courses. .. Participants were randomised to one of four obstetric emergency training interventions: (1) 1-day course at local hospital, (2) 1-day course at simulation centre, (3) 2-day course with teamwork training at local hospital and (4) 2-day course with teamwork training at simulation centre. The description of the different training interventions is summarised in Table 1. *pg 1535*  The clinical content of both the 1- and 2-day training interventions (courses) was identical regardless of the locality. |
|  | How? The form of the presentation / practice scenarios / feedback ... (e.g., sequencing, scaffolds etc.) | ‘Fire-drills’ were conducted on the delivery suite in each local hospital or in a simulated clinical environment at the simulation centre. All participants received a course manual; the manual for the 2-day course included an additional chapter on team working. Details of training on Table 1. See table1 from the article *pg 1536* |
|  | Medium? The medium used (on paper, lecture, group discussion, simulation etc.) | Simulated clinical emergency scenarios, ‘fire-drills’, followed the same outline in the local and simulation centre courses; however, the training equipment used at the simulation centre was more sophisticated, using computer-controlled mannequins that respond to treatment, than that used in the local courses. .. Participants were randomised to one of four obstetric emergency training interventions: (1) 1-day course at local hospital, (2) 1-day course at simulation centre, (3) 2-day course with teamwork training at local hospital and (4) 2-day course with teamwork training at simulation centre. The description of the different training interventions is summarised in Table 1. *pg 1535*  The clinical content of both the 1- and 2-day training interventions (courses) was identical regardless of the locality. |
| **Feedback**  **(**Feedback given to learners) | What? The content of the presentation / practice scenarios / feedback ... |  |
|  | How? The form of the presentation / practice scenarios / feedback ... (e.g., sequencing, scaffolds etc.) | No feedback on scores or answers was given to participants or trainers either before or after training. *pg 1535* |
|  | Medium? The medium used (on paper, lecture, group discussion, simulation etc.) |  |
| **Assessment**  **(**Assessing the learners) | What? The content of the presentation / practice scenarios / feedback ... | Questions related to the incidence, risk factors, emergency management and drug treatment of the following obstetric emergencies: basic life support, advanced life support, hypertensive disorders of pregnancy, shoulder dystocia, breech, twins, cord prolapse, postpartum haemorrhage and electronic fetal monitoring. *pg 1535* |
|  | How? The form of the presentation / practice scenarios / feedback ... (e.g., sequencing, scaffolds etc.) | Pretraining assessment in the form of a MCQ was undertaken 1–3 weeks prior to the training intervention. Post-training assessment was undertaken 1–3 weeks after the training intervention using the same bank of MCQ questions but in a different order. The variations in time intervals for the assessments were due to pre-existing factors, including other local courses, school holidays and availability of the simulation centre. All participants were given 45 minutes to complete the 185 questions, negatively marked, true/false/don’t know MCQs under exam conditions. The MCQ was produced by an expert panel of midwives and obstetricians and had been adapted from a validated 240 question MCQ bank used during the pilot phase of the study. Following the pilot, questions that were poorly discriminating were removed. *pg 1535* |
|  | Medium? The medium used (on paper, lecture, group discussion, simulation etc.) |  |
|  | | |
| **Paper # 19 Siassakos** | | |
| **Title** | | Content analysis of team communication in an obstetric emergency scenario |
| **Authors** | | D. SIASSAKOS, T. DRAYCOTT, I. MONTAGUE& M. HARRIS |
| **Journal & Publishing information** | | Journal of Obstetrics and Gynaecology, August 2009; 29(6): 499–503 |
| **Abstract** | | We studied a random sample of four teams of doctors and midwives who participated in a videotaped simulated obstetric emergency, postpartum haemorrhage, before and after ‘on-site’ clinical training. We aimed to assess the validity of mixed techniques for the analysis of team communication and whether clinical and non-clinical team training improve communication. Two of the four teams received extra training in specific teamwork behaviours (TW+), the other half received only clinical training (TW-). We used content analysis to identify changes in communication patterns. There was a reduction in the total number of communications after training. Teams that received additional teamwork training used more directed commands after training. When commands were directed to specific individuals, tasks were more likely to be acknowledged and performed. We conclude that ‘on-site’ clinical drills can improve team communication in simulated emergencies, and additional teamwork training might improve this further, but it has to be refined and made obstetric specific first.  **Keywords:** Drills, simulation, task coordination, teamwork, training |
| **Presentation**  **(**Information presented to learners) | What? The content of the presentation / practice scenarios / feedback ... | All teams received clinical training but only two out of four teams received additional aviation-based crew resource management (CRM) team-work training: roles and responsibilities, clear directed communication, situational awareness. *pg 500* |
|  | How? The form of the presentation / practice scenarios / feedback ... (e.g., sequencing, scaffolds etc.) |  |
|  | Medium? The medium used (on paper, lecture, group discussion, simulation etc.) |  |
| **Practice**  **(**Practice provided to learners) | What? The content of the presentation / practice scenarios / feedback ... | Two of the four teams received extra training in specific teamwork behaviours (TW+), the other half received only clinical training (TW-). Audio-video recordings of eight postpartum haemorrhage simulations were collected in 2004 for the initial phase of a study of the evaluation of training modalities for the management of obstetric emergencies (the ‘SaFE’ study). *pg 500* |
|  | How? The form of the presentation / practice scenarios / feedback ... (e.g., sequencing, scaffolds etc.) | The participants were a stratified random sample of four groups (one senior, one junior doctor and two senior, two junior midwives per group). Each group took part in a simulation of a major primary postpartum haemorrhage before and after their training courses for the management of obstetric emergencies. The eight video-recordings were reviewed and transcribed to computer text files. The simulations comprised 58 min of audio-video recordings with an average duration of 7 min. *pg 500* |
|  | Medium? The medium used (on paper, lecture, group discussion, simulation etc.) | The simulations were conducted in the delivery room of the labour ward, with a healthcare assistant as patient-actress. Clinical information was provided, when necessary, on an automated computer slideshow. Additional information could be requested via an intercom. Midwives were given a patient history in the form of a shift handover. One midwife was allocated as the primary carer and was given a further briefing in the delivery room. The team members entered the room when requested.  Three small unobtrusive cameras, connected to a digital- recorder in a nearby room, captured time-stamped audio- video data. *pg 500* |
| **Feedback**  **(**Feedback given to learners) | What? The content of the presentation / practice scenarios / feedback ... |  |
|  | How? The form of the presentation / practice scenarios / feedback ... (e.g., sequencing, scaffolds etc.) |  |
|  | Medium? The medium used (on paper, lecture, group discussion, simulation etc.) |  |
| **Assessment**  **(**Assessing the learners) | What? The content of the presentation / practice scenarios / feedback ... |  |
|  | How? The form of the presentation / practice scenarios / feedback ... (e.g., sequencing, scaffolds etc.) |  |
|  | Medium? The medium used (on paper, lecture, group discussion, simulation etc.) |  |
|  | | |
| **Paper # 20 Straub** | | |
| **Title** | | Targeted obstetric haemorrhage programme improves incoming resident confidence and knowledge. |
| **Authors** | | [Straub HL](http://www.ncbi.nlm.nih.gov/pubmed/?term=Straub%20HL%5BAuthor%5D&cauthor=true&cauthor_uid=24219716)^1^, [Morgan G](http://www.ncbi.nlm.nih.gov/pubmed/?term=Morgan%20G%5BAuthor%5D&cauthor=true&cauthor_uid=24219716), [Ochoa P](http://www.ncbi.nlm.nih.gov/pubmed/?term=Ochoa%20P%5BAuthor%5D&cauthor=true&cauthor_uid=24219716), [Grable I](http://www.ncbi.nlm.nih.gov/pubmed/?term=Grable%20I%5BAuthor%5D&cauthor=true&cauthor_uid=24219716), [Wang E](http://www.ncbi.nlm.nih.gov/pubmed/?term=Wang%20E%5BAuthor%5D&cauthor=true&cauthor_uid=24219716), [Kharasch M](http://www.ncbi.nlm.nih.gov/pubmed/?term=Kharasch%20M%5BAuthor%5D&cauthor=true&cauthor_uid=24219716), [Plunkett BA](http://www.ncbi.nlm.nih.gov/pubmed/?term=Plunkett%20BA%5BAuthor%5D&cauthor=true&cauthor_uid=24219716). |
| **Journal & Publishing information** | | [J Obstet Gynaecol.](http://www.ncbi.nlm.nih.gov/pubmed/24219716) 2013 Nov;33(8):798-801. doi: 10.3109/01443615.2013.816668 |
| **Abstract** | | Postpartum haemorrhage is an infrequent but potentially life-threatening obstetrical emergency amenable to simulation. An educational programme consisting of a lecture and high-fidelity simulation exercise was given to incoming obstetrics and gynaecology (OB) and family medicine (FM) residents. Residents reported pre- and post-intervention confidence scores on a 1–5 Likert scale and a subset completed a postpartum haemorrhage knowledge assessment. Residents reported significant improvements in confidence in parameters involved in diagnosis and management of postpartum haemorrhage. The postpartum haemorrhage test mean scores significantly increased (57.4 + 9.6% vs 77.1 + 7.9%, *p* < 0.01) and were significantly correlated to confidence scores (Spearman’s coefficient of 0.651, *p* < 0.001). In conclusion, an education programme that incorporates high-fidelity simulation of postpartum haemorrhage improves the confidence and knowledge of incoming residents and appears to be an effective educational approach. **Keywords**: Obstetric haemorrhage, resident orientation, simulation education |
| **Presentation**  **(**Information presented to learners) | What? The content of the presentation / practice scenarios / feedback ... | The postpartum haemorrhage education portion involved a 1.5 h lecture directed at the diagnosis, management and treatment of obstetric haemorrhage. A standardised lecture developed by the Illinois Department of Public Health as part of its mandated obstetric haemorrhage education initiative was utilised (Obstetric Haemorrhage Education Project Workgroup 2008). Recognition of obstetric haemorrhage and appropriate evaluation and treatment of common aetiologies (i.e. uterine atony) were emphasised. In addition, a review of our institutional protocols in response to haemorrhage was included. *pg 799* |
|  | How? The form of the presentation / practice scenarios / feedback ... (e.g., sequencing, scaffolds etc.) | The orientation day consisted of a series of lectures by the OB faculty, followed by clinical simulations. *pg 799* |
|  | Medium? The medium used (on paper, lecture, group discussion, simulation etc.) |  |
| **Practice**  **(**Practice provided to learners) | What? The content of the presentation / practice scenarios / feedback ... | After the lecture, the residents participated in a blood estimation lab, in which they observed a series of stations with sponges, laparotomy pads and other common delivery containers filled with varying amounts of simulated blood. Residents were asked to estimate the amount of blood contained at each station before the results were revealed. In addition, we selected a simulation scenario that has been described in the literature (Maslovitz et al. 2007; Deering et al. 2009) and recommended by national organisations (Joint Commission on Accreditation of Healthcare Organizations 2004; RCOG 2004). *pg 800* |
|  | How? The form of the presentation / practice scenarios / feedback ... (e.g., sequencing, scaffolds etc.) | Finally, the residents participated in a 10–15 min high-fidelity clinical simulation using the Noelle Simulator (Gaumard Scientific, Miami FL), where they were asked to attend a vaginal delivery with a subsequent postpartum haemorrhage.  Our postpartum haemorrhage lecture and knowledge assessment were both a component of state-mandated training. We utilised a high-fidelity simulation experience following a didactic lecture to allow the opportunity to first acquire and then apply new knowledge to attain confidence. *pg 799* |
|  | Medium? The medium used (on paper, lecture, group discussion, simulation etc.) | The simulator was programmed to have changes in vital signs dependent on the actions of the resident. An actress outside the room played the voice of the patient. A nurse was available in the room to help with laboratory assessment and medication administration; periodically, the nurse would check the patient and reveal more simulation blood loss on the perineal padding. *pg 799* |
| **Feedback**  **(**Feedback given to learners) | What? The content of the presentation / practice scenarios / feedback ... |  |
|  | How? The form of the presentation / practice scenarios / feedback ... (e.g., sequencing, scaffolds etc.) | An OB faculty member was in the room to provide immediate feedback and help if the resident was unable to proceed independently. After the simulation, a short debriefing session was held. *pg 799* |
|  | Medium? The medium used (on paper, lecture, group discussion, simulation etc.) |  |
| **Assessment**  **(**Assessing the learners) | What? The content of the presentation / practice scenarios / feedback ... | The resident was expected to recognise the haemorrhage, call for assistance, activate the institutional haemorrhage protocol and initiate treatment. A background survey was given to the residents to assess the extent of their experience and training in uncomplicated vaginal deliveries and obstetric emergencies, prior to their participation in the orientation programme.  The residents were asked to complete an anonymous survey immediately before and after the programme, rating their level of confidence in their ability to successfully perform tasks related to a postpartum haemorrhage. As part of the 2011 simulation, residents were asked to complete a 25-question state-mandated exam on diagnosing and managing postpartum haemorrhage (Obstetric Haemorrhage Education Project Workgroup 2008). Residents participating in the 2011 simulation also filled out an evaluation of the simulation day using the standardised form from the NorthShore Center for Simulation Technology and Academic Research on a 1–5 Likert Scare, where 1 = needs improvement and 5 = outstanding. *pg 799* |
|  | How? The form of the presentation / practice scenarios / feedback ... (e.g., sequencing, scaffolds etc.) | This survey asked residents to rate their level of confidence on a 1–5 Likert scale, where 1 was defined as ‘Not confident’ and 5 as ‘Very confident’, in their ability to successfully perform delineated tasks. The haemorrhage exam was given before and after the educational programme. *pg 799* |
|  | Medium? The medium used (on paper, lecture, group discussion, simulation etc.) |  |
|  | | |
| **Paper # 21 Vadnais** | | |
| **Title** | | [Assessment of long-term knowledge retention following single-day simulation training for uncommon but critical obstetrical events.](http://www.ncbi.nlm.nih.gov/pubmed/22191668) |
| **Authors** | | Vadnais MA, Dodge LE, Awtrey CS, Ricciotti HA, Golen TH, Hacker MR. |
| **Journal & Publishing information** | | J Matern Fetal Neonatal Med. 2012 Sep;25(9):1640-5. doi: 10.3109/14767058.2011.648971. Epub 2012 Apr 25. |
| **Abstract** | | **Objective—**The objectives were to determine (i) whether simulation training results in short- term and long-term improvement in the management of uncommon but critical obstetrical events and (ii) to determine whether there was additional benefit from annual exposure to the workshop.  **Methods—**Physicians completed a pretest to measure knowledge and confidence in the management of eclampsia, shoulder dystocia, postpartum hemorrhage and vacuum-assisted vaginal delivery. They then attended a simulation workshop and immediately completed a posttest. Residents completed the same posttests 4 and 12 months later, and attending physicians completed the posttest at 12 months. Physicians participated in the same simulation workshop 1 year later and then completed a final posttest. Scores were compared using paired t-tests.  **Results—**Physicians demonstrated improved knowledge and comfort immediately after simulation. Residents maintained this improvement at 1 year. Attending physicians remained more comfortable managing these scenarios up to 1 year later; however, knowledge retention diminished with time. Repeating the simulation after 1 year brought additional improvement to physicians.  **Conclusion—**Simulation training can result in short-term and contribute to long-term improvement in objective measures of knowledge and comfort level in managing uncommon but critical obstetrical events. Repeat exposure to simulation training after 1 year can yield additional benefits.  **Keywords**: Long-term follow up; medical education; obstetric emergencies; simulation |
| **Presentation**  **(**Information presented to learners) | What? The content of the presentation / practice scenarios / feedback ... | Resident and attending physicians completed a simulation workshop on the management of eclampsia, shoulder dystocia, postpartum hemorrhage and Kiwi® vacuum-assisted vaginal delivery. *pg 2* Following completion of the pretest, participants attended a 1-hour didactic session that devoted approximately 15 minutes to each clinical scenario and served as an introduction to the simulation topics. *pg 3* |
|  | How? The form of the presentation / practice scenarios / feedback ... (e.g., sequencing, scaffolds etc.) |  |
|  | Medium? The medium used (on paper, lecture, group discussion, simulation etc.) |  |
| **Practice**  **(**Practice provided to learners) | What? The content of the presentation / practice scenarios / feedback ... |  |
|  | How? The form of the presentation / practice scenarios / feedback ... (e.g., sequencing, scaffolds etc.) | This (the didactic session) was followed by a simulation training with one station for each of the four clinical scenarios. The simulation was designed so that all four tasks could be completed within 60–90 minutes. Each station was co-led by an attending physician with expertise in the area and a chief resident. Nurses, midlevel providers and scrub techs participated as trainers playing the roles they would play in live situations. *pg 3* |
|  | Medium? The medium used (on paper, lecture, group discussion, simulation etc.) | The postpartum hemorrhage station utilized a uterine model that was not commercially available and was built for the purpose of this workshop. It consisted of a golf club cover as the uterus and a rubber catheter as the uterine vessels. Station leaders described a clinica scenario of postpartum hemorrhage secondary to atony. Participants communicated steps for medical management of atony, including the use of uterotonics (names, doses, maximum doses and contraindications) and steps for fluid resuscitation and transfusion. Participants then proceeded with laparotomy and surgical management of atony. Station leaders supervised as participants ligated the uterine vessels; placed B lynch sutures and box stitches; and utilized Bakri balloon catheters. *pg 3/4* |
| **Feedback**  **(**Feedback given to learners) | What? The content of the presentation / practice scenarios / feedback ... |  |
|  | How? The form of the presentation / practice scenarios / feedback ... (e.g., sequencing, scaffolds etc.) |  |
|  | Medium? The medium used (on paper, lecture, group discussion, simulation etc.) |  |
| **Assessment**  **(**Assessing the learners) | What? The content of the presentation / practice scenarios / feedback ... | The extent of learning that resulted from the workshop was assessed with a pretest and posttest that included 35 multiple-choice questions designed to evaluate knowledge. All but two of the 35 multiple-choice questions were based on the relevant American Congress of Obstetricians and Gynecologists practice bulletins. The remaining two questions were based on other well-accepted practice guidelines. *pg 2/3* |
|  | How? The form of the presentation / practice scenarios / feedback ... (e.g., sequencing, scaffolds etc.) | A 10-point Likert scale was used to elicit subjective self-reported comfort level in managing each of the clinical scenarios, with 1 being not at all comfortable and 10 being extremely comfortable. The pretest included one Likert scale question for each of the four clinical scenarios. The posttest was identical to the pretest with the exception of one additional question for each clinical scenario that asked whether learners felt more, equally or less able to manage each scenario after the simulation compared with before. The pretest was administered immediately before the start of the simulation workshop. They then attended a simulation workshop and immediately completed a posttest. Immediately following the simulation, participants completed the posttest. To examine their experience as co-leaders, chief residents also completed a survey with seven Likert scale questions and one open-ended question. Resident physicians completed additional posttests at 4 and 12 months, and attending physicians completed a posttest at 12 months. After completing the posttest at 12 months, both resident and attending physicians completed the single-day simulation workshop for the second time. Immediately following this repeat simulation workshop, they completed the final posttest. *pg 3* |
|  | Medium? The medium used (on paper, lecture, group discussion, simulation etc.) |  |

1. **Articles' Tables PPH Simulation & ID Subset22to28**

| **Article # 22 Kato** | | |
| --- | --- | --- |
| **Title** | | Simulation training program for midwives to manage postpartum hemorrhage: A randomized controlled trial |
| **Authors** | | Chiho Kato, Yaeko Kataoka |
| **Journal & Publishing information** | | Nurse Education Today 51 (2017) 88–95 |
| **Abstract** | | **Purpose:** To explore the effectiveness of a simulation training program for midwives in performance and knowledge for the management of postpartum hemorrhage (PPH).  **Methods:** The study design was a randomized controlled trial. Midwives working at one obstetrics ward in an urban area were randomly assigned to simulation training program or no training. This “simulation program” included pre study e-learning and simulation. Inclusion criteria were midwives who: 1) had two or three years of clinical experience, 2) worked in an obstetrics ward, and 3) had experience with birth assistance. There was one exclusion criterion namely prior experience of simulation training for PPH. Change in performance was evaluated using a PPH scenario performance test at one month after the simulation training. Change in knowledge was evaluated by a 25-item multiple-choice questionnaire completed shortly before the training and one month after the training. The ethical review committee of St Luke's International University granted approval (No. 14- 096).  **Results:** Eighty-one midwives were randomly assigned to either the intervention group (n = 40) or the control group (n = 41). Performance in the simulation training group was significantly better in comparison to the no training group; mean performance score was 23.85(SD 2.71) in the training group versus 18.00(SD 3.01) in the no training group (MD 5.85 95% CI 4.85–7.12, t = 9.17, p b 0.001). Knowledge was significantly increased in the simulation training group; amount of knowledge score was 3.65(SD 3.40) in the training group versus −0.02(SD 3.02) in the no training group (MD 3.67 95% CI 2.25–5.10, t = 5.14, p b 0.001).  **Conclusion:** Both performance and knowledge about the management of PPH were significantly improved after simulation training. However, assessments of long-term effects on performance, and knowledge and the clinical outcomes in managing of obstetric complications are necessary to adequately evaluate the effectiveness of simulation training. |
| **Presentation**  **(**Information presented to learners) | What? The content of the presentation / practice scenarios / feedback ... | Midwives allocated to the intervention group took the simulation training program. This program included a pre study e-learning for PPH conducted the day before the simulation training. The pre study e-learning program was developed by an obstetrician and four mid- wives based on Japanese national guidelines (Japan Society of Obstetric and Gynecology, 2010), and consisted of four modules: 1) physiology of PPH, 2) assessment of hemorrhagic shock, 3) managing PPH (hemostatic approach, injection, blood transfusion), and 4) role play video on managing PPH (Table 1). *Pg89* |
|  | How? The form of the presentation / practice scenarios / feedback ... (e.g., sequencing, scaffolds etc.) | This program included a pre study e-learning for PPH conducted the day before the simulation training. Each module was approximately 15 min. …In addition, partici- pants could log out of each module and resume at any time. *Pg89* |
|  | Medium? The medium used (on paper, lecture, group discussion, simulation etc.) | This program included a pre study e-learning for PPH conducted the day before the simulation training. *Pg89* |
| **Practice**  **(**Practice provided to learners) | What? The content of the presentation / practice scenarios / feedback ... | We used scenarios for managing PPH that consisted of three sections: 1) first response to PPH/exploring the cause of the bleeding, 2) treatment of hemorrhagic shock, and 3) blood transfusion and disseminated intravascular coagulation (DIC). *Pg89* |
|  | How? The form of the presentation / practice scenarios / feedback ... (e.g., sequencing, scaffolds etc.) | The scenarios started with about a 10-minute briefing (introduc- tion on simulation training), where the clinical situation is detailed using a Power Point presentation. After the introduction, participants started the 5-minute initial simulation training. … *Pg89*  The simulation training program was implemented using low-fidelity mannequins with patient-actors. Low fidelity mannequins were selected in the absence of convincing data that high fidelity mannequins provide superior outcomes. After training, they underwent a debriefing session for 15 minThen, participants performed a second simulation training and debriefing for approximately 5 min. A single session lasted 40–50 min, and the overall simulation training- program was about 3 h, including practical skills training (Table 1). *Pg89* |
|  | Medium? The medium used (on paper, lecture, group discussion, simulation etc.) | The simulation training program was implemented using low-fidelity mannequins with patient-actors. Low fidelity mannequins were selected in the absence of convincing data that high fidelity mannequins provide superior outcomes. After training, they underwent a debriefing session for 15 min. *Pg90* |
| **Feedback**  **(**Feedback given to learners) | What? The content of the presentation / practice scenarios / feedback ... | After training, they underwent a debriefing session for 15 min (feedback about their medical technical skills and teamwork were provided by a training facilitator). *Pg89* |
|  | How? The form of the presentation / practice scenarios / feedback ... (e.g., sequencing, scaffolds etc.) |  |
|  | Medium? The medium used (on paper, lecture, group discussion, simulation etc.) |  |
| **Assessment**  **(**Assessing the learners) | What? The content of the presentation / practice scenarios / feedback ... | Furthermore, we checked access to the time the person logged on and how long they were logged in as documented on the manager page. An identification of completion was published when they finished e-learning that all participants brought to the simulation training. *Pg89*  The primary outcome was a change in the performance score one month after the simulation training program. The secondary outcome was the amount of change in knowledge as measured by a multiple-choice questionnaire (MCQ). *Pg90*  …Moreover, the intervention and control groups were asked to fill out a self-assessment questionnaire if they had encountered PPH (hemorrhaging of 1000 mL or more) in their clinical setting during the month after taking the simulation training program. The questions were 1) Were you able to make an appropriate assessment? 2) Were you able to appropriately respond? and 3) Do you think teamwork was achieved? There were 4 possible responses, from “I definitely think so” to “I definitely don't think so” (Table 4). |
|  | How? The form of the presentation / practice scenarios / feedback ... (e.g., sequencing, scaffolds etc.) | The overall performance assessment took approximately 15 min. *Pg90*  The same trained assessor (with clinical experience of N 3 years) who was blinded to group allocation conducted all the performance assessments. Performance in a PPH scenario was measured by a checklist of key actions for PPH management based on national guidelines. *Pg90*  The secondary outcome was the amount of change in knowledge as measured by a multiple-choice questionnaire (MCQ). We conducted a pilot test for the pre study e-learning on 48 midwives who worked on obstetrical units in an urban area of Japan and had a range of clinical ex- perience from 1 to over 10 years. *Pg90*  …Moreover, the intervention and control groups were asked to fill out a self-assessment questionnaire if they had encountered PPH (hemorrhaging of 1000 mL or more) in their clinical setting during the month after taking the simulation training program. The questions were 1) Were you able to make an appropriate assessment? 2) Were you able to appropriately respond? and 3) Do you think teamwork was achieved? There were 4 possible responses, from “I definitely think so” to “I definitely don't think so” (Table 4). |
|  | Medium? The medium used (on paper, lecture, group discussion, simulation etc.) | Performance in a PPH scenario was measured by a checklist of key actions for PPH management based on national guidelines. The performance checklist consisted of 17 items in five domains: 1) first response to PPH, 2) exploring the cause of bleeding, 3) treatment of hemorrhagic shock, 4) blood transfu- sion and DIC, and 5) communication. The checklist was graded with a 0, 1, or 2, where a score of 2 indicated appropriate care. The total performance score ranged from 0 to 34, with a higher score representing more appropriate care. The performance checklist had high inter-rater reliability (Interclass Correlation Coefficient: ICC 0.954) (Kato et al., 2015a, 2015b). *Pg90*  …. Changes in the knowledge score pre- test and post-test were analyzed by using a paired t-test. The knowledge score significantly increased after the program (t = 10.27, p b 0.001) (Kato et al., 2015a, 2015b). *Pg90*  After the pilot test, the knowledge test was reworked into 25 items. The 25-item knowledge test consisted of 2 domains, basic knowledge and management of PPH. MCQ was graded as 0 (false) or 1 (true). The total knowledge score ranged from 0 to 25, with a higher score representing a greater amount of knowledge. The MCQ as revised was conducted prior to the pre study e-learning, one day before and then one month after the simulation training. We excluded the pilot group from the main study. |
|  | | |
| **Article # 23 DeMelo** | | |
| **Title** | | **The use of instructional design guidelines to increase effectiveness of postpartum hemorrhage simulation training** |
| **Authors** | | Brena C.P. de Melo, Ana R. Falbo, Arno M.M. Muijtjens, Cees P.M. van der Vleuten, Jeroen J.G. van Merriënboer |
| **Journal & Publishing information** | | *Int J Gynecol Obstet* 2017; 1–7 |
| **Abstract** | | **Objective:** To compare learning outcomes of postpartum hemorrhage simula on training based on either instructional design guidelines or best practice.  **Methods:** A pretest–post-test non-equivalent groups study was conducted among obstetrics and gynecology residents in Recife, Brazil, from June 8 to August 30, 2013. The instructional design group included 13 teams, whereas the best practice group included seven teams. A standardized task checklist was used for scenario analysis and the propor on of correctly executed tasks compared (post- test minus pretest).  **Results:** The instructional design group scored higher than the best prac ce group for total number of tasks completed (median di erence 0.46 vs 0.17; *P<*0.001; effect size [*r*]=0.72). Similar results were observed for communication (median difference 0.56 vs 0.22; *P=*0.004; *r=*0.58), laboratory evaluation (median difference 0.83 vs 0.00; *P<*0.001; *r=*0.76), and mechanical management (median difference 0.25 vs –0.15; *P=*0.048; *r=*0.39). Speed of learning was also increased. The median differences were 0.20 for the instructional design group compared with 0.05 for the best practice group at 60 seconds (*P=*0.015; *r=*0.49), and 0.49 versus 0.26 (*P=*0.001; *r=*0.65) at 360 seconds.  **Conclusion:** The use of simulation training for postpartum hemorrhage that was based on instructional design guidelines yielded better learning outcomes than did training based on best practice. |
| **Presentation**  **(**Information presented to learners) | What? The content of the presentation / practice scenarios / feedback ... | The aim of the present study was to test the hypothesis that a PPH simulation training program based on instructional design guidelines would lead to better learning outcomes than the use of a simulation training program based on best practice. *Pg2*  To clearly establish the learning objec ves of the present study, a PPH guideline was created through discussion with a focus group (formed of invited obstetricians and anesthesiologists who were supervisors of residents) and was based on the most up- to- date clin- ical evidence. Table 1 provides examples of the expected tasks by subscale. *Pg2* **Subscale** Communication **Expected task** The participant introduced himself or herself to the patient, explained the actions to the patient, and asked the patient about comorbidities, medications, and allergies. **Subscale** Teamwork **Expected task** Team- support behavior and adequate sharing of information  **Subscale** Vital signs **Expected task** Checked heart rate, blood pressure, and rechecked vital signs **Subscale** Venous access  **Expected task** Large caliber intravenous line, order blood samples **Subscale** Laboratory evaluation **Expected task** Complete blood count; renal and liver func on tests; blood type and cross- match **Subscale** Drug management **Expected task** Loading and maintenance doses of oxytocin **Subscale** Mechanical management  **Expected task** Massaged uterus, emptied bladder, and checked for lacerations  **Subscale** Surgical management  **Expected task** Suture or placental ssue removal if the scenario required action |
|  | How? The form of the presentation / practice scenarios / feedback ... (e.g., sequencing, scaffolds etc.) | Figure 1 outlines the two training formats and the clinical case scenarios. The instructional design group format comprised eight steps, including three training scenarios with increasing levels of complexity. An obstetrician was present in the delivery room to provide “over the shoulder” just-in-time guidance and corrective feedback to participants, whenever necessary. The best practice group received the PPH guideline before undergoing a three-step training format, one of which was a training scenario. This format replicated the best PPH simula on training11 and was creating using articles identied in the literature. Seven experts (five from an educational background and two who had healthcare training) used a rating scale based on Merrill’s First Principles of Instruction8 to analyze the articles from an instructional design perspective. *Pg2* |
|  | Medium? The medium used (on paper, lecture, group discussion, simulation etc.) | At each training session, participants were welcomed, introduced to the training equipment, and asked to suspend their disbelief (i.e. act as though the simulation was a real-life clinical case) and not to share any informa on about the program until the end of all training sessions. *Pg3* |
| **Practice**  **(**Practice provided to learners) | What? The content of the presentation / practice scenarios / feedback ... | All scenarios were scripted as PPH after vaginal delivery and included a standardized patient, a standardized nurse, and a part-task pelvis simulator (EVA Simulador Pós- Parto; ProDelphus, Olinda, Brazil). *Pg2* |
|  | How? The form of the presentation / practice scenarios / feedback ... (e.g., sequencing, scaffolds etc.) | All scenarios were scripted as PPH after vaginal delivery and included a standardized patient, a standardized nurse, and a part-task pelvis simulator (EVA Simulador Pós- Parto; ProDelphus, Olinda, Brazil). Consequently, the nature of poten ally expected tasks comprised communication, teamwork, and clinical management. *Pg2*  *See Figure 1 on page3* |
|  | Medium? The medium used (on paper, lecture, group discussion, simulation etc.) | The low-cost part-task simulator allowed varia on in the site (vaginal laceration, uterus, or both) and intensity of the bleed, and was managed by the standardized patient. The standardized nurses provided the par cipants with clinical data (vital signs, results of laboratory evaluation, and uterine responses). Both the standardized patients and nurses (who were all volunteer healthcare personnel) received the script for each scenario in advance of training to allow for rehearsal. Par cipants were then asked to form the teams outlined above and provided with the pretest scenario. All scenarios were either run for a maximum of 900 seconds or else interrupted at the point that the team diagnosed and corrected the main cause of the bleeding. *Pg3* |
| **Feedback**  **(**Feedback given to learners) | What? The content of the presentation / practice scenarios / feedback ... | All scenarios were video recorded for either debriefing purposes (the training scenarios) or to enable future analysis for scoring and comparisons (the pretest and post-test scenarios). |
|  | How? The form of the presentation / practice scenarios / feedback ... (e.g., sequencing, scaffolds etc.) | An obstetrician was present in the delivery room to provide “over the shoulder” just- in- me guidance and correc ve feedback to par- cipants, whenever necessary. *Pg2*  Two obstetricians were present at each session: BdM attended all the training sessions and 10 additional obstetricians alternated as the second content expert. Each pair of obstetricians was responsible for rating the pretest and post-test scenarios video recordings for each of the teams that they supervised. *Pg3* |
|  | Medium? The medium used (on paper, lecture, group discussion, simulation etc.) | The experts received the checklists of expected tasks at the beginning of each scenario, whereas the participants received them immediately after attending the first and third training scenario for debriefing purposes. A standardized checklist of expected tasks (derived from the PPH guideline outlined previously) for each scripted scenario was used in the analysis. A task was considered to be executed if it had been performed by at least one participant (e.g. ordering laboratory evalua on) or, depending on the nature of the task, by all members of the team (i.e. teamwork tasks). Consequently, the unit of analysis was the team rather than individual par cipants. |
| **Assessment**  **(**Assessing the learners) | What? The content of the presentation / practice scenarios / feedback ... | All scenarios were video recorded for either debriefing purposes (the training scenarios) or to enable future analysis for scoring and comparisons (the pretest and post-test scenarios). |
|  | How? The form of the presentation / practice scenarios / feedback ... (e.g., sequencing, scaffolds etc.) | The pretest and post-test scenarios were the same for both groups. They were offered immediately before and after the training scenarios, and presented an intermediate level of complexity. |
|  | Medium? The medium used (on paper, lecture, group discussion, simulation etc.) | All scenarios were video recorded for either debrie ng purposes (the training scenarios) or to enable future analysis for scoring and comparisons (the pretest and post- test scenarios). |
|  | | |
| **Article # 24 Egenberg** | | |
| **Title** | | Changes in self-efficacy, collective efficacy, and patient outcome following interprofessional simulation training on postpartum haemorrhage |
| **Authors** | | Signe Egenberg, Pål Øian, Torbjørn Moe Eggebø, Mirjana Grujic Arsenovic, Lars Edvin Bru |
| **Journal & Publishing information** | | Journal of Clinical Nursing Accepted Date : 12-Nov-2016 |
| **Abstract** | | **Aims and objectives.** To examine whether interprofessional simulation training on management of postpartum haemorrhage enhances self-efficacy and collective efficacy and reduces the blood transfusion rate after birth.  **Background.** Postpartum haemorrhage is a leading cause of maternal morbidity and mortality worldwide, although it is preventable in most cases. Interprofessional simulation training might help improve the competence of health professionals dealing with postpartum haemorrhage, and more information is needed to determine its potential.  **Design.** Multimethod, quasi-experimental, pre-post intervention design.  **Methods.** Interprofessional simulation training on postpartum haemorrhage was implemented for midwives, obstetricians, and auxiliary nurses in a university hospital. Training included realistic scenarios and debriefing, and a measurement scale for perceived postpartum haemorrhage-specific self-efficacy and collective efficacy was developed and implemented. Red blood cell transfusion was used as the dependent variable for improved patient outcome pre-post intervention.  **Results.** Self-efficacy and collective efficacy levels were significantly increased after training. The overall red blood cell transfusion rate did not change, but there was a significant reduction in the use of ≥5 units of blood products related to severe bleeding after birth.  **Conclusion.** The study contributes to new knowledge on how simulation training through mastery and vicarious experiences, verbal persuasion and psychophysiological state might enhance postpartum haemorrhage-specific self-efficacy and collective efficacy levels and thereby predict team performance. The significant reduction in severe postpartum haemorrhage after training, indicated by reduction in ≥5 units of blood transfusions, corresponds well with the improvement in collective efficacy, and might reflect the emphasis on collective efforts to counteract severe cases of postpartum haemorrhage. |
| **Presentation**  **(**Information presented to learners) | What? The content of the presentation / practice scenarios / feedback ... | According to the PPH protocol, the first steps to be made, were; call for help, give additional oxytocin, secure IV access, empty urine bladder, give misoprostol and perform bimanual uterine compression. In case of PPH and retained placenta, manual removal was to be done in the operating theatre. |
|  | How? The form of the presentation / practice scenarios / feedback ... (e.g., sequencing, scaffolds etc.) |  |
|  | Medium? The medium used (on paper, lecture, group discussion, simulation etc.) |  |
| **Practice**  **(**Practice provided to learners) | What? The content of the presentation / practice scenarios / feedback ... | During an eight-hour training day, 2 of the 5 scenarios focused on PPH, in accordance with the PPH protocol for the hospital and based on national guidelines.  There were four learning goals for the team in the PPH scenario: (1) treat PPH according to guidelines; (2) develop communication skills (e.g. closed loop-communication); (3) perform uterine massage; and (4) exercise clear leadership. In the actual PPH scenario, the bleeding was caused by uterine atony, and the facilitator provided information on request that could not be obtained by examination of MamaNatalie®, like blood pressure, pulse, and whether the skin was cold and clammy. Additionally, the team had to treat the operator as an anxious mother who required attention, using non-technical skills like communication, leadership, and teamwork. The scenario ended when the team controlled the bleeding and stabilised the mother or decided to transfer her to the operating theatre. |
|  | How? The form of the presentation / practice scenarios / feedback ... (e.g., sequencing, scaffolds etc.) | A group of 8 fellow midwives/doctors prepared themselves to act as facilitators and operators during a 2-day facilitation course. They created realistic scenarios on obstetric emergencies like neonatal asphyxia, shoulder dystocia, and PPH.  Before the scenario, the facilitator briefly described the frames of the simulation training and presented the local PPH guidelines, the basic features of MamaNatalie®, learning goals for technical and non-technical skills, and the importance of a safe environment for the simulation. |
|  | Medium? The medium used (on paper, lecture, group discussion, simulation etc.) | The PPH scenario utilised the MamaNatalie® birthing simulator, which includes a baby, a uterus, a urine bladder, a placenta, and a blood tank containing up to 1500 ml of artificial blood (LGH 2015). The birthing simulator is strapped onto the operator, who acts as the labouring woman. The operator responds to the actions during the simulation, providing a high degree of realism. The PPH scenario requires technical skills like uterine massage and bimanual compression, and uterine contractility and blood loss are adjusted according to the team’s actions |
| **Feedback**  **(**Feedback given to learners) | What? The content of the presentation / practice scenarios / feedback ... | The facilitator debriefed the team with a “good judgement approach” that used her/his expert opinion while valuing the unique perspective of each participant (Rudolph et al. 2007). |
|  | How? The form of the presentation / practice scenarios / feedback ... (e.g., sequencing, scaffolds etc.) | During rigorous reflection, every trainee was challenged on his/her understanding of the PPH case and how it was managed, perception of individual and team achievements and preferable future actions. |
|  | Medium? The medium used (on paper, lecture, group discussion, simulation etc.) |  |
| **Assessment**  **(**Assessing the learners) | What? The content of the presentation / practice scenarios / feedback ... | A literature search performed in September 2013 revealed no published self-efficacy measurements related to PPH management. A measurement of PPH self-efficacy (PPHSE) was thus developed for this specific study.  The study population comprised all women who gave birth with gestational age ≥23 weeks at UNN Tromsø over four years: 2011 (n = 1419) and 2012 (n = 1284) births were the baseline (before implementation of training), and June 2013–May 2014 (n = 1382) and June 2014– May 2015 (n = 1361) births were used for patient outcome after training. |
|  | How? The form of the presentation / practice scenarios / feedback ... (e.g., sequencing, scaffolds etc.) | The study had a multimethod, quasi-experimental pre-post design that combined patient outcome with survey measures. A questionnaire was distributed to all trainees just before the initial training, three months later and three months after the second training, which was carried out a year after the initial training. Data on patient outcome were collected 24 months pre training (2011-2012) and 24 months post initial training (June 2013–May 2015). The self- report questionnaires and patient outcome added to the validity of the study by triangulating quantitative data from different sources. No other interventions to reduce transfusion rates were implemented during the study period.  SE and LEB developed items to assess PPHSE, discussed the items with an expert group of midwives and obstetricians, and revised the items according to their recommendations. A preliminary PPHSE-scale was pilot-tested among 59 midwives, 16 obstetricians and 4 auxiliary nurses employed at a different Norwegian university hospital, and minor adjustments were made. Questions on self-efficacy focused on individual perception of control. Items on PPH self-efficacy used an 8-point Likert scale that ranged from always (1 point) to never (8 points). The final PPHSE scale included 8 items (Appendix 1 shows item wording and Cronbach’s alphas for the self-report scales).  In health facilities, PPH is usually handled by a team of health workers. This study included two measurements related to the collective handling of PPH. The *Team Emergency Assessment Measure* (TEAM)-scale (Cooper & Cant 2014) is an established measure of team functioning in medical emergencies. The scale consists of eleven items with a 5-step scoring format that ranges from ‘Never/Hardly ever’ to ‘Always/Nearly always.’  There are indications that visual estimation of blood loss after birth is an unreliable measurement (Al-Kadri et al. 2014, Bose et al. 2006, Hancock et al. 2015), and number of units of blood products given to mothers after birth can indicate quantity of blood loss. We used the RBC transfusion rate as the dependent variable for mothers giving birth at UNN Tromsø 2 years before vs. after the first simulation training, distinguishing between the overall rate of RBC transfusion and severe PPH needing ≥5 units of RBCs, platelet concentrate, and/or FFP.  Data were also collected for maternal and labour characteristics for all mothers who received RBCs during their admission, to investigate whether changes in transfusion rates could be explained by these characteristics (e.g. parity, gestational age, unplanned or planned CS, placenta praevia/accreta/percreta, haemoglobin (Hb) level at discharge, and hysterectomy). Data were extracted from the hospital’s electronic birth registration system, the hospital’s electronic patient record system, and the registry of the Department of Laboratory Medicine. The birth cohort visitations did not change during the two periods. |
|  | Medium? The medium used (on paper, lecture, group discussion, simulation etc.) | Only people who participated in the training, were eligible to complete the surveys. The same questionnaire was used at all three time points. Of a total number of 104 employees, 82 participated in the initial training, and among the participants 88% (n = 72) answered the pre- test questionnaire. The post-test was distributed 3 months after the first training; of a total number of 82 employees who had participated, 62% of them responded (n = 51, of whom 8 had not answered the pre-test). The follow-up test was distributed 3 months after the second training; out of a total number of 106 employees, 85 participated in the second training and the response rate among them was 72% (n = 61, 20 of whom had not answered the pre- or post-tests). Among the participants, an average of 79% of the midwives answered the pre- test, post-test, and follow-up test, while 81% of the auxiliary nurses and 56% of the obstetricians did the same (Table 1, 2). |
|  | | |
| **Article # 25 Nathan** | | |
| **Title** | | Retention of skills 2 years after completion of a postpartum hemorrhage simulation training program in rural Rwanda |
| **Authors** | | Lisa M. Nathan, Desire Patauli, Damien Nsabimana, Peter S. Bernstein, Stephen Rulisa, Dena Goffman |
| **Journal & Publishing information** | | International Journal of Gynecology and Obstetrics xxx (2016) xxx–xxx http://dx.doi.org/10.1016/j.ijgo.2016.01.021 |
| **Abstract** | | **Objective:** To evaluate the long-term retention of skills gained by rural physicians who completed a postpartum hemorrhage simulation-training program. **Methods:** A quasi-experimental pre–post intervention study enrolled a convenience sample of generalist physicians in rural Rwanda. Participants underwent initial simulation training including pre- and post-training testing in February 2012. Simulation drills to assess skill retention were conducted in March 2014. Participants were scored based on their communication, evaluation, and management skills. Median scores and interquartile ranges were calculated and the Wilcoxon signed-rank sum test was used to compare the pre-training, post-training, and retention scores. Physician confidence was assessed using a survey.  **Results:** In total, 11 physicians were enrolled; eight were available for the 2-year skill-retention evaluation. Significant improvements were observed when comparing participants’ pre-training and post-training communication (P = 0.03), evaluation (P = 0.05), and management (P = 0.02) scores, and there were no changes between participants’ post-training and 2-year communication (PN0.99), evaluation (P=0.16), and manage- ment (P=0.46) scores. There were no differences in the self-reported confidence measures across the duration of the study.  **Conclusion:** Simulation training is an effective method for teaching postpartum hemorrhage- management skills to generalist physicians in rural areas and skills are retained for at least 2 years. Further studies could determine the optimal time intervals for refresher training. |
| **Presentation**  **(**Information presented to learners) | What? The content of the presentation / practice scenarios / feedback ... | In January 2012, two generalist physicians at a rural district hospital in Rwanda underwent a train-the-trainers program for postpartum hemorrhage management that incorporated simulation-based training. This program consisted of three components: an educational component, a simulation component, and leadership training. The educational component included didactics on normal labor, delivery, and obstetric hemorrhage, and a simulated postpartum hemorrhage scenario. Practice sessions of simulation scenarios were also conducted to allow participants to perfect their skills. The second component of the program involved teaching the partic- ipants how to set up and execute a postpartum hemorrhage-simulation drill using a basic birthing simulator, imitation blood, and actors; partic- ipants were taught how to make imitation blood from cornstarch and local fruit juice. Coaching was provided in writing a realistic postpartum hemorrhage scenario for an appropriate hospital setting and in recruiting local nurses to assist as actors/actresses. The final component of the train-the-trainers program was leadership training, using Jhpiego’s on- line ModCAL for Training Skills course [15], which is designed to improve the effectiveness of trainers. This course was completed in individual and group formats. |
|  | How? The form of the presentation / practice scenarios / feedback ... (e.g., sequencing, scaffolds etc.) |  |
|  | Medium? The medium used (on paper, lecture, group discussion, simulation etc.) |  |
| **Practice**  **(**Practice provided to learners) | What? The content of the presentation / practice scenarios / feedback ... | All participants completed an orientation course prior to simulation training; this in- cluded a vaginal delivery demonstration. This demonstration included an explanation of how to verbalize and perform tasks in order to receive credit during the simulation training. |
|  | How? The form of the presentation / practice scenarios / feedback ... (e.g., sequencing, scaffolds etc.) | The training program included individual, pre-training testing during a simulated postpartum hemorrhage drill, immediate individual performance feedback/debriefing, and a group lecture. During the study period at the end of each testing day, participants were permitted to complete an unrestricted volume of simulation practice using a manne- quin. Individual post-training testing, using the same simulated post- partum hemorrhage drill, was completed within 6–14 days to assess participants’ short-term retention of skills. |
|  | Medium? The medium used (on paper, lecture, group discussion, simulation etc.) | The postpartum hemorrhage simulation scenario included one actress portraying a “patient”, one actor/actress portraying a “nurse”, and one of the train-the-trainers participants who acted as a rater to assess each participant’s performance. The “patient” was fully clothed and was sat on the bed in a cross-legged position with a mannequin pelvis in front of her at her feet. Typical local fabric was placed at her waist and extended to cover the mannequin to give the appearance of being one "person". The “patient” controlled the release of blood from under the fabric, with the “nurse” present, caring for the “patient” during the scenario. |
| **Feedback**  **(**Feedback given to learners) | What? The content of the presentation / practice scenarios / feedback ... |  |
|  | How? The form of the presentation / practice scenarios / feedback ... (e.g., sequencing, scaffolds etc.) |  |
|  | Medium? The medium used (on paper, lecture, group discussion, simulation etc.) |  |
| **Assessment**  **(**Assessing the learners) | What? The content of the presentation / practice scenarios / feedback ... | The rater was responsible for completing a skills-based checklist that was used to assess the performance of each participant during the drill. All drills were performed in either French or Kinyarwanda, depending on the participant’s preference. The majority of tasks completed during the drill were verbalized, including some detail, while the participant acted as if they were performing the task. By way of example, when performing the task, “obtaining vital signs”, participants would ver- balize the vital sign they were obtaining while pretending to take the “patient’s” vital signs. Completing tasks including intravenous cannula- tion and medication administration involved obtaining the materials, verbalizing actions/intentions, and pretending to perform the task. Foley-catheter placement, bimanual compression, and uterine massage were performed directly on the mannequin while verbalizing what task was being completed and how to perform the task. |
|  | How? The form of the presentation / practice scenarios / feedback ... (e.g., sequencing, scaffolds etc.) | Post-training testing was performed 6–14 days later using the same simulated postpartum hemorrhage drill and skills-based checklist.  Skills were categorized into three performance areas: communication, evaluation, and management. Communication skills included calling for help, requesting anesthesia, notifying nurses of the post- partum hemorrhage, and delegating tasks to nurses. Evaluation skills included obtaining vital signs, inspecting the vagina and cervix for lacer- ations, bimanual uterine inspection, evaluation of the placenta, and requesting laboratory tests. Management skills included placing a Foley catheter, inserting two large-bore intravenous cannulas, adminis- tering uterotonic medications at specific doses and by specific routes, and performing fundal massage and bimanual uterine compression. Total scores were calculated by assessing the degree of completion of each set of skills in the three categories. The maximum communication, evaluation, and management scores were 4, 5, and 12, respectively.  All of the physicians who completed the training, and were present at the study hospital 2 years later, were asked to participate in a second postpartum hemorrhage drill to assess the long-term retention of these skills. The skill-retention simulation drills were conducted using the same scenario as the initial training and the same scoring system was applied. Each participant was assigned a retention score, calcu- lated as the percentage of skills performed correctly in the original post-intervention evaluation that were performed correctly during the re-evaluation. Participants were also asked to complete the same confidence-based survey again. *Pg2* |
|  | Medium? The medium used (on paper, lecture, group discussion, simulation etc.) | Skills were categorized into three performance areas: communication, evaluation, and management. Communication skills included calling for help, requesting anesthesia, notifying nurses of the post- partum hemorrhage, and delegating tasks to nurses. Evaluation skills included obtaining vital signs, inspecting the vagina and cervix for lacer- ations, bimanual uterine inspection, evaluation of the placenta, and requesting laboratory tests. Management skills included placing a Foley catheter, inserting two large-bore intravenous cannulas, adminis- tering uterotonic medications at specific doses and by specific routes, and performing fundal massage and bimanual uterine compression. Total scores were calculated by assessing the degree of completion of each set of skills in the three categories. The maximum communication, evaluation, and management scores were 4, 5, and 12, respectively. |
|  | | |
| **Article # 26 Higgins** | | |
| **Title** | | **Teaching an Experienced Multidisciplinary Team About Postpartum Hemorrhage: Comparison of Two Di erent Methods** |
| **Authors** | | Mary Higgins, Julia Kfouri, Anne Biringer, Gareth Seaward, Rory Windrim |
| **Journal & Publishing information** | | J Obstet Gynaecol Can 2015;37(9):824–828 |
| **Abstract** | | **Objective:** Morbidity from postpartum hemorrhage (PPH) affects 20% of pregnancies worldwide and remains a signi cant cause of maternal mortality. This study compared the impressions of experienced clinicians on the effect of two methods of educational interventions in a MoreOB training program designed to improve recognition and management of PPH.  **Methods:** Participants were exposed to a traditional didactic lecture and an interactive clinical intervention exercise incorporating video simulation of a PPH event with opportunities for feedback and discussion of how to proceed. They were then invited to respond to a questionnaire regarding their impressions of both methods.  **Results:** Of 150 participants, 110 completed the questionnaire. Respondents considered the interactive format to be more effective (55%) and enjoyable (72%) than the traditional didactic format. The majority (81%), however, still recommended a mixture of both interactive and didactic formats in future events, supported by a multidisciplinary drill.  **Conclusion:** Clinical learners value interactivity and mutual reinforcement among varied learning exercises in their educational experiences. Future educational programs may consider incorporating similar methods in order to maximize participants’ receptiveness. |
| **Presentation**  **(**Information presented to learners) | What? The content of the presentation / practice scenarios / feedback ... | The MoreOB course is provided with online modules and yearly in-house updates based on course content.17  As this was a mandatory session for permanent staff, the content was based on the MoreOB module on PPH. |
|  | How? The form of the presentation / practice scenarios / feedback ... (e.g., sequencing, scaffolds etc.) | The first, a traditional didactic lecture, reviewed the information on PPH using the information from the MoreOB online course. The second, an interactive video presentation, brought participants through a typical PPH scenario, with time to allow for discussion at key points and to apply the ndings to the local setting. |
|  | Medium? The medium used (on paper, lecture, group discussion, simulation etc.) |  |
| **Practice**  **(**Practice provided to learners) | What? The content of the presentation / practice scenarios / feedback ... | PPH, their initial management, the involvement of the obstetric, nursing, and anaesthesia teams, and the eventual resolution of the PPH after management in the operating room. |
|  | How? The form of the presentation / practice scenarios / feedback ... (e.g., sequencing, scaffolds etc.) | The scenario then led the participants through the risk factors for PPH, the recognition by the midwifery team of the impending PPH, their initial management, the involvement of the obstetric, nursing, and anaesthesia teams, and the eventual resolution of the PPH after management in the operating room. Options regarding the medical and surgical management of the patient were suggested to participants in order to stimulate discussion. |
|  | Medium? The medium used (on paper, lecture, group discussion, simulation etc.) | At pertinent points in the presentation, videos were shown of key clinicians within the Mount Sinai unit commenting on the management to date of the scenario. These key clinicians included the nursing manager of the case room, a staff neonatologist, the head of the obstetric anaesthesia program, and the head of obstetrics. |
| **Feedback**  **(**Feedback given to learners) | What? The content of the presentation / practice scenarios / feedback ... |  |
|  | How? The form of the presentation / practice scenarios / feedback ... (e.g., sequencing, scaffolds etc.) | The videos concluded with a debriefing of the situation. Other than the comments by key staff members, the videos were filmed using actors, paid from an educational fund used to record common obstetric emergencies and used for training residents and staff. |
|  | Medium? The medium used (on paper, lecture, group discussion, simulation etc.) |  |
| **Assessment**  **(**Assessing the learners) | What? The content of the presentation / practice scenarios / feedback ... |  |
|  | How? The form of the presentation / practice scenarios / feedback ... (e.g., sequencing, scaffolds etc.) |  |
|  | Medium? The medium used (on paper, lecture, group discussion, simulation etc.) |  |
|  | | |
| **Article # 27 Hilton** | | |
| **Title** | | Checklists and multidisciplinary team performance during simulated obstetric hemorrhage |
| **Authors** | | G. Hilton, K. Daniels, S.N. Goldhaber-Fiebert, S. Lipman, B. Carvalho, A. Butwick |
| **Journal & Publishing information** | | International Journal of Obstetric Anesthesia (2015) http://dx.doi.org/10.1016/j.ijoa.2015.08.011 |
| **Abstract** | | **Background:** Checklists can optimize team performance during medical crises. However, there has been limited examination of checklist use during obstetric crises. In this simulation study we exposed multidisciplinary teams to checklist training to evaluate checklist use and team performance during a severe postpartum hemorrhage.  **Methods:** Fourteen multidisciplinary teams participated in a postpartum hemorrhage simulation occurring after vaginal delivery. Before participating, each team received checklist training. The primary study outcome was whether each team used the checklist during the simulation. Secondary outcomes were the times taken to activate our institution-specific massive transfusion protocol and commence red blood cell transfusion, and whether a designated checklist reader was used.  **Results:** The majority of teams (12/14 (86%)) used the checklist. Red blood cell transfusion was administered by all teams. The median [IQR] times taken to activate the massive transfusion protocol and transfuse red blood cells were 5 min 14 s [3:23–6:43] and 14 min 40 s [12:56–17:28], respectively. A designated checklist reader was used by 7/12 (58%) teams that used the checklist. Among teams that used a checklist with versus without a designated reader, we observed no differences in the times to activate the massive transfusion protocol or to commence red blood cell transfusion (P>0.05).  **Conclusions:** Although checklist training was effective in promoting checklist use, multidisciplinary teams varied in their scope of checklist use during a postpartum hemorrhage simulation. Future studies are required to determine whether structured checklist training can result in more standardized checklist use during a postpartum hemorrhage. |
| **Presentation**  **(**Information presented to learners) | What? The content of the presentation / practice scenarios / feedback ... | The teams encountered the same scenario of a patient with atonic PPH fol- lowing vaginal delivery. |
|  | How? The form of the presentation / practice scenarios / feedback ... (e.g., sequencing, scaffolds etc.) | For the design of the checklist the authors reviewed PPH guidelines from recognized obstetric bodies (American College of Obstetricians and Gynecologists (ACOG) and the Royal College of Obstetricians and Gynaecologists), relevant papers from the literature and our institutional PPH protocol.13–15 The final action items for the checklist were selected by consensus by all authors. The checklist was available for participants’ use during simulations (Fig. 1). Fifteen key tasks were identified for inclusion on the PPH checklist which included clinical, communication, and systems-based aspects of management. See Figure 1  Figure 1: Box 1: Uterine Massage, Pitocin, Fluids, Oxygen, Vital sign; Box 2: PPH causes, Bladder, Uterotonics, Call; Box 3: MTP(massive transfusion protocol), IV (intravenous), Stat Labs, Management Plans, Considere OR(operating room), Vasopressors |
|  | Medium? The medium used (on paper, lecture, group discussion, simulation etc.) | All 14 teams underwent standardized educational training immediately before each simulation. Each train- ing session lasted 15 min and comprised two parts. Each simulation was overseen by an expert in obstetric simulation (GH, KD, SL) with at least 10 years of experience in simulation training. The first part was didactic train- ing in which participants viewed a slide presentation with an oral presentation by an expert in obstetric simulation training (GH, SL). During the oral presentation, the benefits of using a checklist were discussed. It was explained to each team that a checklist could be used to initiate tasks (in a prospective manner) or verify task completion (in a retrospective manner). Teams were able to use either approach. Trainers advised teams to designate a participant to be a checklist reader who assisted the team leader by reading out-loud tasks on the check- list and verifying completion of each.9 The second part of the training session was hands-on checklist training. For training purposes, a checklist for managing shoulder dystocia was used which was formatted in a similar style to the PPH checklist. |
| **Practice**  **(**Practice provided to learners) | What? The content of the presentation / practice scenarios / feedback ... | The scenario consisted of a severe PPH (1500 mL blood loss) due to refractory uterine atony in a primiparous 18-year-old patient who had undergone a spontaneous vaginal delivery. The patient became tachycardic and hypotensive consistent with hemorrhagic shock. All simulations were performed on the labor and delivery unit. |
|  | How? The form of the presentation / practice scenarios / feedback ... (e.g., sequencing, scaffolds etc.) | We used a hybrid mannequin (MamaNatalie Birthing Simulator; Laerdal, Wappingers Falls, NY, USA) for simulations in the labor room. A high-fidelity mannequin (NOELLE Maternal and Neonatal Birthing Simulator S550; Gaumard Scientific, Miami, FL, USA) was used for simulations in the OR. All simulations were videotaped. None of the teams received information about the PPH simulation before starting the simulation. |
|  | Medium? The medium used (on paper, lecture, group discussion, simulation etc.) |  |
| **Feedback**  **(**Feedback given to learners) | What? The content of the presentation / practice scenarios / feedback ... | After each simulation all participants were asked to complete a survey related to the use of checklists. Questions required a yes/no answer or an answer based on an ordinal scale (1=poor, 5=excellent). Survey questions are presented in the Appendix. |
|  | How? The form of the presentation / practice scenarios / feedback ... (e.g., sequencing, scaffolds etc.) |  |
|  | Medium? The medium used (on paper, lecture, group discussion, simulation etc.) |  |
| **Assessment**  **(**Assessing the learners) | What? The content of the presentation / practice scenarios / feedback ... | Before each simulation, instructors informed each team that a checklist would be available in the labor room. However, none of the teams received or viewed the actual PPH checklist before the simulation. After being introduced to the scenario, teams were expected to manage ongoing blood loss in a hemodynamically unstable patient. |
|  | How? The form of the presentation / practice scenarios / feedback ... (e.g., sequencing, scaffolds etc.) | Key management tasks were: activation of the institutional massive transfusion protocol (MTP) and transfusion of red blood cells (RBC) via a rapid infuser (Belmont Rapid Infuser; Belmont Instrument Corp., Billerica, MA, USA). The simulation was stopped when each team completed all 15 tasks on the checklist, or when 20 min had elapsed (if <15 tasks were completed).  The primary study outcome was the use of the PPH checklist (yes/no). Secondary outcomes included: times to administer a second-line uterotonic, activate the MTP, and commence RBC transfusion; and the order and number of checklist tasks completed. Time zero for these interventions was taken from the end of the third stage of labor. If teams completed all 15 checklist tasks, the time to complete all tasks was recorded. We also evaluated whether each team used the checklist intermittently or continuously (determined by the study investigator), the location (labor room or OR) when the checklist was first used, and if a checklist reader was used by each team. A reader is a designated person who reads tasks out-loud from the checklist to the team leader. A reader can decrease task saturation experi- enced by the team leader thereby increasing the likelihood that all critical tasks are completed.9 |
|  | Medium? The medium used (on paper, lecture, group discussion, simulation etc.) | During each simulation checklist use was not enforced and the choice to use the checklist was at the discretion of each team. A study investigator not involved in the simulation recorded the times to complete key events. Before each simulation, participants were not informed that key events were timed. These times were verified by a second investigator who reviewed videos of each simulation. |
|  | | |
| **Article # 28 Miller** | | |
| **Title** | | Emergency Birth Hybrid Simulation with Standardized Patients in Midwifery Education: Implementation and Evaluation |
| **Authors** | | Jane Lindsay Miller, Melissa D. Avery, Karin Larson, Anne Woll, Alison VonAchen, Angela Mortenson |
| **Journal & Publishing information** | | JMidwiferyWomensHealth2015;60:298–303 |
| **Abstract** | | Structured simulations have become a critical part of health professions education at every level, particularly for high-risk, low-incidence sce- narios. This article describes the implementation and evaluation of a hybrid simulation of emergency birth situations in a graduate midwifery program. In the fall of 2011 and spring of 2012, nurse-midwifery students twice participated in 2 simulated emergencies—shoulder dystocia and postpartum hemorrhage—using hybrid simulation (a standardized patient paired with a birth task trainer). Students found the simulations to be realistic. The use of best practices (ie, repetitive practice, team learning, small group debriefing, and large group debriefing) enhanced the quality of the simulation experience and the learners’ reflection about their professional skills, strengths, weaknesses, and confidence in managing these 2 obstetric emergencies. This article is part of a special series of articles that address midwifery innovations in clinical practice, education, interprofessional collaboration, health policy, and global health. |
| **Presentation**  **(**Information presented to learners) | What? The content of the presentation / practice scenarios / feedback ... | The postpartum hemorrhage and shoulder dystocia simulations were designed by nurse-midwifery faculty and simulation professionals for formative assessment of required skills. The collaboration between the faculty and the simulation professionals resulted in heightened clinical realism and authenticity of the standardized patients’ performances, improved focus on desired competencies, and ensured that the simulations could be repeated and standardized. Case 1 was a healthy primigravida with labor dystocia who was in the transition phase of labor (Figure 1). In the first scenario, students were given a report, then they met and as- sessed the woman’s needs and provided her with labor support. In the second scenario, the woman gave birth using the MamaNatalie® birth simulator. Red food coloring was added to water to simulate blood. Students managed the birth and subsequent postpartum hemorrhage. |
|  | How? The form of the presentation / practice scenarios / feedback ... (e.g., sequencing, scaffolds etc.) | The students in this course participated in the 2 simulations twice, once in the fall of 2011 and again in the spring of 2012. The same students completed both sets of simulations. By the time the students participated in the first simulation experi- ence in the fall of 2011, most had observed or anticipated a shoulder dystocia and postpartum hemorrhage at least once.  The simulation experience began with a prebriefing to provide logistical information (eg, orienting students to the clinical simulation space, equipment), as well as their roles and expectations, which were the ground rules of the simulation. Orientation to the simulation scenario included a description of the simulation process, an opportunity to experience the equipment and the space, and instructions for successful sce- nario completion. |
|  | Medium? The medium used (on paper, lecture, group discussion, simulation etc.) | Each case was designed with 1 to 2 specific risk factors present so the students had the opportunity to anticipate the  potential emergency. The cases were each 30 minutes in du- ration and divided into two 15-minute scenarios (Figure 1). |
| **Practice**  **(**Practice provided to learners) | What? The content of the presentation / practice scenarios / feedback ... | The case scenarios were constructed so that the students would perform a risk assessment while they either reviewed a prenatal record or performed an admission history and physical examination. A simulation confederate (an individual role-playing a midwife ending her call period or a registered nurse admitting the woman in labor) provided assessment findings and a verbal report. *Pg299*  Based on the first simulation experiences with the 2 cases, faculty made revisions utilizing student evaluation informa- tion. Increased complexity was added to challenge the same students in their second experience participating in these 2 simulation cases in the following semester in March 2012. For example, care of a depressed newborn was added, requiring the student to make decisions about resuscitation and to in- teract with a woman who appeared to be frightened. *Pg300* |
|  | How? The form of the presentation / practice scenarios / feedback ... (e.g., sequencing, scaffolds etc.) | Students then experienced their first 15-minute interaction with the standardized patient to provide labor support, clarify history, and develop a plan for the birth. Following the first interaction, the students had a 5-minute mini-huddle that was facilitated by faculty. During the mini-huddle, simulation rooms were reset by staff. The second 15-minute scenario unfolded with the birth and either a postpartum hemorrhage or a shoulder dystocia. After each team completed both cases, the teams reconvened for a large group debriefing. |
|  | Medium? The medium used (on paper, lecture, group discussion, simulation etc.) | A team of 4 students participated in each simulation case. The following 4 roles were assigned: lead the team as a midwife, participate as a registered nurse caring for the patient, participate as a consulting midwife, and observe and record the events of the birth in the woman’s health record. Our intention was that the lead midwife would manage the case and direct the others, with the opportunity to ask questions of the consultant midwife as needed. After allowing the lead midwife time to act, the consultant was encouraged to ask questions or make suggestions to enhance the care. One student who had the role of registered nurse provided the oppor- tunity to collaborate in managing the case and also provided perspective on role transition from nurse to midwife. |
| **Feedback**  **(**Feedback given to learners) | What? The content of the presentation / practice scenarios / feedback ... |  |
|  | How? The form of the presentation / practice scenarios / feedback ... (e.g., sequencing, scaffolds etc.) | Following the simulation, a 30-minute debriefing was facilitated by the simulation center director using advocacy- inquiry methodology.25 Student responses were recorded in real time on a white board as a means for clarification and information sharing. Students were guided through a process of acknowledging their emotional responses, identifying key points of clinical decision making and management, and reflecting on the strengths and weaknesses of individual and team performance. Following the debriefing, a written evaluation was completed by the students for both quantitative and qualitative feedback. |
|  | Medium? The medium used (on paper, lecture, group discussion, simulation etc.) |  |
| **Assessment**  **(**Assessing the learners) | What? The content of the presentation / practice scenarios / feedback ... | The core determinants included 5 domains: 1) simulation logistics, 2) clinical relevance and accuracy of each case, 3) realism of each case, 4) usefulness of the simulations, and 5) global impressions of the simulation experience.26 |
|  | How? The form of the presentation / practice scenarios / feedback ... (e.g., sequencing, scaffolds etc.) | The evaluation survey followed a format used routinely with all simulations in the Academic Health Center Simulation Center (Table 2). The specific items in the tool were adapted to the details of the birth simulations and asked for both positive and negative feedback on key components of the simulation. |
|  | Medium? The medium used (on paper, lecture, group discussion, simulation etc.) | Fourteen quantitative items using a 5-point Likert scale (including a null-value option of “don’t know”) were included.  In addition, we asked students 3 open-ended questions: 1) to identify the best 3 things about the experience, 2) to recommend 3 changes based on perceived shortcomings, and 3) if there was anything else they learned from the experience. Qualitative comments were reviewed using content analysis.27 |

1. **Articles' Tables PPH Simulation & ID Subset29to32**

| **Article # 29 Wong** | | |
| --- | --- | --- |
| **Title** | | The state of Illinois obstetric hemorrhage project: pre-project and post-training examination scores |
| **Authors** | | Cynthia A. Wong, Shirley Scott, Robin L. Jones, Jennifer Walzer, and Stacie Geller; For the members of the State of Illinois Maternal Mortality Review Committee |
| **Journal & Publishing information** | | J Matern Fetal Neonatal Med, Early Online: 1–5 2015 |
| **Abstract** | | **Objective:** The Illinois Department of Public Health mandated that all clinicians who provide care to obstetric patients participate in the Illinois Obstetric Hemorrhage Project. The aim of the current report is to describe change in knowledge among providers engaged in the project, as assessed by pre- and post-tests.  **Methods:** The project, implemented 2008 to 2010, included four components: a written 25-item multiple-choice examination (pre-test), a didactic lecture, skill stations (for teaching blood loss estimation), and a simulation drill and debriefing. Participants completed a post-test 6 months later. Pre- and post-test examination scores were compared.  **Results:** Data from 95 hospitals are included in this analysis (9456 paired test results). The proportion of participants who scored 88% correct answers increased from 10.9% on the pre-test to 49.1% on the post-test (p50.0001). Registered nurses made greater improvements in test scores than anesthesia and obstetric providers (p50.0001).  **Conclusions:** The Illinois Obstetric Hemorrhage Project was successful in improving knowledge of obstetric hemorrhage in a large number of providers with different expertise and experience levels. Further long-term study is essential to determine whether the skills acquired during the Project contribute to improved obstetric hemorrhage outcomes for the women of Illinois. |
| **Presentation**  **(**Information presented to learners) | What? The content of the presentation / practice scenarios / feedback ... | The goal of the program was to improve (1) risk assessment and preparation for possible hemorrhage, (2) estimation of blood loss skills, and (3) recognition and treatment of hemorrhage and hypovolemia. |
|  | How? The form of the presentation / practice scenarios / feedback ... (e.g., sequencing, scaffolds etc.) |  |
|  | Medium? The medium used (on paper, lecture, group discussion, simulation etc.) |  |
| **Practice**  **(**Practice provided to learners) | What? The content of the presentation / practice scenarios / feedback ... | Topics covered in the didactic presentation included case presentations, cardiovascular physiologic changes of pregnancy and the purpureum, recognition of obstetric hemor-rhage and accurate estimation of blood loss (including visual estimation and gravimetric methods), fluid management, indications for uterotonic medications, an overview of procedures used to treat hemorrhage (e.g. B-Lynch technique, uterine artery ligation or embolization), and the importance of the team approach to management. |
|  | How? The form of the presentation / practice scenarios / feedback ... (e.g., sequencing, scaffolds etc.) | The program included four components and was designed to be completed in a 4-h time frame: a written benchmark examination (pre-test) (30 min), a didactic lecture (90 min), skill stations (30 min), and a simulation drill (30 min) followed by debriefing (60 min). |
|  | Medium? The medium used (on paper, lecture, group discussion, simulation etc.) | The program’s educational material, including didactic material, instructions for setting up the skill stations, and simulations, is available through the Illinois Department of Public Health website (www.dph.illinois.gov).  The program was administered through Illinois’s Perinatal Network system. Each hospital was asked to identify physician and nurse ‘‘champions’’ to lead the project in each institution. Two ‘‘train-the-trainer’’ sessions were held to review the instructional material. The content of the didactic lecture was standardized; however, the presenter and format varied among institutions, and included grand rounds, video- tape and web-based presentations. Not all institutions completed the required material in a single 4-h session.  The skill stations were developed to teach accurate visual estimation of blood loss using materials commonly used to absorb blood during childbirth, including perineal pads, disposable underpads, laparotomy sponges and 4 4 inch gauze squares. The 10 skill stations were prepared using predetermined volumes of imitation blood and clots. Participants were asked to view the stations and estimate blood loss, and were then told the actual blood loss for each station. Finally, standardized simulation cases were prepared by the MMRC workgroup, along with instructions and assessment checklists for the simulation drills. Drills were led by the team leaders in the individual institutions. *Pg2* |
| **Feedback**  **(**Feedback given to learners) | What? The content of the presentation / practice scenarios / feedback ... |  |
|  | How? The form of the presentation / practice scenarios / feedback ... (e.g., sequencing, scaffolds etc.) | The participants were informed of their scores (pretest), but were not allowed to keep a copy of the test. Providers who practiced at more than one institution were required to complete the pre-test, attend the didactic lecture and review the skills stations once. They were required to complete the simulation drill at each hospital in which they provided services. |
|  | Medium? The medium used (on paper, lecture, group discussion, simulation etc.) |  |
| **Assessment**  **(**Assessing the learners) | What? The content of the presentation / practice scenarios / feedback ... | The purpose of the written pre-test was to assess partici- pants’ baseline knowledge before the education intervention. |
|  | How? The form of the presentation / practice scenarios / feedback ... (e.g., sequencing, scaffolds etc.) | Training for individual providers consisted of a pre-test, training in the recognition and treatment of obstetric hemorrhage, and 6 months later, a post-test.  The purpose of the written pre-test was to assess participants’ baseline knowledge before the education intervention. A draft of the written test was reviewed for face validity by a group of subject matter experts consisting of 10 maternal– fetal medicine specialist co-directors of perinatal centers with current clinical activity on labor and delivery units and 10 perinatal administrators. Unclear or confusing questions were rewritten. Members of the MMRC assigned the test questions to one of three categories: risk and assessment, diagnosis, and management. The final examination consisted of 25 multiple-choice questions with one correct answer and three distractors. The same test (post-test) was repeated 6 months after the completion of the program. Each hospital was given the option to use a web-based or classroom format to administer the test. |
|  | Medium? The medium used (on paper, lecture, group discussion, simulation etc.) | Each hospital was responsible for administering the test in a format that did not compromise the integrity of the questions. It was the hospital’s responsibility to ensure that the participants took the test prior to participating in the didactic, skill stations and simulation components. The hospital was also charged with keeping a record of the individual pre-test and post-test scores. The examinations were graded locally, reported on an Excel spreadsheet, and sent to the perinatal centers. |
|  | | |
| **Article # 30 Evans** | | |
| **Title** | | Competency-based training “Helping Mothers Survive: Bleeding after Birth” for providers from central and remote facilities in three countries |
| **Authors** | | Cherrie L. Evans, Peter Johnson, Eva Bazant, Neeta Bhatnagar, Jane Zgambo, Asma R. Khamis |
| **Journal & Publishing information** | | C.L. Evans et al. / International Journal of Gynecology and Obstetrics 126 (2014) 286–290 |
| **Abstract** | | **Objective:** To validate a new training module for skilled and semiskilled birth attendants authorized to provide care at birth—Helping Mothers Survive: Bleeding After Birth (HMS:BAB)—aimed at reducing postpartum hemorrhage, the leading cause of maternal mortality worldwide. BAB training involves single-day, facility-based training that emphasizes simulation of scenarios related to prevention, detection, and management of postpartum hemorrhage. **Methods:** A total of 155 skilled and semiskilled birth attendants participated in training in India, Malawi, and Zanzibar, Tanzania. Knowledge and confidence were assessed before and after training. Skills and acceptability were assessed after training.  **Results:** Knowledge and confidence scores improved significantly from pre- to post-training among all cadres in all three countries. The proportion of providers with passing knowledge scores increased significantly from pre- to post-training among all cadres except for those already high at baseline. On three post-training skills tests the overall proportion of individuals with a passing score ranged from 83% to 89%.  **Conclusion:** BAB training in prevention and management of postpartum hemorrhage increased knowledge and confidence among skilled and semiskilled birth attendants. Further studies are needed to determine the impact of this training on skills retention and clinical outcomes following postpartum hemorrhage, after broader implementation of the training program. |
| **Presentation**  **(**Information presented to learners) | What? The content of the presentation / practice scenarios / feedback ... | The BAB module was designed to teach birth attendants to prevent postpartum hemorrhage using international standards and a simplified protocol for management of postpartum hemorrahge |
|  | How? The form of the presentation / practice scenarios / feedback ... (e.g., sequencing, scaffolds etc.) | The training consists of a single-day, facility-based training emphasizing simulation of scenarios relevant to prevention, detection, and management of postpartum hemorrhage. |
|  | Medium? The medium used (on paper, lecture, group discussion, simulation etc.) |  |
| **Practice**  **(**Practice provided to learners) | What? The content of the presentation / practice scenarios / feedback ... | The materials, methods, and assessment tools of BAB were reviewed by the International Federation of Gynecology and Obstetrics (FIGO), the International Confederation of Midwives, the United Nations Population Fund, the World Health Organization, the Maternal and Child Health Integrated Program, the American College of Obstetricians and Gynecologists, the American College of Nurse-Midwives, and the American Academy of Pediatrics. The training module and accompa- nying knowledge and skills assessment were finalized and approved on October 21, 2011, at a meeting in Washington, D.C., that involved representatives from the respective organizations. |
|  | How? The form of the presentation / practice scenarios / feedback ... (e.g., sequencing, scaffolds etc.) | Twelve experienced maternal and newborn health trainers from the intervention countries were oriented to the BAB module, simulators, skills, assessment, standardization, and study procedures in a two-day session conducted by the principal investigator (CLE).  During all sessions there were no more than six learners to each trainer during  hands on practice. |
|  | Medium? The medium used (on paper, lecture, group discussion, simulation etc.) | Materials include a graphic flipbook for training, job aid for supporting clinical decisions, and handbook for facilitation and learning. An economical apron-style simulator, consisting of an abdominal “skin” containing a uterus holding a fetal mannequin, postpartum size uterus, and blood tank, was used to simulate birth, normal bleeding, and postpartum hemorrhage. |
| **Feedback**  **(**Feedback given to learners) | What? The content of the presentation / practice scenarios / feedback ... |  |
|  | How? The form of the presentation / practice scenarios / feedback ... (e.g., sequencing, scaffolds etc.) | Trainers were both participants and data collectors. Their participant role was to provide feedback on the module from a trainer perspective. |
|  | Medium? The medium used (on paper, lecture, group discussion, simulation etc.) |  |
| **Assessment**  **(**Assessing the learners) | What? The content of the presentation / practice scenarios / feedback ... | The present stu Skills were assessed using three Objective Structured Clinical Examinations (OSCEs). The three OSCEs assessed prevention of postpartum hemorrhage, management of retained placenta, and management of severe hemorrhage from uterine atony. OSCEs were conducted using simulators immediately after training. dy focused on two levels of Kirkpatrick model of evaluation of training: participant reaction and participant learning. *Pg287* |
|  | How? The form of the presentation / practice scenarios / feedback ... (e.g., sequencing, scaffolds etc.) | We measured change in four aspects of learning: BAB relevant knowledge, self-reported confidence on five clinical skills, actual clinical skills post-training, and acceptability of training materials and methods. Trainers were both participants and data collectors. Their participant role was to provide feedback on the module from a trainer perspective.  Knowledge and confidence were assessed using two pre- and post- training oral questionnaires: 26 items for knowledge and five items for confidence. These were administered orally by trainers in the local language to eliminate bias due to variations in literacy among providers. Participants rated their confidence in providing AMTSL, treating post- partum hemorrhage, accessing advanced care, assessing the placenta, and bimanual uterine compression. |
|  | Medium? The medium used (on paper, lecture, group discussion, simulation etc.) | Skills were assessed using three Objective Structured Clinical Examinations (OSCEs). The three OSCEs assessed prevention of postpartum hemorrhage, management of retained placenta, and management of severe hemorrhage from uterine atony. OSCEs were conducted using simulators immediately after training. A criterion reference pass score was developed for all assessments using the Angoff procedure, with input from global organizations that reviewed the module [17].  Acceptability of materials, methods, and specific aspects of BAB was assessed by participant evaluation questionnaires using a five-point Likert type scale (1 = “disagree” to 5 = “agree”). *Pg287* |
|  | | |
| **Article # 31 Monod** | | |
| **Title** | | Optimization of competency in obstetrical emergencies: a role for simulation training |
| **Authors** | | Cécile Monod, Cora A.Voekt, Martina Gisin, Stefan Gisin, Irene M. Hoesli |
| **Journal & Publishing information** | | Arch Gynecol Obstet (2014) 289:733–738 DOI 10.1007/s00404-013-3111-6 |
| **Abstract** | | Purpose In obstetrical emergency situations, optimal management requires the immediate coordinated actions of a multi-disciplinary and multi-professional team. This study investigated the influence of simulation training on four specific skills: self-confidence, handling of emergency sit- uation, knowledge of algorithms and team communication. Methods Clinical algorithms were first presented to the participants. Training for six emergency situations (shoul- der dystocia, postpartum haemorrhage, pre-eclampsia, maternal basic life support, neonatal resuscitation and operative vaginal birth) was performed using high- and low-fidelity simulation mannequins. General impression of the simulation training and the four above-mentioned skills were evaluated anonymously through a self-assessment questionnaire with a five-point Likert scale immediately after the training and 3 months later.  Results From November 2010 to March 2012, 168 par- ticipants, distributed over six one-day courses, took part in the training. 156 participants returned the questionnaire directly after the course (92.9 %). The questionnaire return rate after 3 months was 36.3 %. The participants gave higher Likert scale answers for the questions on the four specific skills after 3 months compared to immediately after the course. The improvement was statistically sig- nificant (p B 0.05) except for the question regarding team communication.  Conclusion Implementation of simulation training strengthens the professional competency. |
| **Presentation**  **(**Information presented to learners) | What? The content of the presentation / practice scenarios / feedback ... | Midwifes and obstetricians from different public and private Swiss hospitals were allowed to participate in these courses. After a brief presentation of the clinical algorithms and essentials of medical simulation and crisis resource management (CRM), the participants trained in small groups and retained their normal function (junior or senior obstetrician, midwife) under supervision of multi-professional tutor-teams in six different obstetrical emergency situations: shoulder dystocia, postpartal haemorrhage, instrumental delivery for foetal distress, pre-eclampsia/eclampsia, maternal Basic Life Support and neonatal resuscitation. |
|  | How? The form of the presentation / practice scenarios / feedback ... (e.g., sequencing, scaffolds etc.) |  |
|  | Medium? The medium used (on paper, lecture, group discussion, simulation etc.) |  |
| **Practice**  **(**Practice provided to learners) | What? The content of the presentation / practice scenarios / feedback ... | Midwifes and obstetricians from different public and private Swiss hospitals were allowed to participate in these courses. After a brief presentation of the clinical algorithms and essentials of medical simulation and crisis resource management (CRM), the participants trained in small groups and retained their normal function (junior or senior obstetrician, midwife) under supervision of multi-professional tutor-teams in six different obstetrical emergency situations: shoulder dystocia, postpartal haemorrhage, instrumental delivery for foetal distress, pre-eclampsia/eclampsia, maternal Basic Life Support and neonatal resuscitation. |
|  | How? The form of the presentation / practice scenarios / feedback ... (e.g., sequencing, scaffolds etc.) | The participants worked on different simple and high-fidelity mannequins: Noelle®(Gaumard, Miami, FL, USA) for shoulder dystocia training, Ambu®Man (Ambu, Ballerup, Denmark) for maternal resuscitation, SimMan®Classic and 3G (Laerdal Medical, Stavanger, Norway) for postpartal haemorrhage and pre-eclampsia/eclampsia and obstetrical leather models for operative vaginal delivery.  The training groups of participants consisted of a maximum of six participants, namely, two midwives, two junior and two senior obstetricians. Three of them (one of each function) actively participated in the clinical scenario, while the remaining three were observants. |
|  | Medium? The medium used (on paper, lecture, group discussion, simulation etc.) | The courses were conducted at the Swiss Center for Medical Simulation (‘‘SimBa’’), Basel, Switzerland.  To further augment the realism of the emergency situations and allow training of communication with the patient, scripted role players were acted as standardized patients at all the training stations. |
| **Feedback**  **(**Feedback given to learners) | What? The content of the presentation / practice scenarios / feedback ... |  |
|  | How? The form of the presentation / practice scenarios / feedback ... (e.g., sequencing, scaffolds etc.) | With the consent of the participants, the training was videotaped at three of the six stations (pre-eclampsia/eclampsia, postpartal haemorrhage and shoulder dystocia). The tutors were comprised experienced obstetricians, anaesthesiologists and neonatologists with special education in leading simulation training courses. After each scenario, a debriefing was held to give immediate feedback to the participants and their performance. Where applicable, relevant extracts from the video-recordings were shown to strengthen the learning experience of the teams. The video records were erased after the course. |
|  | Medium? The medium used (on paper, lecture, group discussion, simulation etc.) |  |
| **Assessment**  **(**Assessing the learners) | What? The content of the presentation / practice scenarios / feedback ... | Immediately (questionnaire a) and 3 months after training (questionnaire b) the participants filled up an anonymous self-assessment questionnaire with a five-point Likert scale, evaluating subjective changes in the following skills: self-confidence, handling of the obstetrical emer- gency situation, knowledge of algorithms and team communication. The questions of our questionnaires a and b are not published in any other study. At the same time, the participants also answered a six-point Likert scale questionnaire on general acceptance of simulation training, a questionnaire previously used in a study by Blum et al. [9], together with the professional experience and function of each participant. The voluntary and anonymous questionnaires were a standard part of assessment of the institu- tional quality of service and, therefore, ethical approval was not necessary.  The first written questionnaire was filled up directly after the course and the second electronically in Survey- MonkeyÒ 3 months after the course. |
|  | How? The form of the presentation / practice scenarios / feedback ... (e.g., sequencing, scaffolds etc.) | Immediately (questionnaire a) and 3 months after training (questionnaire b) the participants filled up an anonymous self-assessment questionnaire with a five-point Likert scale, evaluating subjective changes in the following skills: self-confidence, handling of the obstetrical emergency situation, knowledge of algorithms and team communication. The questions of our questionnaires a and b are not published in any other study. At the same time, the participants also answered a six-point Likert scale questionnaire on general acceptance of simulation training, a questionnaire previously used in a study by Blum et al. [9], together with the professional experience and function of each participant. The voluntary and anonymous questionnaires were a standard part of assessment of the institutional quality of service and, therefore, ethical approval was not necessary. |
|  | Medium? The medium used (on paper, lecture, group discussion, simulation etc.) | The first written questionnaire was filled up directly after the course and the second electronically in Survey- MonkeyÒ 3 months after the course. |
|  | | |
| **Article # 32 Highfield** | | |
| **Title** | | Effect of Nurse-Led Simulation on OB/Perinatal Nurses' Knowledge & Confidence in Managing Complications & Emergencies |
| **Authors** | | Martha E. Farrar Highfield PhD RN |
| **Journal & Publishing information** | |  |
| **Abstract** | | **Abstract:** Simulation may help both novice and experienced clinicians maintain competence in managing high-risk, low-frequency obstetric and perinatal complications and emergencies. erefore, we designed a pre-/posttest study to determine whether a day of nurse-led lecture plus low- delity simulation would increase registered nurses’ self-assessed knowledge and con dence in managing ve high-risk obstetric/perina- tal situations. e Nursing Management of OB/Perinatal Complications & Emergencies (NursOB) scale was distributed to 67 labor/birth and postpartum nurses before and a er a simulation training day. Preliminary ndings supported validity and reliability of the NursOB scale, but nurses’ knowledge and con dence did not improve a er the simulation (*p* < .05). Anecdotally, nurses’ interest in competence reviews was reinvigorated, and we gained practical knowledge in simulation delivery. Future simulations could enhance outcome measures, improve drills, and establish criterion- related validity of the NursOB scale. More research is warranted. |
| **Presentation**  **(**Information presented to learners) | What? The content of the presentation / practice scenarios / feedback ... | Our intent was to prepare RNs so that they could more promptly treat women with unstable conditions who may have already experienced a rst delay in seeking care and a second delay in traveling to care (Pacagnella, Cecatti, Osis, & Souza, 2012). |
|  | How? The form of the presentation / practice scenarios / feedback ... (e.g., sequencing, scaffolds etc.) | We planned a 1-day, low- fidelity, nurse-led simulation exercise with task trainers and role-playing supervisors to replace the prior annual competence training of lectures with case discussions. Our intent was to prepare RNs so that they could more promptly treat women with unstable conditions who may have already experienced a first delay in seeking care and a second delay in traveling to care (Pacagnella, Cecatti, Osis, & Souza, 2012) |
|  | Medium? The medium used (on paper, lecture, group discussion, simulation etc.) |  |
| **Practice**  **(**Practice provided to learners) | What? The content of the presentation / practice scenarios / feedback ... | We also included the following recommended, evidence-based simulation elements: (a) outcome measures were aligned with organizational perinatal safety goals, (b) the hospital’s parent organization supported obstetric/perinatal simulation with materials and policies, (c) the first-line RN manager and supervisors were actively engaged in simulation delivery, and (d) adequate human, equipment, and time resources were committed in advance (Auguste et al., 2012). |
|  | How? The form of the presentation / practice scenarios / feedback ... (e.g., sequencing, scaffolds etc.) | We scheduled the simulation day and o ered a presimulation review lecture to reduce participant anxiety and promote success (Argani et al., 2012; Sørensen et al., 2014; Sweeney et al., 2015).  Design characteristics: Scheduled simulation days included prescenario review and postscenario debriefing. The objective was to practice hands-on skills that resulted in demonstration of clinical competence. Low- fidelity equipment and nursing-only simulation were used as economical and feasible options for clinical ex- perts new to simulation teaching. Problem solving was encouraged through unpredictable scenario progress and verbal and cue card prompts (e.g., vital signs) during scenarios.  Thee educational intervention then began immediately with 4 hours of morning lecture/discussion, followed by a lunch break and 4 hours of a ernoon low-fidelity simulations. Attendees received hand-outs of the day’s agenda, a hospital skills validation checklist, and lecture slides. They earned 8 continuing nursing education contact hours at day’s end.  An external educational vendor with experience in low- delity obstetric/perinatal simulation had been hired to consult with the manager, supervisors, and an in-house sta develop- ment specialist to design the day within the context of hospital policies, procedures, and resources. A di erent vendor educa- tor taught each of the three morning classes and then assisted with afternoon simulation. All external educators were experienced in obstetric/perinatal practice and teaching, and they used standardized PowerPoint slides and content (see Box 2).  *BOX 2 content transcribed:* 1)Review of intrapartum and postpartum emergencies: Shoulder dystocia;  Cord prolapse;  Uterine inversion ; Postpartum hemorrhage 2) Review of general obstetric emergencies: Amniotic embolism/pulmonary embolism/ cardiac arrest in pregnancy, Severe pregnancy-induced hypertension and seizure management, Malignant hyperthermia  Learners were all experienced labor/birth or postpartum RNs; therefore, the lecture/discussion was a review of nursing management of complications and emergencies. Prescenario knowledge has been identi ed as an important element of simulation e orts (Chichester, Hall, Wyatt, & Pomilla, 2014); therefore, the lecture/discussion promoted a more homogene- ous knowledge base among labor/birth and postpartum nurse learners. Interactive discussion of cases illustrated key points and how complications might progress.  Afternoon simulation included five different standardized stations through which all RN attendees rotated in groups of ve to six. Stations used task trainers, supervisor role playing, and a standardized, postscenario, hospital-designed verbal and written debrie ng (see Box 3), with additional station-specific questions, such as *What kind of injuries to both mom and baby can you expect from a shoulder dystocia emergency?* Scenarios were oxytocin management, operative vaginal birth, postpar- tum hemorrhage, shoulder dystocia, and pregnancy-induced hypertension (see Box 4 for example). Verbal cues or written cue cards with vital signs or other information communicated “patient” deterioration or improvement.  All five stations were led by RNs, who facilitated, coached, evaluated performance, and debriefed learners. The external educators conducted the postpartum hemorrhage station each day, including videotaping of RN performance that was used in debrie ng and then deleted. Other stations were led by those with limited experience in simulation: the obstetric/perinatal nurse manager and three obstetric/perinatal supervisors. |
|  | Medium? The medium used (on paper, lecture, group discussion, simulation etc.) | Teachers: The unit manager and supervisors, who were in-house simulation leaders for this project, had prior experience with paper-and-pencil case simulations only. They worked collaboratively with each other and in consultation with an external educational vendor with low-fidelity simulation experience to plan scenarios in the context of our setting’s policies, procedures, and resources.  Because the hospital had no simulation laboratory, class and simulations were conducted in a large hospital classroom. Beds, intravenous equipment, carts, and other supplies were moved into and out of the room for each of the 3 simulation days. |
| **Feedback**  **(**Feedback given to learners) | What? The content of the presentation / practice scenarios / feedback ... | **BOX 3** Sample Perinatal High-Risk Simulation Debriefing Form: Start debriefing with *How do you feel the simulation went? What do you think went well? What do you think you could improve upon?* |
|  | How? The form of the presentation / practice scenarios / feedback ... (e.g., sequencing, scaffolds etc.) | Educational practices: Learner expectations were available presimulation as written verbal and performance competencies. Postpartum and labor/birth RNs were assigned together to scenarios so that they could learn from each other. Ongoing coaching occurred. |
|  | Medium? The medium used (on paper, lecture, group discussion, simulation etc.) |  |
| **Assessment**  **(**Assessing the learners) | What? The content of the presentation / practice scenarios / feedback ... | Our simulation elements reflected the simulation model by Je ries (2005; see Box 1), and we measured perceived knowledge and confidence before and after the simulation exercise.  Educational practices: Learner expectations were available presimulation as written verbal and performance competencies. Postpartum and labor/birth RNs were assigned together to scenarios so that they could learn from each other. Ongoing coaching occurred.  Learner outcomes: The study evaluated self-reported knowledge and confidence gains. *Confidence* was un- derstood as beliefs about how well one is able to perform skills rather than about having skills, per se, and *knowledge* was defined as learned practice-related information that supports skill performance (Bandura, 1989; O’Donnell, Decker, Howard, Levett-Jones, & Miller, 2014). For educational—not research—purposes, the simulation leaders measured performance via evaluator checklist and satisfaction as part of debriefing. Critical thinking was not discretely measured.  Using the NursOB scale, RNs could self-report on a scale from 0 to 10 their knowledge and confidence in dealing with each of the ve priority complications/emergencies. Each item on the scale was scored individually from 0 to 10, and then the five knowledge items and the five confidence items were totaled for two subscale scores ranging from 0 to 50. Higher scores suggested higher perceived knowledge and confidence. |
|  | How? The form of the presentation / practice scenarios / feedback ... (e.g., sequencing, scaffolds etc.) | Outcomes were quantitatively measured, and we chose low- fidelity, nurse-only simulation (Ennen & Satin, 2010) as a step toward more complex, interprofessional drills.  Because no targeted tool was available to measure RNs’ knowledge and cofidence of our organiza tion’s annual perinatal safety priorities, we designed a self-reporting tool entitled Nursing Management of OB/Perinatal Complications & Emergencies (NursOB) scale (see Figure 1).  Content validity was established using a panel of two obstetric/perinatal RN experts—a doctoral candidate with more than 20 years of obstetric clinical experience and a masters-prepared sta educator with more than 30 years of obstetric clinical practice who was also a certi ed obstetric nurse and childbirth instructor. These experts independently reviewed the scale’s items and through an iterative process selected ve of our parent organization’s perinatal safety priorities that were responsive to RN management and amenable to simulation.  The NursOB scale was piloted with two bachelor of science in nursing (BSN) degree students only to verify understandability and completion time. e students found the tool clear, and their completion time was less than 1 to 2 minutes. We assumed that if students found the NursOB scale clear and easy to complete quickly, then practicing RNs would as well. Students were not included as participants or experts.  No interrater reliability was established for in-house simula- tion leaders, because the same person led and debriefed the same simulation each day (e.g., the manager conducted shoulder dystocia all 3 days). Intrarater consistency was supported by using the same case study, a case-specific performance evaluation tool, and a standardized debriefing tool. For hospital competency records, each station leader completed a pass/fail performance evaluation of each RN (see Box 5 for composite sample). |
|  | Medium? The medium used (on paper, lecture, group discussion, simulation etc.) | Two weeks after the final training day, the nurse manager and supervisors distributed the postsimulation NursOB, along with cover information to all obstetric/perinatal RNs during unit huddles and via a folder on the unit that was available 24 hours a day, 7 days a week for 3 months. A $2 coffee gift card was given to RNs who reported submitting a scale. |

1. **Rating Instrument PPH Simulation & ID Subset1to7^*1^_SORENSEN^*2^ (example of rating instrument)**

*1 – each article’s table subset varied according to the subset each rater was allocated to; *2 each rater received their own rating instrument adjusted to the subset he/she was allocated to.

**Instructional Design & PPH Simulation - Maastricht University**

**RATING SCALE - Subset Articles 1 to 7**

Dear Rater SORENSEN,

You are receiving your rating instrument for the analysis of your subset of seven articles' tables. The tables with the singled out simulation based training programs, which you will be using to rate, also follows attached in this email in other word document entitled " Articles' Tables PPH Simulation & ID Subset1to7 ".

In the rating scale below, please note that the columns correspond to the articles' number & authors' names and the rows contain the items, divided into 5 subscales, which you will be making your judgment. We will use a **5-point Likert scale** (**1 = strongly disagree; 2= disagree; 3 = neutral; 4= agree; 5 = strongly agree).** We ask you to please type your answer in the corresponding cell and in the cases you judge a specific item is not possible to be judged, please type **ND (not described)**. When you are finished, please save this word document with the typed answers and send them back to me by email.

|  | | **Article # 1 Andriguetti** | **Article # 2 Birch** | **Article # 3 Chichester** | **Article # 4 Clark** | **Article # 5 Cooper** | **Article # 6 Scholes** | **Article # 7 Deering** |
| --- | --- | --- | --- | --- | --- | --- | --- | --- |
| **Example item:** This article you received describes a PPH simulation training. | | 5 | 5 | 5 | 5 | 5 | 5 | 5 |
| **Subscale 1) Authenticity** | | **Article # 1 Andriguetti** | **Article # 2 Birch** | **Article # 3 Chichester** | **Article # 4 Clark** | **Article # 5 Cooper** | **Article # 6 Scholes** | **Article # 7 Deering** |
| a) | Scenarios are based on real-life tasks |  |  |  |  |  |  |  |
| b) | Trainees receive relevant theoretical information before they start to work on the scenario(s) |  |  |  |  |  |  |  |
| c) | Trainees receive guidance while they are working on the scenario(s) |  |  |  |  |  |  |  |
| **Attention:** Is there more than one scenario described in the article? Please, only if your answer to this prior question was yes, you should answer the following questions. | |  |  |  |  |  |  |  |
| d) | Scenarios differ from each other on the same dimensions as real-life tasks |  |  |  |  |  |  |  |
| e) | Scenarios are sequenced from simple to complex |  |  |  |  |  |  |  |
| f) | Trainees are stimulated to compare and contrast scenarios |  |  |  |  |  |  |  |
| **Subscale 2) Activation of prior knowledge** | | **Article # 1 Andriguetti** | **Article # 2 Birch** | **Article # 3 Chichester** | **Article # 4 Clark** | **Article # 5 Cooper** | **Article # 6 Scholes** | **Article # 7 Deering** |
| a) | Trainees are required to activate their relevant prior knowledge and experience |  |  |  |  |  |  |  |
| b) | Trainees are stimulated to connect their past experience to new ideas, skills, and attitudes they are expected to learn |  |  |  |  |  |  |  |
| c) | Trainees receive a protocol (or guidelines) that helps them organize the new things they learn |  |  |  |  |  |  |  |
| d) | Trainees have the opportunity to demonstrate knowledge, skills, and attitudes they already mastered before the training (e.g., through a pretest) |  |  |  |  |  |  |  |
| **Subscale 3) Demonstration** | | **Article # 1 Andriguetti** | **Article # 2 Birch** | **Article # 3 Chichester** | **Article # 4 Clark** | **Article # 5 Cooper** | **Article # 6 Scholes** | **Article # 7 Deering** |
| a) | Trainees are given demonstrations of the skills and/or models of the behaviours they are expected to learn |  |  |  |  |  |  |  |
| b) | Trainees are given examples of errors, mistakes and things that can easily go wrong |  |  |  |  |  |  |  |
| c) | Trainees’ attention is directed to skills, information, and attitudes that are most relevant and/or most important |  |  |  |  |  |  |  |
| d) | Trainees receive multiple demonstrations that represent alternative ways of performing the skills that need to be learned |  |  |  |  |  |  |  |
| e) | Trainees receive demonstrations not as simple descriptions but in a lifelike fashion (e.g., real-life modelling, video, animation) |  |  |  |  |  |  |  |
| f) | Trainees learn the steps containing non-observable decision making and reasoning processes |  |  |  |  |  |  |  |
| **Subscale 4) Application** | | **Article # 1 Andriguetti** | **Article # 2 Birch** | **Article # 3 Chichester** | **Article # 4 Clark** | **Article # 5 Cooper** | **Article # 6 Scholes** | **Article # 7 Deering** |
| a) | Trainees have opportunities to practice or try out what is learned |  |  |  |  |  |  |  |
| b) | Trainees are tested on new, unfamiliar scenarios to see if they can apply what has been learned |  |  |  |  |  |  |  |
| c) | Trainees´ errors when working on the scenarios are detected and they receive feedback on it |  |  |  |  |  |  |  |
| d) | Trainees are required to predict challenges and/or explain causes of undesirable outcomes |  |  |  |  |  |  |  |
| e) | Trainees collaborate with peers to enhance their learning |  |  |  |  |  |  |  |
| **Subscale 5) Integration/Transfer** | | **Article # 1 Andriguetti** | **Article # 2 Birch** | **Article # 3 Chichester** | **Article # 4 Clark** | **Article # 5 Cooper** | **Article # 6 Scholes** | **Article # 7 Deering** |
| a) | Trainees have the opportunity to reflect on, discuss with others, and defend what they learned |  |  |  |  |  |  |  |
| b) | Trainees have the opportunity to explore how they can personally use what they learned |  |  |  |  |  |  |  |
| c) | Trainees are able to publicly demonstrate to others what they learned (e.g., at the workplace) |  |  |  |  |  |  |  |

1. **ADDITIONAL INFO PPH Simulation & ID Subset**^*1^**.doc**

*1 – each article’s table subset varied according to the subset each rater was allocated to;

1. **ADDITIONAL INFO PPH Simulation & ID Subset1to7**

| **ADDITIONAL INFO** | |
| --- | --- |
| **Article #1 Andriguetti - Additional information** | |
| **Study Aim** | The purpose of this project was to determine whether the use of high-fidelity simulations increased student confidence in managing the obstetric emergencies of shoulder dystocia and PPH. |
| **Study design** | This project used a quasiexperimental design to evaluate student confidence in learning the management of the obstetric emergencies of shoulder dystocia and PPH. Student confidence was measured immediately before and after the learning experiences. Two groups of students were studied. A control group (n=10) received standard teaching methods consisting of class discussion, watching a video, and low fidelity teaching methods. An intervention group (n=18) received a high-fidelity simulation learning experience. In both the control and intervention groups, students were randomly assigned to participate in either a shoulder dystocia or a PPH simulation. A fixed randomization process was used to ensure that there were equal numbers of students in the shoulder dystocia and PPH groups. |
| **# of trainees** | Total of 18 participants. The control group (n = 10) received standard teaching methods consisting of discussion, watching a video, and low-fidelity teaching methods. The intervention group (n = 18) received a high-fidelity simulation learning experience. |
| **Trainees' field of practice & level of expertise** | The participants were registered nurses enrolled in a graduate midwifery education program in the Southeastern United States. This study was conducted at a graduate level midwifery education program in the Southeastern United States. The participants were all registered nurses enrolled in the program. All students (n = 28) attending simulation learning sessions between November 2009 and March 2010 were invited to participate, and all consented to be part of the study. All of the students had completed or were near completion of their didactic course work and were preparing to enter their clinical practicums. |
|  | |
| **Article # 2 Birch - Additional Information** | |
| **Study Aim** | This study was developed to explore the best way of delivering obstetric emergency drill training.  Simulation based training may not be the best method for knowledge acquisition. However, this study aims to identify if it carries any further benefits in the preparation of clinical staff for future practice that a classroom based approach would not. |
| **Study Design** | Three teaching methods were employed. Lecture based teaching (LBT), simulation based teaching (SBT) or a combination of these two (LAS). Each team of staff were randomly allocated to undertake a full day of training in the management of Post Partum Haemorrhage utilising one of these three teaching methods.  A nurse who was not connected with the research prepared six sealed envelopes with a teaching method written inside. The teaching method enclosed within was then allocated to that particular day. |
| **# of trainees** | In total, six teams, each containing six staff took part in the study. The number of teams was limited due to allocation of registrars. Increasing the number of teams would have necessitated running the research over several years or involving more hospitals in the study. Each team of six people consisted of one registrar, one senior house officer, a delivery suite co-ordinator, one newly qualified midwife and two team midwives. |
| **Trainees' field of practice & level of expertise** | Multi-disciplinary teams were randomly selected from obstetric and midwifery staff to participate in one day of training on an obstetric emergency topic. |
|  | |
| **Article # 3 Chichester** | |
| There was no relevant additional information for Paper#3 | |
|  | |
| **Article #4 Clark - Additional Information** | |
| **Study Aim** | In developing our simulation, we aimed to create a relevant, challenging, immersive experience that was focused on learning and enhanced by the use of appropriate technology. |
| **Trainees' field of practice & level of expertise** | A few years ago, the Labor and Delivery (L&D) Unit and Department of Obstetrics and Gynecology at the University of Utah started to examine how to improve our team functioning in emergency situations. In 2007 to 2008, a multidisciplinary team of obstetricians, anesthesiologists, labor and delivery nurses, and ancillary staff initiated team training exercises in PPH. The team training concept was relatively new to our staff and the L&D Education Committee carefully outlined the agenda to ensure success. |
|  | |
| **Article #5 Cooper - Additional Information** | |
| **Study Aim** | The aim of this study was to examine the ability of student midwives to assess and manage maternal deterioration, in a simulated environment, using measures of knowledge, situation awareness and skill performance. |
| **Study Design** | An exploratory quantitative analysis of student performance based upon performance ratings derived from knowledge tests and observational ratings. |
| **# of trainees** | Thirty-five midwifery students participated out of a population of 49 (71% response rate). |
| **Trainees' field of practice & level of expertise** | ...we examined the pre-existing knowledge and skills of Australian student midwives in video recorded simulated settings incorporating clinical and situation awareness assessments. Undergraduate (single and double degree — nursing/midwifery) and postgraduate midwifery students from three campuses of Monash University, Australia were eligible to participate in this 2010 study. |

1. **ADDITIONAL INFO PPH Simulation & ID Subset8to14**

| **Additional Info Paper # 8 Egenberg2015** | |
| --- | --- |
| **Study Aim** | The aim of this study was to investigate whether the simulation training might have influenced the rate of RBC transfusions. |
| **Study Design** | Two cohorts were compared retrospectively using a pre–post design. The frequency of women receiving RBC transfusions was the primary outcome measure regarded as a surrogate marker for the actual blood loss after birth, and secondary outcomes were the frequencies of curet- tage, B-Lynch sutures, uterine embolization, and hysterectomy. |
| **# of trainees** | In 2010, the hospital implemented a yearly, mandatory, 6-h inter-professional training for 180 midwives, doctors, and nurse assistants in the maternity wards. |
| **Trainees' field of practice & level of expertise** | ... midwives, doctors, and nurse assistants in the maternity wards. |
|  | |
| **Additional Info Paper # 9 Fialkow** | |
| **Study Aim** | This article describes the development, content validation, and in situ implementation of a standardized patient-based, interdisciplinary PPH scenario. |
| **Study Design** |  |
| **# of trainees** | This simulation involved nurses (n = 49), obstetrical residents (n = 19), obstetrical attending physicians (n = 9), anesthesiology residents (n = 3), and anesthesiology attending physicians (n = 5). Although teams can vary in size and still successfully complete the scenario, optimal team com- position consists of 6 to 8 individuals with expertise from all 3 disciplines/specialties (nursing, obstetrics, and anesthe- sia.) A total of 83 learners at the University of Washington Medical Center Labor and Delivery (L&D) unit completed the scenario. |
| **Trainees' field of practice & level of expertise** | This simulation involved nurses (n = 49), obstetrical residents (n = 19), obstetrical attending physicians (n = 9), anesthesiology residents (n = 3), and anesthesiology attend- ing physicians (n = 5). Although teams can vary in size and still successfully complete the scenario, optimal team com- position consists of 6 to 8 individuals with expertise from all 3 disciplines/specialties (nursing, obstetrics, and anesthe- sia.) A total of 83 learners at the University of Washington Medical Center Labor and Delivery (L&D) unit completed the scenario. |
|  | |
| **Additional Info Paper # 10 Magee** | |
| **Study Aim** | Our goal in conducting this research was to study the feasibility and effectiveness of low-cost simulation for teaching residents to manage obstetrical emergencies and retain information over time regarding specifics of management, specifically for preeclampsia/eclampsia and postpartum hemorrhage. |
| **Study Design** | Residents were randomly assigned to intervention or control group. Within each group, residents were assigned to learn about postpartum hemorrhage or preeclampsia/eclampsia; we chose these two scenarios based on learning needs identified by the faculty. |
| **# of trainees** | We recruited 20 second-year residents (family medicine) over 18 months during their required labor-and-delivery month. |
| **Trainees' field of practice & level of expertise** | Twenty residents were randomly assigned to the intervention (simulated PPH or PEC followed by debriefing) or control (lecture on PPH or PEC) group, and equal numbers of residents were assigned to each scenario. |
|  | |
| **Additional Info Paper # 11Markova** | |
| **Study Aim** | In the present study we evaluated the effects that this form of multi-professional obstetric skills training had on the incidence and causes of severe PPH. To estimate this, we performed an audit of medical records and used transfusion rates and the individual requirements for red blood cell (RBC) unit transfusions as indicators in three groups of patients with PPH representing the period before, during and after implementation of the multi-professional obstetric skills training. We also evaluated the pre- and post-transfusion hemoglobin levels to estimate any change in transfusion decision practice, and the compliance with national and hospital transfusion guidelines. Both vaginal deliveries and cesarean sections (CS) were evaluated.  We hypothesized that multi-professional obstetric skills training could reduce the number of women who suffered excessive bleeding and therefore received RBC transfusions, and that this would be reflected in transfusion rates and requirements. |
| **Study Design** |  |
| **# of trainees** | The training sessions were (and still are) held regularly to include all staff, and members are invited to repeat the course every two to three years. The first session with a small minority (*n*=12) of the staff (*n*=156) was provided 9 December 2003 – the only session in 2003. In 2004 an increasing number of staff participated, and in 2005 a total of about 95% of staff had participated in the obstetric skills training. |
| **Trainees' field of practice & level of expertise** | Midwives, nurses, auxiliary nurses and doctors on call participated. |
|  | |
| **Additional Info Paper # 12 Marshal** | |
| **Study Aim** | Our goals were to: (1) improve PPH recognition time, (2) improve medical management response times and (3) decrease medication errors via improved team communication. |
| **Study Design** | We conducted a community/rural hospital multi-center longitudinal study to evaluate in situ simulation and team training for PPH. |
| **# of trainees** | Twenty-two clinical teams consisting of one maternity provider (obstetrician, family physician or certified nurse midwife) and two obstetric nurses completed PPH simulations before and 9–12 months after team training. Clinical team characteristics are summarized in Table 1. Participating clinical teams came from six community/rural hospitals. The majority of the providers had been involved in a delivery within the past week (77.3%, n1⁄417), although eight had attended 520 deliveries in the past 6 months. |
| **Trainees' field of practice & level of expertise** | See Table 1. |
|  | |
| **Additional Info Paper # 13Maslovitz2007** | |
| **Study Aim** | We initiated an experimental project that applies simulation technology to hands-on training of delivery room teams in managing extreme obstetric situations. A simulation-based course is supposed to reflect reality. Our rationale was that inaccurate techniques and management errors spotted during a full-scale simulation would be the same as those that occur repeatedly in real life. Our intent was that these errors would then provide the material for a simulation- based interventional program aimed at improving the ability of the teams to perform correctly in extreme situations such as those that might occur in the clinical setting. |
| **Study Design** |  |
| **# of trainees** | Between February 2004 and April 2006, 60 residents in obstetrics and gynecology and 88 midwives underwent the simulation-based course. Forty-two labor and delivery teams completed all four sessions. All residents were in the first 3 years of a 6-year program: 14 (23%) in their first year (after 6 months of training at labor and delivery), 34 (57%) in their second year, and the remaining 12 (20%) in their third year of residency. The midwives had a mean delivery room experience of 2.5 years (range 1– 8 years). The demographic characteristics of the trainees are shown in Table 1 |
| **Trainees' field of practice & level of expertise** | Each team consisted of at least one resident in obstetrics and gynecology and two midwives. |
|  | |
| **Additional Info Paper#14 Maslovitz2008** | |
| **Study aim** | We aimed at assessing the accuracy of estimated blood loss by obstetrical teams during a simulated Postpartum hemorrhage (PPH) scenario. |
| **Study Design** | a prospective study conducted as part of the simulation-based training course, using sophisticated mannequin simulators adapted for obstetrical training by specially designed devices. Setting. Part of the simulation-based training course. |
| **# of trainees** | During the study period (July 2003 May 2006), 42 teams (68 physicians, 80 midwives) participated in the regular blood loss evaluation study. |
| **Trainees' field of practice & level of expertise** | Most of the participating residents had not completed their third year of residency. The midwives were more evenly distributed across years of experience (Table I). |

1. **ADDITIONAL INFO PPH Simulation & ID Subset15to21**

| **Additional Info Paper # 15 Nellisen** | |
| --- | --- |
| **Study Aim** | The aim of this study was to evaluate HMS BAB training by addressing the following research questions: (i) To what extent is HMS BAB training acceptable and feasible in a low-resource setting? (ii) To what extent do knowledge, skills, and confidence of health care providers change after HMS BAB training? |
| **Study Design** | Educational intervention study. |
| **# of trainees** | Four master trainers trained eight local facilitators, who subsequently trained 89 learners.  Four master trainers trained eight local facilitators. The group of facilitators consisted of one medical attendant, four nurse-midwives (two of whom were tutors in the midwifery school), and three clinicians. The majority of facilitators (63%) were active birth attendants at the time of training. |
| **Trainees' field of practice & level of expertise** | Clinicians, nurse-midwives, medical attendants, and ambulance drivers involved in maternity care.  Local facilitators trained 89 learners according to the train-the-trainer model (Figure 2). The number of learners per facilitator ranged from three to six. The group of learners included six ambulance drivers, 13 medical attendants, 60 nurse-midwives, and 10 clinicians. Half of the learners (46%) were active birth attendants at the time of training. Thirty percent of all learners had received in- service training of AMTSL, and 18% had received train- ing on management of PPH during their career to date (Table 1). |
|  | |
| **Additional Info Paper # 16Phillippi** | |
| **Study Aim** | The purpose of this manuscript is to describe the development and implementation of an interprofessional simulation for nurse-midwifery and nurse-anesthesia students designed to increase interprofessional communication and collaboration. The goal is to provide information for educators and nurse-leaders to create interprofessional simulations to meet the Core Competencies for Interprofessional Collaborative Practice. |
| **Study Design** | The interdisciplinary simulation was designed jointly by the nurse-anesthesia and nurse-midwifery faculty to provide students with a realistic, complex experience that required interaction with other healthcare providers to resolve an ongoing patient crisis. |
| **# of trainees** | The simulation was performed three times in 2012 with a total of 29 students, 22 nurse-midwifery students, three nursing students, and four nurse-anesthesia students. In 2013 it was performed three times with a total of 21 midwifery students and six anesthesia students. There were more nurse-midwifery students in each simulation in part related to difficulties in scheduling a time that the nurse-anesthesia students were on-site for classes and had enough clinical knowledge to meaningfully participate in management of the patient scenario |
| **Trainees' field of practice & level of expertise** | nurse-midwifery and nurse-anesthesia students, |
|  | |
| **Additional Info Paper # 17Robertson** | |
| **Study Aim** | We sought to test the effectiveness of the CTT approach to train providers who respond to obstetric emergencies. We hypothesized that participation in training would positively impact 10 variables: individual’s knowledge about team process and obstetric emergency care; confidence and competence in handling obstetric emergencies; participant attitudes (toward the utility of a rapid response team, simulation technology as a teaching methodology, the utility of team skills in the workplace, comfort in assuming team roles, and about individual and team performance). Objectively measured team performance in a simulated environment was also assessed. |
| **Study Design** | Twenty-two perinatal health care professionals (attending physicians, nurses, resident, and nurse midwives) volunteered to participate in this pretest-posttest study design. |
| **# of trainees** | Twenty-two perinatal health care professionals (attending physicians, nurses, resident, and nurse midwives)... |
| **Trainees' field of practice & level of expertise** | A volunteer sample of obstetric attending physicians, certified nurse midwives, obstetrics and gynecology residents, and perinatal nurses was recruited from a large metropolitan teaching hospital in the southeast. Eligibility criteria include professionals who perform inpatient obstetric clinical duties within the health system, and respond to obstetric crisis events. Participants are part of the health system as an employee, a medical student, or a member of the obstetrics and gynecology residency program. |
|  | |
| **Additional Info Paper # 18 Crofts** | |
| **Study Aim** | The objectives of this paper were to explore the effect of multiprofessional obstetric emergency training on knowledge, as indicated by Multiple-Choice Questionnaire (MCQ) scores, and to investigate whether changes in knowledge were influenced by teamwork training or by the location of training (local hospital versus simulation centre training). |
| **Study Design** | This was a prospective randomised controlled trial, as part of the wider Simulation and Fire-drill Evaluation (SaFE) study. |
| **# of trainees** | All midwives (including those working in hospital or the community) and all doctors, working within the Obstetric Department (including general practice trainees, obstetric and gynaecology trainees and consultants) of the six participating hospitals were eligible for inclusion in the study. Staff were not eligible for randomisation to the study if any of the following criteria were present: (1) participation at a nationally accredited obstetric emergency management course within the 12 months prior to randomisation, (2) already booked to attend an accredited training course within the duration of the study, (3) participation in the pilot phase of the study, (4) involvement in the delivery of the training interventions or evaluations, (5) if on maternity or long-term sick leave and (6) consultants working solely in gynaecology with no emergency obstetric commitments.  At the commencement of the study, a total of 975 staff were working within the maternity departments in the six participating hospitals, of whom 912 were eligible for inclusion and randomised. From the 24 stratified and randomised lists of eligible staff, the first 240 were approached, with 158 giving consent to participate in the study. Eighteen staff subsequently withdrew from the study prior to the first evaluation. |
| **Trainees' field of practice & level of expertise** | Participant staff were recruited from six District General Hospitals in the South West of England, UK. The delivery rates ranged from 2500 to 4600 per annum. |
|  | |
| **Additional Info Paper # 19Siassakos** | |
| **Study Aim** | The purpose of our study was to assess the utility, content validity and application of techniques that are used in aviation, for the qualitative analysis of team communication in a ‘low fidelity’ simulated obstetric emergency scenario before and after clinical training. We then tested the construct validity of the framework by quantitatively comparing the communication patterns of drill teams that received extra teamwork training with teams that did not. We also evaluated consequential validity by associating communication problems with clinical errors. |
| **Study Design** | We studied a random sample of four teams of doctors and midwives who participated in a videotaped simulated obstetric emergency, postpartum haemorrhage, before and after ‘on-site’ clinical training |
| **# of trainees** | The participants were a stratified random sample of four groups (one senior, one junior doctor and two senior, two junior midwives per group). We studied a random sample of four teams of doctors and midwives... |
| **Trainees' field of practice & level of expertise** | The participants were a stratified random sample of four groups (one senior, one junior doctor and two senior, two junior midwives per group). We studied a random sample of four teams of doctors and midwives... |
|  | |
| **Additional Info Paper # 20 Straub** | |
| **Study Aim** | In our institution, we have developed an introductory orientation programme for our incoming residents, in which we utilise a obstetric skills. Cervical examination, routine vaginal delivery and perineal laceration repair are included in this introductory orientation. In addition, we have also incorporated a curriculum on obstetric emergencies. We hypothesise that an educational programme that incorporates high-fidelity simulation in postpartum haemorrhage will increase resident confidence and clinical knowledge in the management of this emergency and thus allow for improved performance and learning in the future. |
| **Study Design** | An educational programme consisting of a lecture and high- fidelity simulation exercise was given to incoming obstetrics and gynaecology (OB) and family medicine (FM) residents. |
| **# of trainees** | A total of 27 residents completed the pre- and post-test confidence survey: 17 OB and 10 FM residents. All of the OB residents and 27% (3/11) of the FM residents were graduates of US medical schools. Of the FM residents graduating from non-US medical schools, most were from medical schools in the Caribbean (6/8), one from a medical school in Europe and one from a medical school in Asia. |
| **Trainees' field of practice & level of expertise** | All first year obstetrics and gynaecological residents (OB) and family medicine (FM) residents at the NorthShore University HealthSystem, an integrated healthcare system and affiliate of University of Chicago Pritzker School of Medicine, participated in an educational programme as part of the incoming resident orientation in June 2010 and June 2011. |
|  | |
| **Additional Info Paper # 21Vadnais** | |
| **Title** | [Assessment of long-term knowledge retention following single-day simulation training for uncommon but critical obstetrical events.](http://www.ncbi.nlm.nih.gov/pubmed/22191668) |
| **Authors** | Vadnais MA, Dodge LE, Awtrey CS, Ricciotti HA, Golen TH, Hacker MR. |
| **Journal & Publishing information** | J Matern Fetal Neonatal Med. 2012 Sep;25(9):1640-5. doi: 10.3109/14767058.2011.648971. Epub 2012 Apr 25. |
| **Abstract** | **Objective—**The objectives were to determine (i) whether simulation training results in short- term and long-term improvement in the management of uncommon but critical obstetrical events and (ii) to determine whether there was additional benefit from annual exposure to the workshop.  **Methods—**Physicians completed a pretest to measure knowledge and confidence in the management of eclampsia, shoulder dystocia, postpartum hemorrhage and vacuum-assisted vaginal delivery. They then attended a simulation workshop and immediately completed a posttest. Residents completed the same posttests 4 and 12 months later, and attending physicians completed the posttest at 12 months. Physicians participated in the same simulation workshop 1 year later and then completed a final posttest. Scores were compared using paired t-tests.  **Results—**Physicians demonstrated improved knowledge and comfort immediately after simulation. Residents maintained this improvement at 1 year. Attending physicians remained more comfortable managing these scenarios up to 1 year later; however, knowledge retention diminished with time. Repeating the simulation after 1 year brought additional improvement to physicians.  **Conclusion—**Simulation training can result in short-term and contribute to long-term improvement in objective measures of knowledge and comfort level in managing uncommon but critical obstetrical events. Repeat exposure to simulation training after 1 year can yield additional benefits.  **Keywords**: Long-term follow up; medical education; obstetric emergencies; simulation |
| **Study Aim** | The aim of this study was to determine whether single-day, intensive, multiple-task simulation training results in short-term and long-term improvement in objective measures of knowledge and self-reported comfort level in managing uncommon but critical obstetrical events. In addition, we sought to determine whether additional knowledge and comfort could be gained from a second exposure to the same simulation workshop, 1 year following initial exposure. |
| **Study Design** | **Methods—**Physicians completed a pretest to measure knowledge and confidence in the management of eclampsia, shoulder dystocia, postpartum hemorrhage and vacuum-assisted vaginal delivery. They then attended a simulation workshop and immediately completed a posttest. Residents completed the same posttests 4 and 12 months later, and attending physicians completed the posttest at 12 months. Physicians participated in the same simulation workshop 1 year later and then completed a final posttest. Scores were compared using paired t-tests. |
| **# of trainees** | The initial simulation workshop was offered to 43 attending obstetricians and all 20 resident physicians in our department; 100% of both the resident and attending physicians participated in the initial workshop and completed all four simulation stations. |
| **Trainees' field of practice & level of expertise** | ...to 43 attending obstetricians and all 20 resident physicians in our department.. |

1. **ADDITIONAL INFO PPH Simulation & ID Subset22to28**

| **Additional Info Table Paper #22 to #28** | |
| --- | --- |
|  | |
| **Paper #22 Kato Additional information** | |
| **Study Aim** | Therefore, the purpose of this study was to determine if simulation training of PPH obstetric emergencies leads to improvement and maintenance of midwives' knowledge and performance of simulated PPH emergencies compared to no training |
| **Study design** | The study was a RCT comparing simulation training group versus no training group using a pretest-intervention-posttest design. |
| **# of trainees** | Eighty-one midwives |
| **Trainees' field of practice & level of expertise** | Inclusion criteria were midwives who: 1) had two or three years of clinical experience, 2) worked in an obstetrics ward, and 3) had experience with birth assistance. |
|  | |
|  | |
| **Paper #23 DeMelo Additional Information** | |
| **Study Aim** | The aim of the present study was to test the hypothesis that a PPH simulation training program based on instructional design guidelines would lead to better learning outcomes than the use of a simulation training program based on best practice. |
| **Study Design** | A pretest–post- test non- equivalent groups study was conducted.. |
| **# of trainees** | Of all eligible residents, half worked at the Intituto de Medicina Integral Prof. Fernando Figueira. Therefore, residents from this center formed the entire instructional design group (n=36). They were divided into 13 teams: four teams of two individuals and nine teams of three individuals. One participant had to leave the instructional design group for personal reasons before undergoing the post- test. Participants assigned to the best practice group (n=18) came from the other four maternity hospitals and underwent training at Hospital das Clínicas, Universidade Federal de Pernambuco. |
| **Trainees' field of practice & level of expertise** | Obstetrics and gynecology residents working at any of the five local teaching hospitals in Recife were considered eligible and invited to participate. These hospitals provide high- risk maternity services and report an equivalent number of deliveries per resident. |
|  | |
| **Paper #24 Egenberg Additional Information** | |
| **Study Aim** | The first aim of the study was to investigate whether interprofessional simulation training on management of PPH is associated with perceived changes in self-efficacy and collective efficacy. We hypothesised that such training would be associated with positive changes in perceived efficacy. The second aim was to test whether interprofessional simulation training on PPH is associated with a reduced blood transfusion rate after birth. We hypothesised that it would be associated with an overall reduction in blood transfusion rates. |
| **Study Design** | The study had a multimethod, quasi-experimental pre-post design that combined patient outcome with survey measures. |
| **# of trainees** | Out of a total of 104 staff members, 82 (79%) attended the first 8-hour simulation training in May 2013, while 85 (80%) out of a total of 106 staff members participated in the second training in May 2014; just 53% of staff participated in both training sessions. |
| **Trainees' field of practice & level of expertise** | The study was performed at the University Hospital of North Norway in Tromsø, being a tertiary university hospital which serves the counties of Finnmark, Troms and Nordland, and provides highly specialised treatment for citizens in the North Norway region. The hospital has approximately 1400 births/year and the staff include around 100 midwives, obstetricians, and auxiliary nurses. Midwives conduct births autonomously, assisted by auxiliary nurses. The doctor on duty is called in case of complications, like PPH, medical interventions requiring a doctor’s attendance or for advice. |
|  | |
| **Paper # 25 Nathan Additional Information** | |
| **Study Aim** | The objectives of the present study were to evaluate simulation-based training as a method for teaching postpartum hemorrhage-management skills to rural physicians and to assess the retention of the skills 2 years later. |
| **Study Design** | A quasi-experimental, pre–post intervention study was conducted at Kibogora District Hospital in the Western Province of Rwanda. |
| **# of trainees** | All 11 physicians at the study hospital completed the simulation training and eight of the original 11 participants were available for the 2-year follow-up assessment. |
| **Trainees' field of practice & level of expertise** | A convenience sample of physicians was enrolled according to the single inclusion criterion of being employed at the study hospital as a generalist physician at the time the study was conducted. The eight participants had 1–7 years of experience since graduating from medical school (Table 1) and four participants had, previously or at the time of 2-year follow- up, spent time every week working full days on the maternity ward. |
|  | |
| **Paper # 26 Higgins Additional Information** | |
| **Study Aim** | This study was carried out to examine how experienced clinicians viewed the effects of two different methods of delivering information on recognition and management of a PPH within a MoreOB workshop. |
| **Study Design** |  |
| **# of trainees** | One hundred ten participants consented to complete the questionnaire, representing 73% of 150 original workshop participants. |
| **Trainees' field of practice & level of expertise** | Mount Sinai Hospital is a tertiary level unit in Toronto, Ontario, providing obstetric care for 7500 births in an ethnically diverse population. Permanent clinical staff (nurses, midwives, obstetricians, and family doctors) are all enrolled in the MoreOB program, with annual workshops focused on preparation for obstetric emergencies. Staff members attending the workshop were invited to participate in a study evaluating different educational methods of teaching the recognition and management of PPH. All participants were exposed to the different educational interventions. As this was a mandatory session for permanent staff, the content was based on the MoreOB module on PPH. |
|  | |
| **Paper # 27 Hilton Additional Information** | |
| **Study Aim** | In this prospective observational study, we exposed 14 multidisciplinary teams to checklist training to evaluate checklist use and team performance during a simulation of major PPH post-vaginal delivery. |
| **Study Design** | This prospective observational study was approved as exempt by Stanford University Institutional Review Board. |
| **# of trainees** | Fourteen multidisciplinary teams were studied: each team consisted of seven to 10 participants which included two obstetricians (attendings, fellows, and residents), one anesthesiologist (resident), three to five labor and delivery nurses and one to two operating room (OR) technicians. |
| **Trainees' field of practice & level of expertise** | Fourteen multidisciplinary teams were studied: each team consisted of seven to 10 participants which included two obstetricians (attendings, fellows, and residents), one anesthesiologist (resident), three to five labor and delivery nurses and one to two operating room (OR) technicians. Participants were na ̈ıve to the scenario before performing each simulation. |
|  | |
| **Paper # 28 Miller Additional Information** | |
| **Study Aim** | This article describes the development and initial evaluation of hybrid simulation used for labor and birth emergency situations. |
| **Study Design** |  |
| **# of trainees** |  |
| **Trainees' field of practice & level of expertise** | The cases were developed for teams of 2 to 4 students in order to reduce student anxiety about individual performance and improve learning through collaboration in decision mak- ing and patient care. |

1. **ADDITIONAL INFO PPH Simulation & ID Subset29to32**

| **Additional Info Table Paper #29 to #** | |
| --- | --- |
|  | |
| **Paper #29 Wong Additional information** | |
| **Study Aim** | The aim of the current report is to describe the implementation of the OBHEP project and to report on change and retention in knowledge among providers (physicians, registered nurses, advanced practice nurses), as assessed by the pre- and post-tests. |
| **Study design** |  |
| **# of trainees** | Paired pre-and post-examination results were available for 9456 providers from 95 hospitals. |
| **Trainees' field of practice & level of expertise** | Hospitals were asked to list nurses on their final reporting grids in the area where they spent most of their time. Additionally, some hospitals tested clinical nurse specialists, nurse managers and supervisors, perinatal center staff and other educators. While these employees do not usually provide direct patient care, they were included in this training program because as leaders and educators they should understand the procedures and drills necessary for the education and treatment of maternal hemorrhage. These providers make up the ‘‘other’’ category, along with intensive care unit, general operating room and emergency department staff members (who often contribute to the management of maternal hemorrhage in smaller hospitals). |
|  | |
| **Paper #30 Evans Additional Information** | |
| **Study Aim** | The objectives of the present study were to validate the BAB training module, including methods, materials, and integration with simulators for ability to transfer knowledge and skills, and accept-ability to learners and facilitators, and to evaluate the psychometric properties of the learner assessment tools (construct validity using item difficulty and discrimination statistics from each of the knowledge and skills assessment). |
| **Study Design** | The study design was a pre- and post-assessment of participants in BAB training in Haldwani and Rudrapur, India, in February 2012; in Lilongwe district in Malawi, in April 2012; and in Stone Town, Zanzibar, Tanzania in July 2012. |
| **# of trainees** | In India, three groups were trained with 16, 16, and 21 participants on each day. In Malawi, 22 were trained on the first day and 23 on the second. In Zanzibar there were 27 participants on the first day and 21 on the second.  We invited 144 individuals to participate; however, 11 additional providers participated in the training for a total of 155. |
| **Trainees' field of practice & level of expertise** | In Malawi, cadres consisted of more skilled birth attendants, compared with India and Zanzibar. The proportion of participants who performed AMTSL was relatively higher in Malawi and India compared with Zanzibar. Overall, a majority (57%) of trainees had delivered between 1 and 44 deliveries in the last 90 days and a sizable proportion (40%) had attended 45 or more deliveries. Although BAB was meant for birth attendants in active practice, overall 13% had not conducted a birth in the last 90 days.  Twelve experienced maternal and newborn health trainers from the intervention countries were oriented to the BAB module, simulators, skills, assessment, standardization, and study procedures in a two-day session conducted by the principal investigator (CLE). |
|  | |
| **Paper #31 Monod Additional Information** | |
| **Study Aim** | The purpose of this study was to determine the influence of a one-day multi-disciplinary and multi-professional simulation training course for obstetrical emergencies based on the self-assessment of four specific skills: self- confidence, handling of the emergency situation, knowledge of algorithms and team communication.  We also analysed the influence of professional experience on self-perceived improvement of the four investigated specific skills 3 months after the training. |
| **Study Design** | In this observational study, six multi-professional, multi- disciplinary obstetrical simulation training courses were organized between November 2010 and March 2012 by the Department of Obstetrics in collaboration with the Departments of Anaesthesiology and Neonatology at the University Hospital of Basel, Switzerland. |
| **# of trainees** | A total of 168 participants took part in the six simulation training sessions. |
| **Trainees' field of practice & level of expertise** | The overall return rate of this questionnaire was 36.3 %. 153 participants provided information about their clinical function: 51 (33.3 %) midwives and 102 (66.7 %) obste- tricians took part in the training. 156 participants indicated their level of professional experience: 40 (25.7 %) had 0–2 years, 30 (19.2 %) 2–5 years, 34 (21.8 %) 5–10 years and 52 (33.3 %) [10 years of professional experience. |
|  | |
| **Paper # 32Highfiled Additional Information** | |
| **Study Aim** | We designed a pre-/posttest study to determine whether 1 day of nurse-led lecture plus simulation would increase obstetric/ perinatal staff nurses’ self-assessed knowledge and confidence in managing five high-risk, low-frequency situations that had been identified among our hospital’s data-based, annual perinatal safety goals. A secondary research goal was to establish preliminary reliability and validity of an economical, quantitative scale to measure simulation outcomes of self-assessed knowledge and confidence among RNs practicing in the labor/ birth and postpartum areas. Additionally, beyond answering the research questions, we hoped to learn practical strategies for conducting and evaluating simulation that might benefit us and others. As Ennen and Satin (2010) have argued, even small research projects can contribute by correcting problems in the simulation drill itself, identifying areas for future education, overcoming barriers to learner engagement, and resolving cost and space issues. |
| **Study Design** | We designed a pre-/posttest study to determine whether a day of nurse-led lecture plus low- fidelity simulation would increase registered nurses’ self-assessed knowledge and confidence in managing five high-risk obstetric/perina tal situations. |
| **# of trainees** | Thee nurse manager scheduled each of the 67 RNs to attend one of three lecture-plus-simulation, hospi- tal-required training days. |
| **Trainees' field of practice & level of expertise** | Students: Learners were experienced labor/birth and postpartum RNs with varying academic preparation and years of experience. They were familiar with patient care equipment used in the simulations. |

1. **Each rater received a pdf copy of the complete articles of their corresponding subsets. Please see Table 4 in the manuscript with the complete references for all 32 articles.**
